# Microbial and Metabolic Flexibility in Response to Habitat Disturbance in an Ecologically Specialist Primate

**DOI:** 10.1111/mec.70562

**Published:** 2026-09-27

**Authors:** Nina Beeby, Charline Rasoanarimalala, Mihary F. Rasoavolandrainy, Jessica M. Rothman, James P. Higham, Maria Luisa Savo Sardaro, Katherine R. Amato, Timothy H. Webster, Andrea L. Baden

**Affiliations:** ^1^ Department of Anthropology Hunter College CUNY New York New York USA; ^2^ Department of Anthropology The Graduate Center CUNY New York New York USA; ^3^ The New York Consortium in Evolutionary Primatology (NYCEP) New York New York USA; ^4^ Ranomafana Ruffed Lemur Project (RRLP) Ranomafana Madagascar; ^5^ Department of Sciences University of Fianarantsoa Fianarantsoa Madagascar; ^6^ Department of Animal Biology University of Antananarivo Antananarivo Madagascar; ^7^ Department of Anthropology New York University New York New York USA; ^8^ Department of Anthropology Northwestern University Evanston Illinois USA; ^9^ Department of Human Science and Promotion of the Quality of Life University of San Raffaele Milan Italy; ^10^ Department of Anthropology University of Utah Salt Lake City Utah USA

**Keywords:** gut microbiome, metabolome, nutrition, physiology, resilience, specialism

## Abstract

In the Anthropocene, understanding what renders a species prone to extinction is critical to wildlife management. Ecological specialists are hypothesised to be at particular risk, given that morphological, physiological, and/or behavioural constraints are expected to impede their responses to rapid habitat degradation. Nevertheless, studies have found mixed support for this hypothesis, raising the question, ‘how resilient are specialists to environmental change?’ Here, we test the hypothesis that ecological specialists, limited by behavioural and physiological constraints, may be at an energetic disadvantage in degraded habitats. Specifically, we tested whether Critically Endangered dietary specialist primates, black‐and‐white ruffed lemurs ( *Varecia variegata* ), living in secondary forests suffered nutritional and energetic deficits compared to those in primary forest habitats over a 12‐month period. To do this, we used mixed modelling approaches to examine relationships among behaviour, nutritional chemistry, 16S sequencing, metagenome functional predictions, metabolite profiles, and energetic outcomes. Compared to primary forest‐living conspecifics, we found that animals in the degraded forest consumed slightly fewer calories from less diverse diets. These animals exhibited less diverse gut microbiota, reduced microbial functional potential, and altered metabolomic profiles. Nevertheless, despite apparent nutritional constraints, energetic outcomes were broadly similar across habitats. These findings suggest that an organism's gut microbiome may be able to regulate microbial metabolic potential to facilitate resilience under suboptimal conditions. These findings highlight host‐microbiome interactions as an important component of resilience in ecological specialists, with broad implications for predicting species persistence amid ongoing environmental change.

## 1 Introduction

As anthropogenic disturbances increase globally, animals are facing mounting ecological stressors, increasing the likelihood of evolutionary mismatch (Stenseth and Mysterud 2002; Raubenheimer et al. 2012). Ecological specialists can be particularly vulnerable to environmental change, as their narrow niche breadth constrains the range of resources, habitats and environmental conditions they can tolerate (Futuyma and Moreno 1988; Devictor et al. 2008). While definitions of ecological specialisation can be highly variable, and largely depend on the system, organism, and question of interest (Devictor et al. 2010), specialisation has long been linked to extinction risk (McKinney 1997; Colles et al. 2009; Britnell et al. 2023). There is fairly strong support for the view that specialists are at a disadvantage when facing the combined effects of climate, land use, and land cover change (Devictor et al. 2008). Nevertheless, although ecological specialists are often assumed to be vulnerable because of narrow behavioural or dietary flexibility, resilience may emerge through compensatory mechanisms operating at other biological scales. For example, when dietary flexibility is limited, physiological or microbial plasticity may partially offset energetic costs by increasing digestive efficiency, reallocating metabolic pathways, or stabilizing host energy balance under altered environmental conditions (e.g., Sun et al. 2022; Mallott et al. 2022). Whether such compensatory mechanisms can buffer specialists against degraded habitats remains poorly understood.

Habitats are complex biological landscapes and can elicit diverse behavioural (Mazza and Šlipogor 2024), nutritional (Machovsky‐Capuska et al. 2016), metabolic, and physiological (Alberdi et al. 2016; Voolstra and Ziegler 2020) responses from the animals that inhabit them. A key connection among these processes is metabolism. The gut microbiome (GM) has been established as a major facilitator of ecological adaptability by supporting and modifying host metabolic function (Lindsay et al. 2020; Karasov et al. 2025). Given the strong relationship between the GM and host diet (e.g., Trevelline and Kohl 2022), narrow host dietary breadth may constrain the flexibility of the GM in responding to dietary change, which may help to explain specialists' vulnerability to environmental change. Nevertheless, recent studies have shown conflicting patterns of specialist versus generalist responses to disturbance‐induced habitat change (e.g., Chen et al. 2021; Koziol et al. 2023). Understanding specialisation within the context of these integrated systems may therefore help to reveal the precise drivers of extinction risk in ecological specialists.

Habitat degradation is expected to alter both the abundance and nutritional composition of available foods (Birnie‐Gauvin et al. 2017), which may constrain dietary specialists by limiting access to preferred resources. In turn, altered nutrient intake can influence gut microbial community composition and functional potential because gut microbes are highly responsive to changes in substrate availability and play central roles in digestion, fermentation, and host energy harvest (Lindsay et al. 2020). Changes in microbial metabolism may therefore mediate the extent to which nutritional perturbations translate into host energetic deficits. In particular, short‐chain fatty acids (SCFAs), compounds produced via microbial fermentation of carbohydrates and proteins, can be used as a direct energy source for the host (e.g., Amato et al. 2015). Under this framework, host energetic condition is not expected to respond directly to habitat degradation alone, but rather to emerge from interactions among resource availability, dietary behaviour, microbial function, and host metabolism.

Here, we compared two populations of black‐and‐white ruffed lemurs ( *Varecia variegata* ), Malagasy rainforest specialists (Morelli et al. 2020) living in [1] primary and [2] regenerating secondary habitats which differ in resource availability and diet selection (Beeby et al. 2023; Beeby 2025). We tested whether GM plasticity may contribute to buffering against environmental and nutritional challenges in an ecological specialist. We predicted that animals inhabiting secondary forest would experience reduced access to preferred foods, resulting in lower dietary diversity and lower calorie intake due to their strong fruit preference and nutrient specialism (*sensu* Machovsky‐Capuska et al. 2016; Beeby et al. 2023). We further predicted that altered nutrient intake would be associated with shifts in GM diversity, composition, and putative metabolic function (Donohue et al. 2019; Greene et al. 2020; McManus et al. 2021; Beeby et al. 2025). Finally, we predicted that if microbial or physiological flexibility compensates for upstream nutritional constraints, animals in secondary habitat would maintain energetic condition despite nutritional and microbial differences, consistent with metabolic buffering. Alternatively, if such compensatory mechanisms are insufficient, we predicted that animals in secondary habitat would exhibit reduced energetic condition relative to animals in primary forest. By integrating behavioural, nutritional, microbial, metabolomic, and physiological data, this study provides a multifactorial mechanistic framework for evaluating resilience in ecological specialists under habitat degradation.

## 2 Methods

### 2.1 Study Site and Subjects

We collected data on two populations of black‐and‐white ruffed lemurs ( *Varecia variegata* ) in Ranomafana National Park, Madagascar (RNP), from August 2022–July 2023. Mangevo [21°23′00′ S, 47°27′30″ E] is a primary rainforest site in the southeast of RNP and Talatakely [21°15′37′ S, 47°25′36″ E] is a recovering, secondary growth rainforest site previously subject to logging (White et al. 1995; Balko 1998). Study subjects at Mangevo (MGV) comprised *N* = 20 adult individuals with ID‐tagged radio‐collars. Talatakely (TLK) has only recently been recolonised by ruffed lemurs, and as such, study subjects at this site comprised *N* = 5 adult individuals (3 with ID‐tagged GPS‐collars plus 2 non‐collared animals).

### 2.2 Data Collection

#### 2.2.1 Ecological Monitoring

We used data collected by phenology teams for selected ruffed lemur food trees along established transects (MGV: 5 × 500 m, *N* = 275 trees; TLK: 3 × 500 m transects, *N* = 195 trees; Mancini 2023), comprising measurements for flowers and buds, unripe and ripe fruit, and young and mature leaves (as per Dunham et al. 2018, Beeby et al. 2023). We calculated monthly food availability indices (FAI_m_) using the formula: FAI_m_ = Σ *P*_*xm*_, where *P*_*xm*_ is the proportion of trees with each plant part for species *x* in month *m* (adapted from Wrangham et al. 1991). We also collected daily minimum and maximum temperature (°C) as well as daily total rainfall (mm) at each site to quantify climate.

#### 2.2.2 Behavioural Observations

Two research teams simultaneously targeted individuals from both populations for daily focal animal follows from 7 am to 4 pm (limited to park opening hours). We collected all‐occurrence feeding behaviour data for nutrition, recording plant taxa, plant parts, and amounts consumed (Altmann 1974; Rothman et al. 2012; described in Beeby et al. 2023). We collected a total of *N* = 1727 h of all‐occurrence feeding data (MGV: *N* = 894; TLK: *N* = 833).

#### 2.2.3 Plant Sampling

Once per month, we targeted feeding trees of the 10 most frequently fed on plant taxa for collection (those with the greatest proportion of observed feeding time that month). Plant parts sampled included flower buds, flowers, unripe and ripe fruit, mature and young leaves. Plants were processed in the same way as the focal animals process them before recording the number of units (#), wet weight (g) before dehydration, and dry weight (g) after dehydration, then stored in Whirlpak bags. We collected a total of *N* = 261 plants from the two sites (MGV: *N* = 117, TLK: *N* = 144).

#### 2.2.4 Faecal Sampling

We collected faecal samples from focal animals during behavioural observations. Samples were collected immediately following defecation and stored in 90% ethanol at room temperature until transport to Centre ValBio (< 2 weeks), where they were stored at −20°C until shipping to the US. Note that the storage time at room temperature at MGV (mean: 6 days) was longer than for TLK (mean: < 1 day). Sample‐level storage duration was not recorded, therefore, this effect could not be directly quantified or corrected for. Several field studies have indicated that storage differences of gut microbiome samples as seen in our study do not typically obscure real biological differences, even when samples are stored at room temperature for several weeks (Song et al. 2016; Gogarten et al. 2018; Asangba et al. 2019), though this could still introduce some unaccounted‐for variation. We collected a total of *N* = 185 faecal samples (MGV: *N* = 83; TLK: *N* = 102).

#### 2.2.5 Urine Sampling

We opportunistically collected urine samples during behavioural observations. Samples were collected using a plastic bucket held under urinating animals (sterilised between uses), then measured for specific gravity using a handheld refractometer (Atago 4410 PAL‐10S) and pipetted onto filter paper (Cytiva Whatman Grade 5 Circles). Filter papers were then dried and stored in aluminium foil at room temperature until shipping to the US. We collected a total of *N* = 124 urine samples (MGV: *N* = 72; TLK: *N* = 52).

### 2.3 Data Analyses

#### 2.3.1 Nutritional Chemistry

We milled samples through a 1‐mm screen using a Wiley Mill before sequentially analysing macronutrient components (Rothman et al. 2012; detailed methods described in Beeby et al. 2023; Beeby 2025). Briefly, we measured dry and wet weight for all samples prior to analysis, then analysed samples for dry matter, ash, crude protein (CP), available protein (AP) using ADIN protocol, crude fat, neutral detergent fibre (NDF) and total non‐structural carbohydrates (TNC). Additionally, we measured water‐soluble carbohydrates using a phenol‐sulfuric acid assay with a glucose‐fructose‐galactose standard (Dubois et al. 1956; modified by Hawlena and Rothman labs), and starch using neutral‐detergent soluble carbohydrate fractionation (Hall et al. 1999; Hall 2009).

#### 2.3.2 Nutrient Intake Calculations

We combined macronutrient contents of food items with intakes collected during behavioural observations to determine nutrient intakes. We scaled up values from observation days to 13‐h days to represent the potential total feeding time (as per Beeby et al. 2023; Beeby 2025). This number corresponds to the average daylight length and active hours for this species at this location, with limited seasonal variation in daylight length. We calculated daily nutrient intake (g/day) using: Σ (amount of dry food ingested for food item × mean nutrient contents for food item). Each macronutrient was then converted into calories using standard physiological values (NRC 2003), plus a fibre digestibility coefficient of 0.255 for NDF (Beeby et al. 2023; Beeby 2025; Edwards and Ullrey 1999). We used calorie values to calculate daily metabolisable energy intakes using: ME = TNC + AP + Lipid + NDF and non‐protein energy intakes using: NPE = fat + TNC + NDF. Macronutrient intake variables (AP, NDF, fat, TNC) were discretised into low, medium, and high intake categories based on quartile distributions for downstream mixed modelling analyses.

#### 2.3.3 Microbiome Sequencing

We generated microbiome data at Northwestern University following previously established protocols (Mallott and Amato 2018), comprising amplification of the V4–V5 region of the 16S rRNA gene using 515F/926R primers (Quince et al. 2011; Parada et al. 2016; Caporaso et al. 2012). The resultant amplicons were sequenced using an Illumina MiSeq V3 platform (Caporaso et al. 2012) at the Rush University Genomics and Microbiome Core Facility.

#### 2.3.4 Microbiome Data Processing

We processed demultiplexed sequencing data in QIIME2 version 2024.5 (Bolyen et al. 2019). We trimmed primers using Cutadapt (Martin 2011), merged read pairs with VSEARCH (Rognes et al. 2016), and filtered with quality‐filter before denoising the data with Deblur (Amir et al. 2017). We used a naïve Bayes classifier in QIIME2's q2‐feature‐classifier (Bokulich et al. 2018) with SILVA v138.1 99% database (Quast et al. 2012) for taxonomy classification. We further removed all sequences found in only a single sample. We conducted rarefaction analyses for Shannon and Faith diversity metrics with QIIME2's alpha‐rarefaction tool, which stabilised at depths of 1000 and 6000, respectively, and therefore rarefied samples to 6000 sequences to calculate these two indices with QIIME2's core‐metrics‐phylogenetic plugin. We then exported diversity estimates and the ASV count table for downstream statistical analyses.

#### 2.3.5 Metagenome Functional Predictions

We used the PICRUSt2 pipeline within the PICRUSt2 plugin for QIIME 2 (Douglas et al. 2020) to predict functional profiles of 16S sequences. Metagenome functional pathway annotations were carried out using KEGG Orthologs (Kanehisa and Goto 2000) and pathway abundance annotations were carried out using MetaCyc (Caspi et al. 2018). By default, PICRUSt2 excluded all features with the nearest sequenced taxon index (NSTI) value > 2 from outputs. Mean weighted NSTI values were low and comparable between habitats (MGV: 0.136 ± 0.046; TLK: 0.119 ± 0.051), indicating that microbial communities at both sites were similarly represented in reference genome databases.

#### 2.3.6 Metabolomics and Short‐Chain Fatty Acids

Analyses were conducted on a subset of 40 faecal samples, selected to represent both sites across the full annual cycle (MGV *n* = 21 samples from 8 individuals; TLK *n* = 19 samples from 3 individuals). Metabolomics analyses were conducted at the Mass Spectrometry Core Facility of University of Illinois Chicago following previously published protocols (Gomez et al. 2015; Amato et al. 2016; Garber et al. 2019; Mallott et al. 2022). Samples were analysed for untargeted metabolomics via Quadrupole Time‐of‐Flight Mass Spectrometry (Q‐TOF MS) with a reverse‐phased column, then aligned, normalised and annotated by Agilent software. We then grouped metabolites into pathways using the SMPDB metabolites library and pathway descriptions (https://smpdb.ca/; Frolkis et al. 2010). Because short‐chain fatty acids are major products of microbial fermentation and can contribute to host energy metabolism, we quantified faecal SCFA profiles as an additional indicator of microbial metabolic output. Short‐Chain Fatty Acid (SCFA) analyses were conducted at the Proteomics Core Facility of University of Illinois Chicago. Samples were analysed using 3‐Nitrophenylhydrazine hydrochloride (3NPH) derivatisation and liquid chromatography tandem mass spectrometry (LC–MS/MS; Liebisch et al. 2019). Calibration standards were used for identification of fatty acids, which were then calculated as relative concentrations (%, w/v).

#### 2.3.7 Energetics Analyses

We used RayBiotech Rodent C‐Peptide ELISA kits (EIAM‐CPE‐1/EIAR‐CPE‐1) to measure C‐Peptide in urine samples (uCP; Beeby et al. 2023). Briefly, we reconstituted urine from filter paper via methanol extraction and ran samples in duplicate at 1:2 dilution to detect metabolite levels. We then corrected for urine specific gravity to calculate final CP concentrations. Inter‐assay variation for quality controls was 5.1% (high) and 15.9% (low), and intra‐assay variation for quality controls was 2.6% (high) and 12.3% (low).

### 2.4 Statistical Analyses

All statistical analyses were conducted in R v4.4.1 (R Core Team 2021). For further detail, see Methods in Supporting Information.

#### 2.4.1 Nutrition and Energy Intake

We compared daily metabolisable energy intake (kcal/day) across sites using Welch two‐sample *t*‐tests, including both overall and monthly comparisons. We also assessed variation in macronutrient intake using coefficients of variation (CVs) calculated for each nutrient across sites.

#### 2.4.2 Gut Microbiome Diversity and Composition

We calculated alpha diversity (Shannon entropy and Faith's phylogenetic diversity) at both sample and individual (IndividualID) level. Site‐level differences were assessed using non‐parametric Wilcoxon tests. We generated Jaccard and Bray‐Curtis distance matrices and assessed site‐level differences using PERMANOVA in ‘vegan’ (Oksanen et al. 2013). Because the two populations differed substantially in sample size, we performed repeated balanced subsampling analyses for alpha and beta diversity in which 5 MGV individuals were randomly selected without replacement and compared to the 5 TLK individuals across 10,000 iterations. The distribution of effect sizes and significance rates across iterations were used to assess the sensitivity of results to sampling composition. To evaluate whether differences in multivariate dispersion could influence these results, we conducted betadisper analyses. Additionally, to examine site‐level differences in microbial relative abundances, we fit Bayesian generalised linear mixed models (GLMMs) with Poisson errors and log links in ‘MCMCglmm’ (Hadfield 2016; Sweeny et al. 2023), with Site as a fixed effect and ASV ID, Sample ID, Individual ID, and ASV × Site as random effects.

#### 2.4.3 Functional Predictions and Metabolomics

We conducted differential abundance analyses of PICRUSt2‐derived KEGG pathways using the LinDA method with Benjamini‐Hochberg correction in ggpicrust2 (Yang et al. 2023; Zhou et al. 2022). We compared short‐chain fatty acids across sites using Wilcoxon rank‐sum tests and normalised metabolite intensities across sites using *t*‐tests with Benjamini‐Hochberg correction. To evaluate the relationship between predicted functional potential and measured metabolomic profiles, we compared PICRUSt2‐derived KEGG functional predictions with metabolomic data from matched samples (*n* = 30). We calculated Spearman rank correlations between each KEGG functional class or subclass and each metabolomic pathway across the 30 matched samples. These exploratory correlations were then visualised as heatmaps.

#### 2.4.4 Nutrient–Microbiome and Energetic Models

To test associations among nutrient intake, microbial function, and energetic condition, we fit Bayesian GLMMs with macronutrient intake variables as predictors of microbial abundances and KEGG pathway abundances. We additionally modelled urinary C‐peptide concentrations as a function of KEGG pathway abundances and normalised metabolite intensities using Gaussian GLMMs. Full model specifications and priors are provided in Supporting Information (See Methods).

## 3 Results

### 3.1 Nutritional Similarity Across Habitats

Ruffed lemurs consumed fewer calories in the secondary forest (mean_MGV_: 410 kcal, mean_TLK_: 337 kcal; *t* = 1.898, df = 212, *p* = 0.059; Figure 1), though this was non‐significant, and seasonal patterns of energy intake were similar across sites: highest from December through March and lowest from May through September. The lowest energy intakes coincided with proportional decreases in carbohydrate and increases in fat consumption (Table S1; Figure 1). Despite this, and the lower food availability and dietary diversity in the secondary forest (Beeby et al. 2026), ruffed lemurs exhibited limited nutritional flexibility; animals converged on similar nutritional profiles in both primary and secondary habitats. Ruffed lemurs consumed diets high in carbohydrates, moderate‐to‐low in fat, and low in protein, but with seasonal shifts in proportions of non‐protein energy (NPE) components (i.e., carbohydrates and fats; Figure 1).

**FIGURE 1:**
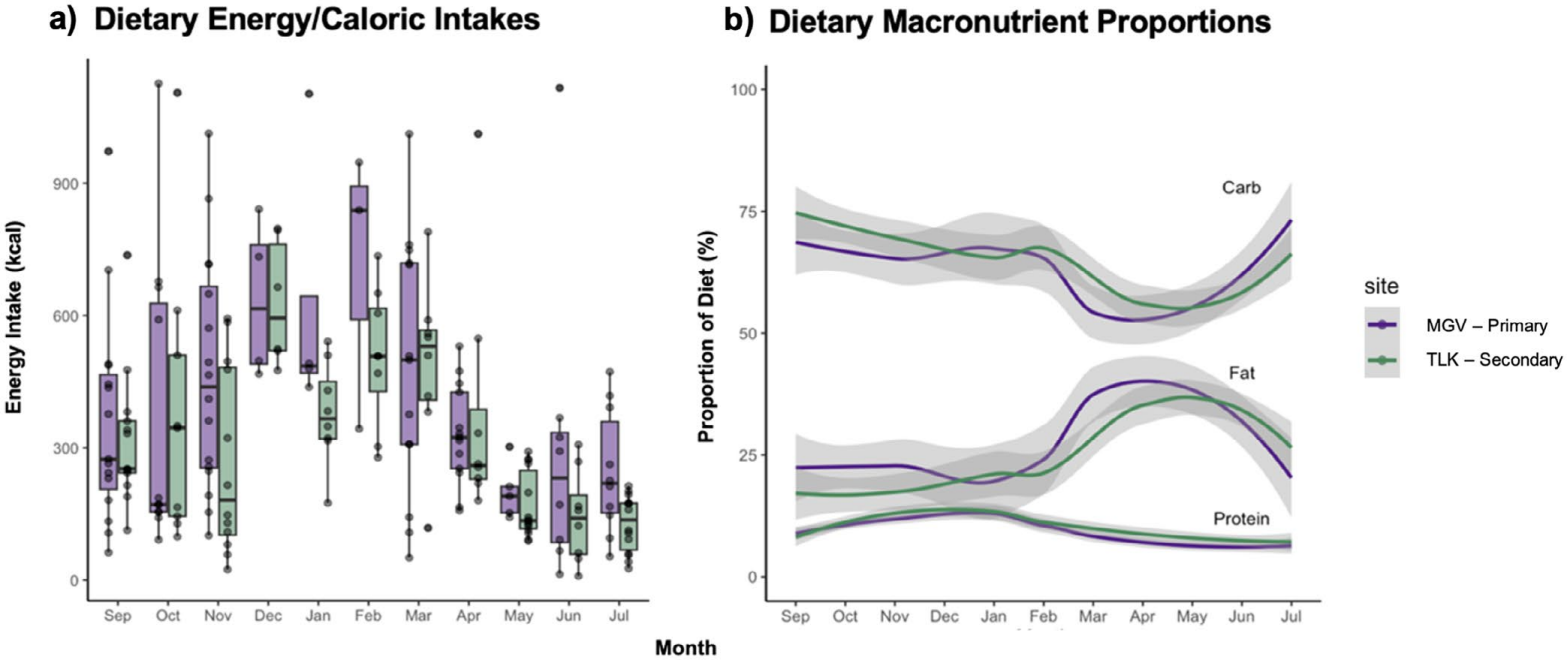
Nutrition across habitats. (a) Monthly metabolisable energy intakes (kcal) of ruffed lemurs in a primary (MGV) and secondary (TLK) forest. (b) Monthly variation in macronutrient intake proportions (expressed as % of dry matter intake).

### 3.2 Microbial and Metabolic Differences Across Habitats

Overall, the ruffed lemur population in secondary habitat exhibited moderately lower alpha diversity (Shannon Entropy and Faith PD), weakly divergent beta dissimilarity (Figure 2) and moderately different core microbiomes from those in the primary forest habitat (Figure S1). Balanced resampling analyses supported that Shannon diversity was generally higher in MGV than TLK (median difference = 0.24), though confidence intervals overlapped zero (95% CI: −0.20–0.61), and only 20.3% of comparisons were significant. Faith's phylogenetic diversity showed weaker and less consistent differences (median difference = 0.34; 95% CI: −1.42–1.85), with only 7.3% significant comparisons (Figure S2). Individual‐level stratified PERMANOVA further revealed that Site explained a small proportion of variation for both Bray–Curtis (*R* ^2^ = 0.022) and Jaccard (*R* ^2^ = 0.021) distances, with no significant effect detected under the restricted permutation scheme. No significant differences in dispersion were detected for Bray–Curtis distances (*F* = 0.143, *p* = 0.705), indicating comparable within‐group variability between sites. For Jaccard distances, a weak trend toward differences in dispersion was observed (*F* = 3.10, *p* = 0.080), suggesting slightly greater variability in presence–absence structure in one population, although this did not reach statistical significance.

**FIGURE 2:**
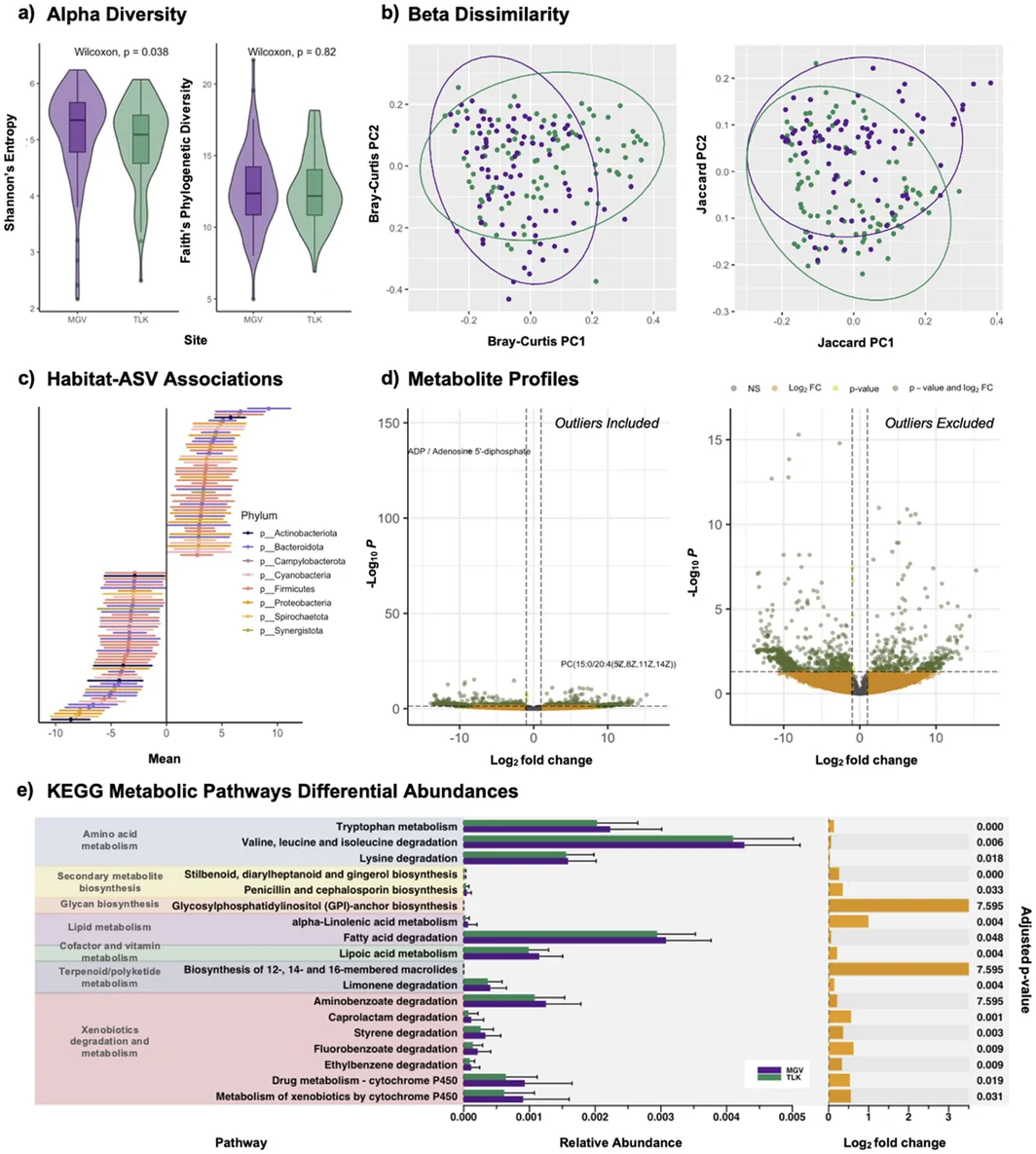
Gut microbiome, metagenome and metabolome across habitats. (a) Box over violin plots represent variation in ruffed lemur GM alpha diversity values across sites for both Shannon's and Faith's measures. (b) PCoAs represent beta dissimilarity, with centroid groupings for each site. (c) Forest plot represents point estimates and HPDIs extracted from GLMMs for the ASVs involved in the 25 strongest increases and decreases in abundance from MGV to TLK. (d) Volcano plot showing log_2_ fold changes and *p*‐values for metabolite intensities (full metabolite dataset and zoomed in y‐axis with two highest *p*‐value metabolites (labelled) from left plot excluded). (e) Greatest differential abundances across sites for KEGG metabolism pathways (18 of 18 shown).

Microbial relative abundances differed significantly across habitats (Poisson GLMM posterior mean = −0.463, ESS = 869.3, pMCMC = 0.006). The greatest differences were found within Firmicutes and Bacteroidota, the two most common phyla. Several rarer phyla present in the primary habitat were entirely absent in the secondary habitat.

Metagenome functional predictions revealed that the most abundant metabolic pathways—carbohydrate metabolism (23.1%) and amino acid metabolism (19.3%), followed by energy metabolism (13.7%), metabolism of cofactors and vitamins (11.6%), nucleotide metabolism (11.5%), and lipid metabolism (4.8%)—were comparable at both sites. However, 18 KEGG (Figure 2) and 62 MetaCyc (Figures S3 and S4) metabolic pathways exhibited significant differences across habitats, almost all of which were enriched in the primary habitat compared to the secondary habitat.

SCFA production was broadly similar across the two sites for four major SCFAs (acetate: W: 216.5, *p*: 0.655; butyrate: W: 251.5, *p*: 0.163; propionate: W: 212, *p*: 0.748; isovalerate: W: 227, *p*: 0.4644). Though not statistically significant, values were slightly higher for all SCFAs at MGV compared to TLK (Figure S5). Metabolomic analyses, however, demonstrated substantial metabolic differences across habitats (Figure 2). Of the 61,608 metabolite compounds identified, 1430 compounds representing 88 metabolic pathways exhibited significantly different intensities between primary and secondary habitat (Figure S6; Table S2). Notably, in the secondary forest, metabolites of fatty acid oxidation exhibited lower intensities and metabolites of starch and sucrose metabolism exhibited higher intensities (TLK; Figure S6).

### 3.3 Associations Among Nutrient Intake, Microbial Metabolism, and Energetic Outcomes

We first examined associations among nutrient intakes, microbial composition and microbial functional profiles. Overall ruffed lemur GM community composition did not significantly vary across nutrient intake categories (model outputs: Suppl. Results). However, estimates from posterior distributions for each random‐effect level of specific taxa revealed that NDF, TNC, and fat intakes were associated with strong shifts in specific subsets of microbial ASV abundances (e.g., several Bacteroidota and Cyanobacteria ASVs exhibited higher relative abundances under lower TNC and fat intake); AP intake was not associated with variation in any ASVs (Figure S8). Though nutrient intakes did not predict GM community composition, nutrient intakes were significant predictors of putative functional pathway abundances, with several pathways in the ‘metabolism’ class negatively correlating with associated macronutrient intakes. Available protein (AP) and fat intakes were the best predictors of overall pathway relative abundances (posterior mean_AP_ = −27,985, ESS = 1000, pMCMC = 0.049; posterior mean_Fat_ = −30,409, ESS = 1000, pMCMC = 0.028). However, decreases in macronutrient intakes were each associated with enrichment of different functional pathways (Figure S8). Most notably, decreased AP intake was associated with higher relative abundances of several amino acid metabolism pathways; decreased TNC intake was associated with higher relative abundances of several carbohydrate metabolism pathways; and decreased fat intake was associated with higher relative abundances of several lipid metabolism pathways (Figure S7).

We then examined associations between nutrient intakes, microbial metabolic measures, and energy outcomes across sites. Our GLMM indicated a marginal effect of site on energy balance (C‐peptide ng/mL; CP), with CP at TLK only slightly lower than at MGV (posterior mean = −4.871, ESS = 1000, pMCMC = 0.054). Energy balance showed distinct patterns across sites, with a notable increase in CP at MGV from September to December, which was absent at TLK (Figure S9; 20). Metagenome metabolism pathway abundances significantly positively predicted energy balance variation across both habitats (Gaussian GLMM posterior mean = 2.148, ESS = 460, pMCMC = 0.004). Higher pathway abundances were positively associated with higher C‐peptide concentrations, with model effects explaining a moderate proportion of total variance (proportion of variance: 22.2%, CI 10.1%–34.4%). Metabolite intensities also significantly positively predicted energy balance variation (Gaussian GLMM posterior mean = 0.006, ESS = 945, pMCMC < 0.001). Higher metabolite intensities were positively associated with higher C‐peptide concentrations, with model effects explaining a high proportion of total variance, though with wide confidence intervals (proportion of variance: 73%, CI 7%–96%). Heatmaps of correlation matrices for metagenome and metabolite pathways show some positive correlations across pathways, but due to the high number of pathways the patterns are complex and noisy (Figures S10 and S11).

## 4 Discussion

Ecological specialism may lead to nutritional shortfalls in disturbed or degraded habitats when resource availability deviates from preferred conditions (Devictor et al. 2008; Birnie‐Gauvin et al. 2017). However, contrary to expectations, ruffed lemurs—Critically Endangered ecological specialists—in this regenerating secondary habitat were not at a substantial energetic disadvantage relative to conspecifics in primary forest. Rather, our data suggest that metabolic flexibility via rapid shifts in microbial function may play a critical role in metabolic buffering, contributing to the maintenance of energetic condition even under suboptimal environmental conditions (Figure 3).

**FIGURE 3:**
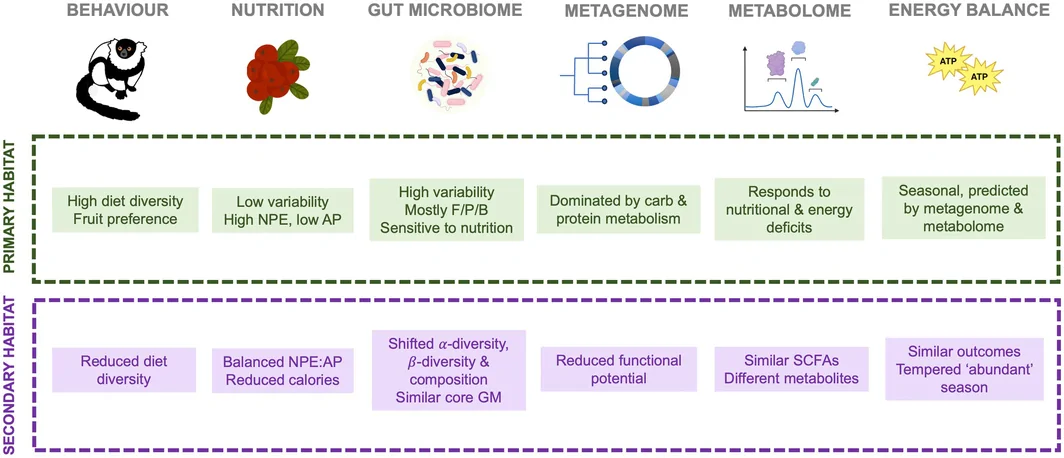
Ruffed lemur behavioural and physiological characteristics across habitats. Schematic representing behaviour, nutrition, gut microbiome, metabolome, and metagenome characteristics of ruffed lemurs in primary habitat, in addition to similarities and differences in these characteristics in degraded, secondary habitat. NPE, non‐protein energy; AP, available protein; F, Firmicutes; P, Proteobacteria; B, Bacteroidota; GM, gut microbiome; SCFAs, short‐chain fatty acids.

Despite differences in resource availability and diet selection (Beeby 2025), we found that ruffed lemurs exhibited remarkably similar patterns of nutrient balancing in both primary and secondary habitats (Figure 1). This similarity indicates that these ecological specialists are highly nutritionally constrained (*sensu* Machovsky‐Capuska et al. 2016), and may struggle to consume sufficient calories in secondary or degraded habitats without concomitant behavioural and physiological changes. However, as with some other frugivorous primates (Irwin et al. 2015; Dunham and Rodriguez‐Saona 2018; Riley et al. 2023), birds (Blendinger et al. 2022), and bats (Saldaña‐Vázquez and Schondube 2013), ruffed lemurs living in secondary forest were in calorie deficit, suggesting many host taxa are unable to maximise calorie intake if available foods are not nutritionally balanced. In this way, if degraded habitats do not offer sufficient nutritionally balanced resources for specialists, these taxa may be poorly equipped to sufficiently adapt their diets in response. Such nutritional constraints may underlie previous research findings identifying specialists as more vulnerable than generalists to environmental change (Devictor et al. 2008).

Gut microbiome (GM) flexibility has the potential to help stabilise energetic condition and facilitate host resilience in altered landscapes. Contrary to our general expectation that specialist hosts will have specialist GMs providing them limited ability to respond to dietary change, we found ruffed lemur GMs exhibited high inter‐ and intra‐individual variation (Beeby et al. 2025). We additionally found GM differences across habitat qualities, with ruffed lemurs in the secondary habitat exhibiting GM profiles characterised by moderately reduced alpha diversity, weakly divergent beta dissimilarity (Figure S2), and differing microbial relative abundances compared to those in the primary habitat (Figure 2). These patterns are similar to those found in several other primates (e.g., Barelli et al. 2015; Trosvik et al. 2018; Wasimuddin et al. 2022). However, the overall magnitude of these differences was modest, suggesting that the ruffed lemur GM may be relatively resilient to environmental change, or that host behavioural and physiological responses buffer microbial communities against habitat‐associated nutritional stress. Moreover, the secondary habitat population did not show more inter‐individual GM variation (Figure 2), nor reduced SCFA production (Figure S5), and therefore did not appear to exhibit signs of dysbiosis (Zaneveld et al. 2017). Interestingly, although metagenome functional predictions and metabolomic profiles revealed reduced functional potential in the secondary habitat, often suggested to be an indicator of decreased GM health (e.g., Clayton et al. 2018; Shi et al. 2022; Zhao et al. 2022), these functional differences occurred without evidence of extensive taxonomic turnover. Moreover, while overall pathway richness and metabolite diversity were reduced in the secondary habitat, specific metabolic pathways associated with nutrient utilisation were enriched under lower nutrient intakes at both sites, suggesting targeted functional responses rather than a uniform loss of metabolic capacity. These patterns suggest that environmental change may alter what microbial communities do more readily than which microbes are present, highlighting functional plasticity as a potential mechanism of resilience.

The GM appears to respond dynamically to variation in nutrient intakes in these populations, with decreases in specific nutrients associated with shifts in the relative abundances of microbes linked to their metabolism (Figure S7). Microbes proliferating in response to decreased nutrient intakes may reflect microbial responses that are consistent with buffering against potential energetic shortfalls by increasing metabolic efficiency for nutrients that are in short supply. We further found that macronutrient intakes negatively correlated with relative abundances of functional pathways in each nutrient metabolism class (Figure S8). This relationship is consistent with enrichment of microbial metabolic pathways under lower macronutrient intakes, potentially reflecting adjustments in microbial metabolic capacity to maximise energy harvest from those nutrients. Such relationships have been observed in another primate ( *Alouatta pigra* ; Mallott et al. 2022) but have been little investigated in other specialist taxa. However, our results indicate that GM flexibility may play a key role in modulating the effects of altered habitat conditions on specialists. Variation in nutrient intakes was associated with metagenome putative functional pathway abundances and metabolite intensities, which both in turn predicted variation in energy balance. Enrichment of putative functional pathways under decreased nutrient intakes may reflect compensatory functional shifts associated with maintenance of energy balance of the host.

Despite slightly lower calorie intake and altered microbial composition in the secondary habitat, energetic outcomes were broadly similar across sites. Moreover, both microbial functional pathway abundances and metabolite intensities positively predicted variation in urinary C‐peptide, indicating that microbial metabolic responses may be linked to host energetic stability (Mallott et al. 2022). Together, these results are consistent with a role for microbiome‐associated metabolic flexibility in mitigating the downstream energetic consequences of nutritional perturbation.

However, these findings should not be interpreted as evidence of complete energetic equivalence across habitats. The relationship between nutrient intake and microbial functional responses may also reflect feedback between host behaviour and microbial metabolism. Urinary C‐peptide was marginally lower in the secondary population (pMCMC = 0.054), and animals in secondary forest lacked the pronounced seasonal increase in C‐peptide observed in the primary population (Figure S9). This pattern may indicate incomplete energetic buffering or reduced capacity to accumulate energetic reserves during periods of resource abundance, which could have important downstream consequences for reproduction or long‐term fitness (Emery Thompson 2013). These two ruffed lemur populations appear to shift toward energy‐conserving behaviours in response to reduced food availability (Beeby et al. 2026). Such fine‐scale behavioural plasticity may alter exposure to locally available resources in ways that indirectly support microbial taxa or metabolic pathways associated with energy harvest. Under this framework, host feeding decisions and gut microbial responses may act synergistically, allowing specialists to maintain energetic stability despite altered resource landscapes. Directly investigating behaviour–microbiome feedback loops would provide valuable insight into how ecological specialists navigate environmental mismatch.

While our findings provide insight into the potential role of gut microbiome‐mediated metabolic flexibility in ecological resilience, several study design limitations should be considered when interpreting these results. Our study design necessarily compared a single primary forest site with a single regenerating secondary forest site, reflecting the logistical constraints of conducting intensive longitudinal research on Critically Endangered primates in remote locations. As a result, habitat type is partially confounded with site identity, and we cannot fully exclude the possibility that some observed differences in diet, microbiome structure, or metabolomic profiles were influenced by unmeasured site‐specific factors, such as microclimatic variation or site history, rather than habitat quality per se. Our findings should therefore be interpreted as a localised comparison of two distinct ruffed lemur populations inhabiting habitats with known differences in disturbance history, resource availability, and dietary ecology, rather than as a generalised test of all primary versus secondary forests. Nevertheless, the consistency of nutritional, microbial, metabolomic, and energetic patterns across multiple complementary datasets provides support for the broader hypothesis that microbiome‐associated metabolic flexibility may contribute to resilience in ecological specialists facing altered environments.

Together, our findings exemplify a key role for the GM in mitigating energetic and nutritional challenges faced by the host. Such metabolic flexibility may be an important component of how some animals, including ecological specialists such as ruffed lemurs, respond to altered environmental conditions. Moreover, these findings reveal the power of integrative and ‐omics approaches to answer questions in ecology. Studies using this integrative approach have the potential to identify both proximate and ultimate drivers of environmental and evolutionary mismatch for organisms inhabiting changing environments and will provide greater insight into responses to environmental change than unidimensional approaches. These methods could be used to identify the limits and thresholds of metabolic flexibility in response to different degrees and types of environmental change across both generalist and specialist taxa, including whether compensatory buffering mechanisms persist under extreme climatic events such as severe droughts, cyclones, or acute resource collapse. Moreover, from an applied perspective, these findings suggest that habitat restoration strategies that maintain or enhance dietary diversity and key functional food resources may help support gut microbial and metabolic flexibility in ecological specialists, although identifying species‐specific contributions will require further targeted experimental work.

## Author Contributions

Nina Beeby and Andrea L. Baden conceptualised and funded the study. Nina Beeby, Charline Rasoanarimalala, and Mihary F. Rasoavolandrainy conducted the data collection. Jessica M. Rothman, James P. Higham, Katherine R. Amato, Timothy H. Webster, and Andrea L. Baden contributed to methodology, resources, and training. Nina Beeby and Maria Luisa Savo Sardaro conducted formal analyses. Nina Beeby produced visualisations. Nina Beeby and Andrea L. Baden prepared the original draft, and all authors participated in the editing process and approved the final manuscript.

## Funding

Funding was provided by the American Society of Primatologists (NB), International Primatological Society (NB), Columbus Zoo and Aquarium (ALB), National Science Foundation (NB, ALB; #BCS‐(DDRI)2,216,551), and Re:Wild (NB).

## Ethics Statement

Research complied with all laws and guidelines set forth by ANGAP/Madagascar National Parks and Hunter College IACUC (#AB‐Canopy Project 5/23) and adhered to the American Society of Primatologists (ASP) Principles for the Ethical Treatment of Non‐Human Primates.

## Conflicts of Interest

The authors declare no conflicts of interest.

## Supporting information


**Figure S1:** Heat maps representing the core microbiome from ruffed lemurs at primary (MGV) and secondary (TLK) forest sites in Ranomafana National Park, Madagascar, based on a detection threshold of 1% and prevalence of 50% that is, ASVs with relative abundances of at least 1% in at least 50% of samples. Boxes around taxonomy labels indicate ASVs that are shared across the core microbiomes of the two sites, with colour representing phylum classification. ASVs that are unclassified at the genus level are given numbers for differentiation and identification for example, G1, G2.
**Figure S2:** Balanced subsampling (10,000 iterations) of Shannon and Faith PD alpha diversity between MGV and TLK ruffed lemur populations, controlling for unequal sample sizes.
**Figure S3:** Characterisation of MetaCyc predicted functional pathways highlighting the greatest differential abundances across sites for MetaCyc metabolic pathways (top 30 of 62 shown).
**Figure S4:** Heatmap showing relative abundances of MetaCyc metabolic pathways with *Z*‐scores based on CLR‐transformed relative abundances, and PCA showing differences in overall functional pathway abundances across two forest sites (MGV and TLK) in Ranomafana National Park, Madagascar.
**Figure S5:** Short‐chain fatty acid (SCFA) differences across two forest sites (MGV and TLK) in Ranomafana National Park, Madagascar.
**Figure S6:** Log_2_‐fold changes in metabolite intensity grouped by KEGG Pathway across two forest sites (MGV and TLK) in Ranomafana National Park, Madagascar. Positive values represent greater intensities at TLK, and negative values represent lower intensities at TLK, compared to MGV.
**Figure S7:** Differential abundances of microbial ASV abundances in response to nutrient intakes from ruffed lemurs in Ranomafana National Park, Madagascar, calculated from GLMMs. Violin plots represent the distribution of effect estimates for each nutrient, with point sizes representing inverse variance of estimates and rectangles indicating microbial ASVs with the greatest (positive or negative) differential abundances.
**Figure S8:** Differential abundances of KEGG functional pathway predictions (metabolism pathway class) in response to nutrient intakes from ruffed lemurs in Ranomafana National Park, Madagascar, calculated from GLMMs. Violin plots represent the distribution of effect estimates for each nutrient, with point sizes representing inverse variance of estimates and rectangles indicating KEGG IDs with the greatest (positive or negative) differential abundances (AAM, amino acid metabolism; BoSM, biosynthesis of other secondary metabolites; CM, carbohydrate metabolism; EM, energy metabolism; GBM, glycan biosynthesis and metabolism; LM, lipid metabolism; MoCV, metabolism of cofactors and vitamins; MoAA, metabolism of other amino acids; MoTP, metabolism of terpenoids and polyketides; NM, nucleotide metabolism; XDM, xenobiotics degradation and metabolism). Forest plots represent point estimates and HPDIs for the KEGG IDs involved in the 20 strongest increases and decreases of abundance from high to low nutrient intake classes (AP, available protein; NDF, neutral detergent fibre; fat; TNC, total non‐structural carbohydrate).
**Figure S9:** Monthly variation in energy balance (measured by urinary C‐peptide) of ruffed lemurs across two sites (MGV and TLK) in Ranomafana National Park, Madagascar. Adapted from (25).
**Figure S10:** Heatmap showing correlations among PICRUST2 KEGG pathway predictions and measured metabolite pathways across all samples. KEGG pathways are grouped at the KEGG Class level.
**Figure S11:** Heatmap showing correlations among PICRUST2 KEGG pathway predictions and measured metabolite pathways across all samples. KEGG pathways are grouped at the KEGG Subclass level.
**Table S1:** Monthly *t*‐test comparisons of energy intakes (kcal) across MGV and TLK sites in Ranomafana National Park. Significant values are in bold. Note: there were insufficient data collected in August to calculate monthly averages for nutrient and energy intakes.
**Table S2:** Adjusted *p*‐values, log_2_‐fold changes, *t*‐statistics and upper and lower confidence intervals for *t*‐test metabolite comparisons across MGV and TLK sites in Ranomafana National Park. Only metabolites with significant *p*‐values are included (*N* = 1430).

## Data Availability

The data that support the findings of this study are openly available in GitHub at https://github.com/thw17/Varecia_metabolomics.
