# Supplementary material for: Microbial and Metabolic Flexibility in Response to Habitat Disturbance in an Ecologically Specialist Primate
Source: Mol Ecol. 2026 Sep 27;35(18):e70562. doi: 10.1111/mec.70562 (PMC13616744; doi:10.1111/mec.70562)
Supplement: Supplementary file 1 [file MEC-35-e70562-s001.docx]

**SUPPLEMENTARY MATERIALS**

**Supplementary Results**

*Nutrient Intakes*

Protein intakes exhibited the least variation (CV_AP_: 0.85), while non-protein energy components all showed greater variation (CV_TNC_: 0.88, CV_Fat_: 0.90, CV_NDF_: 1.03). When comparing monthly energy values, we found energy intakes were significantly higher at MGV than TLK in November and July. For all other months there were no significant differences across sites (Suppl. Tab. 1).

*GM Community Composition*

At MGV, 24 phyla were identified, and GMs were dominated by Bacteroidota (35.5%), Proteobacteria (24.7%), Firmicutes (16.2%), and Cyanobacteria (12.9%; Fig. 2). At TLK, 19 phyla were identified, but GMs were dominated by the same top taxa (Bacteroidota: 36.7%, Proteobacteria: 26%, Firmicutes: 20.1%, and Cyanobacteria: 9.9%; Fig. 2). Campylobacterota, Crenarchaeota, Gemmatimonadota, Patescibacteria, and Sumerlaeota were all found in low abundances at MGV but were entirely absent from TLK. The core microbiome of each site was also similar, though not identical. We identified 11 core ASVs at MGV and 9 at TLK (Suppl. Fig. 1). ASVs which were shared between core microbiomes at both sites were all in the Prevotellaceae family of Bacteroidota (4 ASVs) and Succinivibrionaceae and Sutterellaceae families of Proteobacteria (3 ASVs).

Poisson GLMMs demonstrated that community composition differed significantly across sites (posterior mean: -0.463, ESS: 869.3, pMCMC: 0.006), though this only explained a small proportion of total variance (proportion of variance u_asv:site_: 6.4%, CI 5.7–7.2%, Fig. 2, Suppl. Fig. 2). Estimates from posterior distributions for each random-effect level across sites further revealed that ASVs demonstrating the strongest increases from MGV to TLK primarily belong to Firmicutes (36%), Bacteroidota (26%), Proteobacteria (20%), and Cyanobacteria (12%; Fig. 2). Different ASVs within these same phyla represented most of the strongest decreases from MGV to TLK also (Firmicutes: 36%, Bacteroidota: 22%, Cyanobacteria: 10%, and Proteobacteria: 10%; Fig. 2).

*Metagenome Functional Predictions*

KEGG functional predictions also revealed 18 KEGG metabolism pathways which exhibited significantly different abundances across sites, all of which were more abundant at MGV. The most relatively enriched pathways in MGV samples were associated with glycan biosynthesis, metabolism of terpenoids and polyketides, and lipid metabolism (Fig. 2). MetaCyc pathway predictions further revealed 62 pathways which exhibited significantly different abundances across sites (Suppl. Fig. 2). MetaCyc pathways most enriched in MGV samples were fatty acid β-oxidation, myo-inositol degradation, fatty acid salvage, and teichoic acid (polyglycerol) biosynthesis. The pathways most enriched in TLK samples were biotin biosynthesis and L-glutamate degradation. Differential abundance heatmaps and PCA group centroids also support that substantially more metabolic pathways were enriched at MGV compared to TLK (Suppl. Fig. 3).

*SCFA and metabolite profiles*

SCFA production was broadly similar across the two sites for all four major SCFAs (acetate: W: 216.5, P: 0.655; butyrate: W: 251.5, P: 0.163; propionate: W: 212, P: 0.748; isovalerate: W: 227, P: 0.4644). Though not statistically significant, values were slightly higher for all SCFAs at MGV compared to TLK (Suppl. Fig. 4).

We positively identified 61,608 compounds in the ruffed lemur metabolome. Of these compounds, 1,430 exhibited differential intensities across the two sites (Fig. 4; Suppl. Tab. 2), as identified by Benjamini-Hochberg corrected p-values <0.05 and Log 2-fold changes >1 (+/-). When grouped into metabolism pathways, a wide range of metabolites across 88 different pathways showed significant differences across sites (Suppl. Fig. 5). Most notably, purine and pyramidine metabolism, fatty acid oxidation, and metabolism of several sugar molecules were greater in the primary forest (MGV; Suppl. Fig. 5). Conversely, starch and sucrose metabolism, androgen and estrogen metabolism, and cardiolipin biosynthesis were greater in the secondary forest (TLK; Suppl. Fig. 5).

*Predictors of gut microbiome variation*

Community composition did not vary in response to nutrient intakes overall, though macronutrient intakes were generally positively correlated with microbial abundances (posterior mean_AP_: -0.086, ESS: 1000, pMCMC: 0.542; posterior mean_NDF_: -0.253, ESS: 1000, pMCMC: 0.864; posterior mean_Fat_: -0.893, ESS: 1000, pMCMC: 0.488; but posterior mean_TNC_: 0.068, ESS: 876, pMCMC: 0.934), which explained minimal variance (proportion of variance u_asv:ap_ + u_asv:ndf_ + u_asv:tnc_ + u_asv:fat_: 2.8%, CI 2.5-3.2%).

GLMMs examining macronutrient intakes as predictors of GM community composition revealed that community composition did not vary in response to nutrient intakes overall, though macronutrient intakes were generally positively correlated with microbial abundances (posterior mean_AP_: -0.086, ESS: 1000, pMCMC: 0.542; posterior mean_NDF_: -0.253, ESS: 1000, pMCMC: 0.864; posterior mean_Fat_: -0.893, ESS: 1000, pMCMC: 0.488; but posterior mean_TNC_: 0.068, ESS: 876, pMCMC: 0.934), which explained minimal variance (proportion of variance u_asv:ap_ + u_asv:ndf_ + u_asv:tnc_ + u_asv:fat_: 2.8%, CI 2.5-3.2%). However, estimates from posterior distributions for each random-effect level of specific taxa across each macronutrient intake (AP, NDF, TNC, fat) revealed that NDF, TNC, and fat intakes all drove strong shifts in abundances of specific microbial ASVs, whereas AP intakes did not drive shifts in any ASVs (Suppl. Fig. 6). NDF intakes were positively associated with 5 ASVs, mainly Proteobacteria and Firmicutes, and negatively associated with no ASVs. TNC intakes were positively associated with 17 ASVs, mainly Bacteroidota and Cyanobacteria, and negatively associated with 7 ASVs within the same two phyla in addition to Proteobacteria. Fat intakes were positively associated with 19 ASVs, mostly Bacteroidota, and negatively associated with 5 ASVs from Bacteroidota and Cyanobacteria.

GLMMs examining macronutrient intakes as predictors of PICRUSt2 predicted functional pathway abundances revealed that some functional pathways in the ‘metabolism’ class negatively correlated with associated macronutrient intakes. Available protein (AP) and fat intakes were the best predictors of overall pathway relative abundances (posterior mean_AP_: -27985, ESS: 1000, pMCMC: 0.049; posterior mean_Fat_: -30409, ESS: 1000, pMCMC: 0.028). However, decreases in macronutrient intakes were each associated with enrichment of different functional pathways. Decreased AP intakes were associated with amino acid metabolism pathway enrichment, decreased fat intakes were with lipid metabolism pathway enrichment, and decreased total non-structural carbohydrate (TNC) intakes with carbohydrate metabolism pathway enrichment, meaning macronutrient intakes most strongly influence pathways involved in their own metabolism (Suppl. Fig. 7). Fiber (NDF) did not show such clear relationships, with the decreased NDF intakes being associated with enrichment of xenobiotics degradation and metabolism pathways. Notably, many of these nutrient-by-metabolism pathway effects were weak, especially for NDF and TNC, indicated by effect estimate credible intervals (CIs) spanning zero (Suppl. Fig. 7).

**Supplementary Methods**

*Statistical Analyses*

*Energy and nutrient intakes*: to examine variation in overall energy intakes (kcal) we conducted Welch two-sample t-tests to compare overall calorie intakes across sites, as well as monthly comparisons to identify when differences occur, if any. To examine variation in individual macronutrient intakes we calculated coefficients of variation (CVs) for each macronutrient intake (grams) to examine variation across sites.

*GM alpha and beta diversity*: for alpha diversity we used Wilcoxon rank-sum tests to compare both Shannon Entropy and Faith Phylogenetic Diversity across sites. Wilcoxon tests were chosen due to non-normality of residuals. Then, for beta dissimilarity we used Adonis regressions with 999 permutations using the “vegan” package to compare Bray-Curtis and Jaccard distances matrices across sites (Oksanen et al. 2013).

*GM community composition*: we used a Bayesian framework and built generalized linear mixed effects models (GLMMs) with Poisson errors and log links using the “MCMCglmm” package in R (Hadfield 2016, Sweeny et al. 2023). We removed all global singletons and doubletons in the dataset and removed all taxonomy assignments with a confidence level below 0.9. We used ‘Site’ as the fixed effect, and included ‘ASV ID’, ‘Sample ID’, ‘Individual ID’, and an ‘ASV ID:Site’ interaction term as random effects. We then calculated differential abundances using the posterior distributions for each random-effect level of taxa across sites. We subtracted the posterior distributions for each ASV between group (MGV vs TLK) levels from Poisson model outputs and calculated the mean difference and the highest posterior density interval (HPDI) to estimate differential abundance for each ASV. Finally, we removed any taxon effects for which credible intervals (CIs) did not span zero to increase robusticity.

*Core microbiome*: we identified ASVs with relative abundances of at least 1% in at least 50% of samples using the “microbiome” package with detection set to 0.01 and prevalence to 0.5 (Lahti et al. 2017).

*Functional pathway predictions*: using KEGG functional pathway predictions in the “ggpicrust2” package (Yang et al. 2023), we conducted a differential abundance (DA) analysis using the LinDA method with Benjamini-Hochberg corrections (Zhou et al. 2022). We additionally created a heatmap of the top 30 pathways with greatest differential abundances across sites and examined overall patterns across sites with a PCA comparing MGV and TLK centroids.

*SCFAs and metabolites:* we used Wilcoxon rank-sum tests to compare each SCFA across sites. Wilcoxon tests were chosen due to non-normality of residuals. We normalized metabolite intensity values, then used t-tests with Benjamini-Hochberg corrections to compare metabolite intensities across sites, and plotted results as volcano plots based on corrected p-values and log2 -fold changes using the “EnhancedVolcano” package (Blighe et al. 2024).

*Nutrient intakes and microbial abundances:* we discretized nutrient intake variables (AP, NDF, fat, and TNC) based upon data spread, with “low” intakes corresponding to the lower quadrant of values, “medium” as the middle two quadrants, and “high” as the upper quadrant. We then built GLMMs to examine whether macronutrient intakes predict gut microbial relative abundances. For these models we had microbial ‘count’ as the response variable, macronutrient intakes (AP, NDF, fat, TNC) as fixed effects and ‘Individual ID’, ‘Sample ID’, ‘ASV ID’, and ‘Nutrient Intake:ASV ID’ interaction terms as random effects.

*Nutrient intakes and functional pathways*: using the same discretized values as above, we then built GLMMs to examine whether macronutrient intakes predict macronutrient metabolism pathway relative abundances. For these models we had KEGG metabolism pathway abundances as the response variable, macronutrient intakes (AP, NDF, fat, TNC) as fixed effects and ‘Individual ID’, ‘Sample ID’, ‘KEGG ID’, and ‘Nutrient Intake:KEGG ID’ interaction terms as random effects.

*Energetic outcomes*: we built GLMMs with uCP concentration as the response variable and KEGG metabolism pathway abundances as the fixed effect with ‘Site’, ‘Individual ID’, and a ‘KEGG Pathway Abundance:KEGG ID’ interaction term as random effects. Note that KEGG metabolism pathway abundances were CLR-transformed to fit a normal distribution for modeling. We also built GLMMs with the same structure as above but using normalized metabolite intensities as the fixed effect, with ‘Site’, ‘Individual ID’, ‘Pathway’ and a ‘normalized intensity:Pathway’ interaction term as random effects. In this model, ‘Pathway’ represents the pathways assigned to each metabolite using the SMPDB metabolites library (see *Metabolomics*).

**Methods references not included in main reference list*

Blighe, K., Rana, S., & Lewis, M. (2024). *EnhancedVolcano: Publication-ready volcano plots with enhanced colouring and labeling*. R package version 1.24.0. <https://github.com/kevinblighe/EnhancedVolcano>.

Lahti, L., Shetty, S., et al. (2017). Tools for microbiome analysis in R. Retrieved from: <http://microbiome.github.com/microbiome>.

**Supplementary Figures**

**Supplementary Figure 1** Heat maps representing the core microbiome from ruffed lemurs at primary (MGV) and secondary (TLK) forest sites in Ranomafana National Park, Madagascar, based on detection threshold of 1% and prevalence of 50% i.e., ASVs with relative abundances of at least 1% in at least 50% of samples. Boxes around taxonomy labels indicate ASVs which are shared across the core microbiomes of the two sites, with color representing phylum classification. ASVs which are unclassified at the genus level are given numbers for differentiation and identification e.g., G1, G2.

**Supplementary Figure 2** Balanced subsampling (10,000 iterations) of Shannon and Faith PD alpha diversity between MGV and TLK ruffed lemur populations, controlling for unequal sample sizes.

**Supplementary Figure 3** Characterization of MetaCyc predicted functional pathways highlighting the greatest differential abundances across sites for MetaCyc metabolic pathways (top 30 of 62 shown).

**MetaCyc**

**Supplementary Figure 4** Heatmap showing relative abundances of MetaCyc metabolic pathways with Z-scores based on CLR transformed relative abundances, and PCA showing differences in overall functional pathway abundances across two forest sites (MGV and TLK) in Ranomafana National Park, Madagascar.

**Acetate	Butyrate	Propionate	Isovalerate**

**Supplementary Figure 5** Short-chain fatty acid (SCFA) differences across two forest sites (MGV and TLK) in Ranomafana National Park, Madagascar.

**Supplementary Figure 6** Log 2-fold changes in metabolite intensity grouped by KEGG Pathway across two forest sites (MGV and TLK) in Ranomafana National Park, Madagascar. Positive values represent greater intensities at TLK, and negative values represent lower intensities at TLK, compared to MGV.

N/A

**Supplementary Figure 7** Differential abundances of microbial ASV abundances in response to nutrient intakes from ruffed lemurs in Ranomafana National Park, Madagascar, calculated from GLMMs. Violin plots represent the distribution of effect estimates for each nutrient, with point sizes representing inverse variance of estimates and rectangles indicating microbial ASVs with the greatest (positive or negative) differential abundances.

**Supplementary Figure 8** Differential abundances of KEGG functional pathway predictions (metabolism pathway class) in response to nutrient intakes from ruffed lemurs in Ranomafana National Park, Madagascar, calculated from GLMMs. Violin plots represent the distribution of effect estimates for each nutrient, with point sizes representing inverse variance of estimates and rectangles indicating KEGG IDs with the greatest (positive or negative) differential abundances (AAM: amino acid metabolism, BoSM: biosynthesis of other secondary metabolites, CM: carbohydrate metabolism, EM: energy metabolism, GBM: glycan biosynthesis and metabolism, LM: lipid metabolism, MoCV: metabolism of cofactors and vitamins, MoAA: metabolism of other amino acids, MoTP: metabolism of terpenoids and polyketides, NM: nucleotide metabolism, XDM: xenobiotics degradation and metabolism). Forest plots represent point estimates and HPDIs for the KEGG IDs involved in the 20 strongest increases and decreases of abundance from high to low nutrient intake classes (AP: available protein; NDF: neutral detergent fiber; fat; TNC: total non-structural carbohydrate).

**Supplementary Figure 9** Monthly variation in energy balance (measured by urinary C-peptide) of ruffed lemurs across two sites (MGV and TLK) in Ranomafana National Park, Madagascar. Adapted from (25).

**Supplementary Figure 10** Heatmap showing correlations among PICRUST2 KEGG pathway predictions and measured metabolites pathways across all samples. KEGG pathways are grouped at the KEGG Class level.

**Supplementary Figure 11** Heatmap showing correlations among PICRUST2 KEGG pathway predictions and measured metabolites pathways across all samples. KEGG pathways are grouped at the KEGG Subclass level.

**Supplementary Tables**

**Supplementary Table 1** Monthly t-test comparisons of energy intakes (kcal) across MGV and TLK sites in Ranomafana National Park. Significant values are in bold. Note: there were insufficient data collected in August to calculate monthly averages for nutrient and energy intakes.

| **Month** | **t-statistic** | **Degrees of freedom** | **p-value** |
| --- | --- | --- | --- |
| September | 1.065 | 22.528 | 0.298 |
| October | -0.614 | 17.085 | 0.547 |
| **November** | **2.171** | **25.853** | **0.039** |
| December | 0.054 | 5.460 | 0.959 |
| January | 1.522 | 3.415 | 0.214 |
| February | 1.044 | 2.379 | 0.391 |
| March | -0.077 | 18.856 | 0.939 |
| April | -0.480 | 8.457 | 0.644 |
| May | -0.676 | 15.756 | 0.509 |
| June | 1.250 | 8.238 | 0.246 |
| **July** | **2.441** | **11.692** | **0.032** |

**Supplementary Table 2** Adjusted p-values, log 2-fold changes, t-statistics and upper and lower confidence intervals for t-test metabolite comparisons across MGV and TLK sites in Ranomafana National Park. Only metabolites with significant p-values are included (N=1430).

| **Compound** | **Adjusted p-value** | **log2FC** | **t-statistic** | **Lower CI** | **Upper CI** |
| --- | --- | --- | --- | --- | --- |
| 2-Hexaprenyl-3-methyl-5-hydroxy-6-methoxy-1,4-benzoquinol | 4.53E-03 | 1.16E+01 | 5.32E+00 | 7.09E+00 | 1.61E+01 |
| UDP-N-acetyl-D-galactosaminuronic acid | 1.02E-03 | 1.27E+01 | 5.91E+00 | 8.33E+00 | 1.72E+01 |
| (14alpha,17beta,20S,22R)-14,20-Epoxy-17-hydroxy-1-oxowitha-3,5,24-trienolide | 7.62E-03 | -4.32E+00 | -5.32E+00 | -6.02E+00 | -2.61E+00 |
| PE(14:1(9Z)/16:1(9Z)) Esi-15.4349985 | 4.46E-04 | 7.96E+00 | 5.42E+00 | 5.04E+00 | 1.09E+01 |
| Armillatin | 3.67E-03 | 1.23E+01 | 5.43E+00 | 7.60E+00 | 1.69E+01 |
| 3beta,15alpha-Diacetoxylanosta-8,24-dien-26-oic acid | 2.86E-02 | 3.83E+00 | 4.21E+00 | 1.94E+00 | 5.72E+00 |
| 7alpha-Hydroxy-3-oxo-5beta-cholan-24-oic Acid Esi-16.205996 | 3.92E-02 | -3.49E+00 | -3.95E+00 | -5.33E+00 | -1.64E+00 |
| UDP-N-acetyl-D-galactosaminuronic acid Esi-14.977998 | 7.26E-04 | 1.27E+01 | 6.08E+00 | 8.43E+00 | 1.70E+01 |
| Glucolepidiin | 3.85E-03 | -9.29E+00 | -5.33E+00 | -1.29E+01 | -5.70E+00 |
| (14alpha,17beta,20S,22R)-14,20-Epoxy-17-hydroxy-1-oxowitha-3,5,24-trienolide Esi-14.937006 | 1.96E-02 | -3.74E+00 | -4.54E+00 | -5.47E+00 | -2.02E+00 |
| Capsicum annuum Fluorescent chlorophyll catabolite | 3.25E-02 | 6.08E+00 | 3.91E+00 | 2.90E+00 | 9.25E+00 |
| 3-(3,4-Dihydroxyphenyl)lactate Esi-4.8999987 | 3.63E-02 | -2.45E+00 | -3.77E+00 | -3.77E+00 | -1.12E+00 |
| Chlorogenic acid Esi-4.5650015 | 4.81E-02 | -3.57E+00 | -3.63E+00 | -5.59E+00 | -1.56E+00 |
| Nigakilactone A | 4.24E-02 | 9.64E+00 | 3.70E+00 | 4.30E+00 | 1.50E+01 |
| C37 H68 N6 O4 S Esi-16.362 | 3.61E-02 | 9.92E+00 | 3.99E+00 | 4.74E+00 | 1.51E+01 |
| PE(14:1(9Z)/16:1(9Z)) Esi-15.520997 | 6.04E-04 | 7.29E+00 | 5.30E+00 | 4.55E+00 | 1.00E+01 |
| Veranisatin C | 3.23E-02 | -3.59E+00 | -4.06E+00 | -5.42E+00 | -1.76E+00 |
| 5'-(3',4'-dihydroxyphenyl)-gamma-valerolactone sulfate Esi-7.7869987 | 1.30E-02 | -3.62E+00 | -4.72E+00 | -5.22E+00 | -2.03E+00 |
| C43 H58 O6 | 1.99E-02 | -3.62E+00 | -4.58E+00 | -5.29E+00 | -1.96E+00 |
| Lucidenic acid J | 3.81E-02 | 8.66E+00 | 3.91E+00 | 4.06E+00 | 1.33E+01 |
| PE(20:5(5Z,8Z,11Z,14Z,17Z)/15:0) | 2.31E-02 | 5.52E+00 | 3.79E+00 | 2.62E+00 | 8.42E+00 |
| 7-Hydroxy-(S)-usnate | 1.00E-02 | -3.48E+00 | -4.60E+00 | -5.02E+00 | -1.93E+00 |
| Armillatin Esi-16.617002 | 6.74E-06 | 1.28E+01 | 8.05E+00 | 9.58E+00 | 1.61E+01 |
| Mosesin 4 | 3.35E-02 | -2.94E+00 | -4.11E+00 | -4.44E+00 | -1.45E+00 |
| Cerberoside | 4.81E-02 | 2.61E+00 | 3.65E+00 | 1.15E+00 | 4.08E+00 |
| Hexyl glucoside Esi-6.9909983 | 4.99E-02 | -2.87E+00 | -3.73E+00 | -4.47E+00 | -1.27E+00 |
| Norathyriol Esi-7.070002 | 7.52E-03 | 1.21E+01 | 4.90E+00 | 7.02E+00 | 1.71E+01 |
| Nimodipine | 3.45E-02 | 8.35E+00 | 4.28E+00 | 4.19E+00 | 1.25E+01 |
| C22 H32 O4 | 2.39E-02 | -8.87E+00 | -4.44E+00 | -1.31E+01 | -4.68E+00 |
| Estradiol-17-phenylpropionate | 4.97E-02 | -9.36E+00 | -3.76E+00 | -1.45E+01 | -4.18E+00 |
| 3beta,15alpha-Diacetoxylanosta-8,24-dien-26-oic acid Esi-16.565 | 6.17E-03 | 4.73E+00 | 5.36E+00 | 2.89E+00 | 6.58E+00 |
| C45 H64 O13 S | 4.21E-02 | 8.85E+00 | 4.12E+00 | 4.28E+00 | 1.34E+01 |
| PE(20:2(11Z,14Z)/14:0) | 3.45E-02 | -5.45E+00 | -3.53E+00 | -8.52E+00 | -2.39E+00 |
| Acetic acid | 2.24E-05 | -9.16E-01 | -5.88E+00 | -1.22E+00 | -6.09E-01 |
| C21 H16 N17 | 5.02E-03 | -1.93E+00 | -5.08E+00 | -2.71E+00 | -1.15E+00 |
| PE(20:5(5Z,8Z,11Z,14Z,17Z)/15:0) Esi-16.528 | 1.14E-02 | 5.98E+00 | 4.11E+00 | 3.08E+00 | 8.87E+00 |
| C21 H26 O5 | 2.37E-02 | 8.98E+00 | 4.37E+00 | 4.70E+00 | 1.33E+01 |
| (3b,6b,8b,12a)-8,12-Epoxy-7(11)-eremophilene-6-angeloyloxy-8,12-dimethoxy-3-ol | 3.92E-02 | 8.94E+00 | 3.88E+00 | 4.16E+00 | 1.37E+01 |
| Notoginsenoside R1 Esi-14.550002 | 3.63E-02 | 9.04E+00 | 3.98E+00 | 4.30E+00 | 1.38E+01 |
| Loganin pentaacetate Esi-6.6260033 | 4.84E-02 | -2.88E+00 | -3.61E+00 | -4.51E+00 | -1.25E+00 |
| Caffeic acid 3-sulfate | 3.17E-03 | -4.36E+00 | -5.23E+00 | -6.06E+00 | -2.66E+00 |
| 5-Hydroxy-7,3',4'-trimethoxy-8-methylisoflavone 5-neohesperidoside | 4.66E-02 | -2.35E+00 | -3.64E+00 | -3.67E+00 | -1.03E+00 |
| Phloroacetophenone 6'-[xylosyl-(1-6)-glucoside] | 7.71E-03 | -3.14E+00 | -4.80E+00 | -4.48E+00 | -1.80E+00 |
| LysoPE(18:3(9Z,12Z,15Z)/0:0) | 4.25E-02 | 1.93E+00 | 3.71E+00 | 8.64E-01 | 3.00E+00 |
| Caffeic acid 3-sulfate Esi-4.488 | 1.36E-02 | -9.86E+00 | -4.90E+00 | -1.41E+01 | -5.63E+00 |
| C32 H44 O20 | 1.66E-02 | -3.74E+00 | -4.38E+00 | -5.50E+00 | -1.98E+00 |
| Cinnamtannin A1 | 4.76E-02 | -2.36E+00 | -3.62E+00 | -3.69E+00 | -1.03E+00 |
| C7 H14 O6 | 1.61E-02 | -2.23E+00 | -4.37E+00 | -3.27E+00 | -1.18E+00 |
| Ethyl 7-epi-12-hydroxyjasmonate glucoside | 2.81E-02 | -1.75E+00 | -4.01E+00 | -2.64E+00 | -8.55E-01 |
| Cinchonain Ib Esi-7.882998 | 1.47E-02 | -2.45E+00 | -4.52E+00 | -3.57E+00 | -1.33E+00 |
| 1-O-Sinapoylglucose | 5.81E-03 | -2.24E+00 | -5.03E+00 | -3.15E+00 | -1.33E+00 |
| C48 H84 N12 O | 1.59E-02 | 2.05E+00 | 4.42E+00 | 1.09E+00 | 3.00E+00 |
| (5b,7a,12a)-2-(3-methoxyphenyl)-2-oxoethyl ester-7,12-dihydroxy-cholan-24-oic acid | 3.34E-02 | -1.78E+00 | -3.90E+00 | -2.71E+00 | -8.45E-01 |
| NNAL-N-glucuronide | 3.89E-02 | 5.00E+00 | 3.72E+00 | 2.26E+00 | 7.74E+00 |
| Isofucosterol 3-O-[6-O-(9-Octadecenoyl)-b-D-glucopyranoside] Esi-15.1449995 | 6.41E-03 | 2.39E+00 | 4.89E+00 | 1.39E+00 | 3.39E+00 |
| Garcimangosone D | 6.70E-03 | -2.52E+00 | -4.86E+00 | -3.57E+00 | -1.46E+00 |
| Primary fluorescent chlorophyll catabolite Esi-16.510002 | 7.52E-03 | 7.05E+00 | 4.78E+00 | 4.04E+00 | 1.01E+01 |
| Acuminoside | 3.35E-02 | -2.32E+00 | -3.89E+00 | -3.55E+00 | -1.10E+00 |
| 3,7-Dimethyl-5-octene-1,7-diol 1-glucoside Esi-9.513 | 4.47E-02 | -2.73E+00 | -3.67E+00 | -4.26E+00 | -1.21E+00 |
| Agrocin 84 | 6.70E-03 | -1.19E+01 | -4.87E+00 | -1.70E+01 | -6.92E+00 |
| 1,3,6-Tri-O-galloylglucose | 4.95E-02 | 3.11E+00 | 3.66E+00 | 1.36E+00 | 4.86E+00 |
| Shanzhiside Esi-4.7519994 | 1.88E-02 | -2.14E+00 | -4.21E+00 | -3.17E+00 | -1.10E+00 |
| D-Altronate Esi-0.8169998 | 2.59E-02 | -2.30E+00 | -4.14E+00 | -3.44E+00 | -1.16E+00 |
| C15 H16 N5 O6 | 1.45E-02 | -1.68E+00 | -4.42E+00 | -2.46E+00 | -9.02E-01 |
| 2-Phenylaminoadenosine | 4.99E-02 | -2.05E+00 | -3.59E+00 | -3.22E+00 | -8.86E-01 |
| C39 H80 N19 O2 | 2.76E-03 | 3.23E+00 | 5.77E+00 | 2.07E+00 | 4.39E+00 |
| Estradiol-17-phenylpropionate Esi-14.981997 | 8.79E-03 | -6.49E+00 | -4.70E+00 | -9.31E+00 | -3.67E+00 |
| Leucomycin V Esi-14.941997 | 2.71E-02 | -2.42E+00 | -4.09E+00 | -3.64E+00 | -1.21E+00 |
| C26 H36 N4 O | 2.56E-02 | 9.38E+00 | 4.08E+00 | 4.67E+00 | 1.41E+01 |
| Leukotriene C4 | 4.41E-02 | 8.58E+00 | 3.66E+00 | 3.79E+00 | 1.34E+01 |
| 4-Sulfobenzyl alcohol | 3.35E-02 | -2.26E+00 | -3.89E+00 | -3.45E+00 | -1.07E+00 |
| Isofucosterol 3-O-[6-O-(9-Octadecenoyl)-b-D-glucopyranoside] Esi-15.010004 | 6.70E-03 | 2.75E+00 | 4.84E+00 | 1.59E+00 | 3.90E+00 |
| C23 H44 N23 O | 3.52E-02 | 6.26E+00 | 3.79E+00 | 2.89E+00 | 9.63E+00 |
| Gentamicin A Esi-12.769 | 1.59E-02 | 9.53E+00 | 4.46E+00 | 5.13E+00 | 1.39E+01 |
| Austalide I | 3.93E-02 | 8.89E+00 | 3.75E+00 | 4.03E+00 | 1.37E+01 |
| Mitoxantrone Esi-5.710999 | 1.52E-02 | -2.59E+00 | -4.37E+00 | -3.80E+00 | -1.38E+00 |
| N-Hydroxy-L-tyrosine | 1.40E-02 | -1.94E+00 | -4.42E+00 | -2.84E+00 | -1.04E+00 |
| Eriodictyol 7-(6-trans-p-coumaroylglucoside) Esi-4.809001 | 3.14E-02 | -2.50E+00 | -3.95E+00 | -3.79E+00 | -1.20E+00 |
| Veranisatin C Esi-3.876001 | 1.60E-02 | -3.48E+00 | -4.42E+00 | -5.10E+00 | -1.86E+00 |
| (7Z,10Z,13Z,16Z,19Z)-Docosapentaenoyl-CoA | 2.49E-02 | -6.62E+00 | -3.81E+00 | -1.01E+01 | -3.15E+00 |
| Ethyl 7-epi-12-hydroxyjasmonate glucoside Esi-6.869998 | 1.10E-02 | -2.46E+00 | -4.54E+00 | -3.57E+00 | -1.35E+00 |
| 2,2-Dibutyl-3-(4-methoxyphenyl)-4-methyl-2H-1-benzopyran-7-ol acetate | 7.57E-03 | -3.71E+00 | -5.11E+00 | -5.22E+00 | -2.20E+00 |
| 4-Methylumbelliferyl acetate | 2.87E-02 | -2.75E+00 | -4.00E+00 | -4.15E+00 | -1.34E+00 |
| 3-Methoxy-4-hydroxyphenylglycol sulfate Esi-5.0460014 | 6.79E-03 | -2.57E+00 | -5.05E+00 | -3.62E+00 | -1.52E+00 |
| Cinnamtannin A1 Esi-4.6269994 | 2.12E-02 | -2.79E+00 | -4.18E+00 | -4.15E+00 | -1.42E+00 |
| Hordatine A Esi-14.480995 | 3.62E-02 | 9.26E+00 | 4.16E+00 | 4.55E+00 | 1.40E+01 |
| C21 H42 N2 O2 S3 | 4.81E-02 | -8.35E+00 | -3.61E+00 | -1.31E+01 | -3.63E+00 |
| 3-Deoxy-D-manno-octulosonate Esi-1.2109997 | 3.44E-02 | -1.38E+00 | -3.82E+00 | -2.11E+00 | -6.40E-01 |
| Decuroside III | 1.75E-02 | -1.94E+00 | -4.41E+00 | -2.85E+00 | -1.03E+00 |
| Coenzyme Q10 Esi-15.712002 | 1.08E-03 | 6.57E+00 | 5.32E+00 | 4.09E+00 | 9.05E+00 |
| Kelampayoside A | 4.29E-03 | -1.85E+00 | -5.09E+00 | -2.59E+00 | -1.11E+00 |
| PE(14:1(9Z)/16:1(9Z)) Esi-14.843997 | 1.95E-02 | 6.77E+00 | 3.83E+00 | 3.27E+00 | 1.03E+01 |
| Malonylgenistin Esi-6.084 | 4.71E-02 | -1.82E+00 | -3.76E+00 | -2.83E+00 | -8.19E-01 |
| Isofucosterol 3-O-[6-O-(9,12-Octadecadienoyl)-b-D-glucopyranoside] Esi-14.960001 | 1.37E-02 | 2.83E+00 | 4.54E+00 | 1.55E+00 | 4.11E+00 |
| (-)-Matairesinol 4'-[apiosyl-(1-2)-glucoside] | 1.59E-02 | -9.44E+00 | -4.58E+00 | -1.37E+01 | -5.16E+00 |
| N-tert-Butyloxycarbonyl-deacetyl-leupeptin | 1.94E-02 | -7.31E+00 | -4.19E+00 | -1.09E+01 | -3.75E+00 |
| > limit Esi-14.386995 | 2.65E-02 | 6.09E+00 | 4.06E+00 | 3.02E+00 | 9.16E+00 |
| 1-O-Galloylglycerol | 3.40E-02 | -1.84E+00 | -3.82E+00 | -2.82E+00 | -8.56E-01 |
| 1-O-2'-Hydroxy-4'-methoxycinnamoyl-b-D-glucose Esi-5.2870016 | 1.39E-02 | -2.07E+00 | -4.47E+00 | -3.03E+00 | -1.12E+00 |
| Anhwiedelphinine | 3.40E-02 | 5.84E+00 | 3.88E+00 | 2.75E+00 | 8.94E+00 |
| C25 H28 N4 O2 | 1.50E-02 | -2.31E+00 | -4.38E+00 | -3.38E+00 | -1.23E+00 |
| Cortolone-3-glucuronide | 1.42E-02 | -3.09E+00 | -4.40E+00 | -4.53E+00 | -1.66E+00 |
| (8Z,11Z,14Z,17Z)-Icosatetraenoyl-CoA | 4.05E-02 | 2.51E+00 | 3.64E+00 | 1.11E+00 | 3.91E+00 |
| 12-O-Palmitoyl-16-hydroxyphorbol 13-acetate Esi-14.951998 | 3.45E-02 | 9.12E+00 | 3.90E+00 | 4.30E+00 | 1.39E+01 |
| C25 H16 N4 O5 | 4.29E-02 | -9.04E+00 | -3.74E+00 | -1.40E+01 | -4.07E+00 |
| Thesinine 4'-O-glucoside Esi-5.296998 | 2.33E-02 | -2.26E+00 | -4.11E+00 | -3.38E+00 | -1.13E+00 |
| 12alpha-Fluoro-11beta,17beta-dihydroxyandrosta-1,4-dien-3-one Esi-11.895002 | 3.18E-02 | 9.78E+00 | 4.19E+00 | 4.90E+00 | 1.47E+01 |
| Glucovanillin | 3.34E-02 | -1.24E+00 | -3.87E+00 | -1.90E+00 | -5.85E-01 |
| Avenanthramide 2s | 3.34E-02 | -8.42E+00 | -3.87E+00 | -1.29E+01 | -3.97E+00 |
| Carfentrazone | 3.79E-02 | -8.80E+00 | -3.76E+00 | -1.36E+01 | -4.01E+00 |
| 1-(3-Methyl-2-butenoyl)-6-apiosylglucose | 8.92E-03 | -1.11E+01 | -4.67E+00 | -1.60E+01 | -6.26E+00 |
| NNAL-N-glucuronide Esi-12.372993 | 3.30E-02 | 4.70E+00 | 3.91E+00 | 2.24E+00 | 7.16E+00 |
| Eriocitrin | 9.78E-05 | -2.44E+00 | -6.89E+00 | -3.16E+00 | -1.71E+00 |
| Acronycidine | 9.57E-03 | 1.06E+01 | 4.63E+00 | 5.93E+00 | 1.53E+01 |
| C23 H36 O6 | 3.47E-02 | 9.04E+00 | 3.90E+00 | 4.26E+00 | 1.38E+01 |
| C32 H44 O20 Esi-4.475001 | 2.50E-02 | -3.34E+00 | -4.17E+00 | -4.99E+00 | -1.69E+00 |
| C12 H28 N13 O3 S | 3.33E-02 | -2.23E+00 | -3.93E+00 | -3.39E+00 | -1.07E+00 |
| Sambacin Esi-5.328002 | 1.18E-02 | -1.73E+00 | -4.58E+00 | -2.50E+00 | -9.54E-01 |
| TG(18:3(9Z,12Z,15Z)/14:0/22:6(4Z,7Z,10Z,13Z,16Z,19Z))[iso6] Esi-16.042994 | 3.47E-02 | 1.84E+00 | 3.80E+00 | 8.49E-01 | 2.82E+00 |
| Nb-trans-Feruloylserotonin glucoside Esi-6.9559975 | 1.07E-02 | -1.96E+00 | -4.65E+00 | -2.83E+00 | -1.10E+00 |
| C27 H28 N7 Esi-13.8670025 | 3.07E-02 | -8.77E+00 | -4.00E+00 | -1.33E+01 | -4.27E+00 |
| dTDP / Thymidine 5'-diphosphate | 1.88E-03 | -3.67E+00 | -5.72E+00 | -4.99E+00 | -2.35E+00 |
| C39 H80 N19 O2 Esi-14.752996 | 6.46E-04 | 3.35E+00 | 6.21E+00 | 2.24E+00 | 4.45E+00 |
| C23 H34 O16 Esi-2.945 | 8.71E-04 | -3.83E+00 | -5.88E+00 | -5.15E+00 | -2.50E+00 |
| Spirotaccagenin Esi-14.717003 | 1.07E-02 | 1.02E+01 | 5.17E+00 | 6.04E+00 | 1.44E+01 |
| Glycoperine | 4.46E-04 | -1.21E+01 | -6.22E+00 | -1.61E+01 | -8.15E+00 |
| 4-Sulfobenzyl alcohol Esi-6.091002 | 7.11E-03 | -6.60E+00 | -5.15E+00 | -9.26E+00 | -3.93E+00 |
| Synaptolepis factor K1 | 4.71E-02 | 8.40E+00 | 3.66E+00 | 3.69E+00 | 1.31E+01 |
| Cucurbitacin S | 1.24E-02 | 1.01E+01 | 4.50E+00 | 5.51E+00 | 1.47E+01 |
| PE(20:4(8Z,11Z,14Z,17Z)/15:0) Esi-16.705997 | 1.24E-02 | 5.90E+00 | 4.09E+00 | 3.03E+00 | 8.76E+00 |
| Arctiopicrin Esi-11.731998 | 1.60E-02 | 9.64E+00 | 4.32E+00 | 5.09E+00 | 1.42E+01 |
| 2E-hexadecenoyl-CoA | 1.63E-03 | 4.47E+00 | 4.92E+00 | 2.66E+00 | 6.27E+00 |
| Cinnamtannin A1 Esi-4.6410017 | 1.93E-02 | -2.51E+00 | -4.21E+00 | -3.73E+00 | -1.29E+00 |
| 7-Hydroxy-(S)-usnate Esi-14.765998 | 2.77E-02 | -4.02E+00 | -4.03E+00 | -6.05E+00 | -1.98E+00 |
| Lamioside Esi-9.036002 | 3.10E-02 | 4.49E+00 | 3.98E+00 | 2.18E+00 | 6.81E+00 |
| Agrocin 84 Esi-4.565 | 5.88E-03 | -1.19E+01 | -4.93E+00 | -1.68E+01 | -6.96E+00 |
| Tetraneurin E | 3.93E-02 | 8.55E+00 | 3.73E+00 | 3.87E+00 | 1.32E+01 |
| Calophyllolide | 1.91E-02 | -2.24E+00 | -4.20E+00 | -3.33E+00 | -1.15E+00 |
| N-Acetyl-L-phenylalanine | 3.23E-02 | -1.52E+00 | -4.10E+00 | -2.29E+00 | -7.50E-01 |
| C16 H16 N7 O3 | 3.26E-02 | -1.62E+00 | -3.91E+00 | -2.47E+00 | -7.72E-01 |
| Coenzyme Q10 Esi-14.328001 | 1.06E-11 | 2.51E+00 | 9.63E+00 | 1.99E+00 | 3.03E+00 |
| Ethyl 7-epi-12-hydroxyjasmonate glucoside Esi-7.220998 | 3.65E-02 | -1.87E+00 | -3.77E+00 | -2.88E+00 | -8.56E-01 |
| (+)-Lyoniresinol 9-glucoside | 9.66E-03 | -3.39E+00 | -4.72E+00 | -4.86E+00 | -1.91E+00 |
| 3alpha-Acetomethoxy-11alpha-oxo-12-ursen-24-oic acid | 3.88E-02 | 8.56E+00 | 3.86E+00 | 3.97E+00 | 1.32E+01 |
| Neoarctin B | 2.21E-02 | -4.57E+00 | -4.22E+00 | -6.81E+00 | -2.34E+00 |
| 17,21-Epoxy-9-fluoro-11beta-hydroxypregn-4-ene-3,20-dione Esi-4.8450007 | 1.84E-02 | -9.59E+00 | -4.39E+00 | -1.41E+01 | -5.07E+00 |
| beta-Zearalanol Esi-12.539998 | 1.10E-02 | 1.03E+01 | 4.56E+00 | 5.66E+00 | 1.49E+01 |
| C25 H44 N25 O3 | 3.19E-02 | 1.40E+00 | 3.92E+00 | 6.69E-01 | 2.12E+00 |
| Tyrosyl-Asparagine | 3.35E-02 | -9.21E+00 | -4.06E+00 | -1.39E+01 | -4.48E+00 |
| Hydroxyvernolide | 8.51E-04 | -1.06E+01 | -6.10E+00 | -1.42E+01 | -7.04E+00 |
| > limit Esi-14.326997 | 9.71E-03 | 6.62E+00 | 4.61E+00 | 3.69E+00 | 9.55E+00 |
| RU-0211 | 1.28E-02 | 9.28E+00 | 4.47E+00 | 5.04E+00 | 1.35E+01 |
| Ethyl Oxalacetate | 4.45E-02 | -8.95E+00 | -3.66E+00 | -1.40E+01 | -3.95E+00 |
| PE(22:6(4Z,7Z,10Z,13Z,16Z,19Z)/20:5(5Z,8Z,11Z,14Z,17Z)) | 5.50E-04 | -7.00E+00 | -5.22E+00 | -9.67E+00 | -4.34E+00 |
| Agrocin 84 Esi-4.5680003 | 3.12E-03 | -1.22E+01 | -5.36E+00 | -1.69E+01 | -7.52E+00 |
| 1-Pentadecanecarboxylic acid Esi-5.296998 | 9.01E-03 | -2.41E+00 | -4.68E+00 | -3.46E+00 | -1.36E+00 |
| Hyperforin Esi-16.647999 | 3.34E-02 | 9.05E+00 | 3.97E+00 | 4.36E+00 | 1.37E+01 |
| 3,4,5-Trimethoxyphenyl glucoside | 1.51E-02 | -2.02E+00 | -4.53E+00 | -2.94E+00 | -1.10E+00 |
| Methylsuccinic acid Esi-1.0439999 | 1.56E-02 | -1.58E+00 | -4.34E+00 | -2.33E+00 | -8.38E-01 |
| C19 H28 Cl N3 O S | 4.48E-02 | -7.91E+00 | -3.98E+00 | -1.21E+01 | -3.71E+00 |
| Mitoxantrone Esi-6.522002 | 3.33E-02 | -1.58E+00 | -3.91E+00 | -2.41E+00 | -7.55E-01 |
| Diosbulbinoside F Esi-7.0980024 | 3.18E-02 | -2.26E+00 | -4.20E+00 | -3.39E+00 | -1.14E+00 |
| C26 H52 O10 | 3.48E-02 | -2.29E+00 | -3.90E+00 | -3.50E+00 | -1.08E+00 |
| Kuwanon J | 4.44E-02 | -2.39E+00 | -3.67E+00 | -3.72E+00 | -1.06E+00 |
| C15 H43 N11 O S3 | 4.75E-02 | 8.42E+00 | 3.63E+00 | 3.68E+00 | 1.32E+01 |
| (+)-Lyoniresinol 9-glucoside Esi-5.9980006 | 8.05E-03 | -3.40E+00 | -4.71E+00 | -4.88E+00 | -1.93E+00 |
| Cyflufenamid Esi-6.238999 | 9.47E-03 | -2.28E+00 | -4.70E+00 | -3.28E+00 | -1.29E+00 |
| Cinncassiol C3 | 3.34E-02 | 8.33E+00 | 3.90E+00 | 3.95E+00 | 1.27E+01 |
| Sambacin Esi-6.261001 | 3.04E-02 | -1.58E+00 | -3.96E+00 | -2.39E+00 | -7.64E-01 |
| Mometasone | 3.65E-02 | -1.34E+00 | -3.76E+00 | -2.07E+00 | -6.13E-01 |
| C22 H32 N7 O2 Esi-14.235995 | 1.51E-02 | 1.05E+01 | 4.36E+00 | 5.56E+00 | 1.54E+01 |
| Chlorobenzilate Esi-4.164001 | 3.60E-02 | -2.21E+00 | -3.80E+00 | -3.40E+00 | -1.02E+00 |
| Acuminoside Esi-8.246998 | 3.35E-02 | -8.32E+00 | -4.19E+00 | -1.25E+01 | -4.13E+00 |
| 3-Vinylbacteriochlorophyllide d | 2.28E-02 | -8.77E+00 | -4.14E+00 | -1.31E+01 | -4.44E+00 |
| Chlorobenzilate Esi-5.0230007 | 3.38E-02 | -8.56E+00 | -3.95E+00 | -1.30E+01 | -4.08E+00 |
| Naphtho[2,3-e]pyrene | 1.10E-02 | -1.80E+00 | -4.58E+00 | -2.60E+00 | -9.94E-01 |
| 16-Feruloyloxypalmitate Esi-12.957 | 3.32E-03 | -1.15E+01 | -5.33E+00 | -1.59E+01 | -7.05E+00 |
| Quassimarin | 4.44E-03 | -7.13E+00 | -5.14E+00 | -9.97E+00 | -4.28E+00 |
| Diclofop methyl | 1.10E-02 | -9.79E+00 | -4.70E+00 | -1.41E+01 | -5.49E+00 |
| 3''-Adenylylstreptomycin Esi-6.4909983 | 3.94E-02 | -2.81E+00 | -3.85E+00 | -4.31E+00 | -1.30E+00 |
| C14 H30 N11 O5 S2 | 3.39E-02 | 9.03E+00 | 4.07E+00 | 4.40E+00 | 1.37E+01 |
| Cassiaside B2 | 1.10E-02 | -5.37E+00 | -4.64E+00 | -7.75E+00 | -2.99E+00 |
| Fexofenadine | 2.44E-02 | 8.65E+00 | 4.54E+00 | 4.62E+00 | 1.27E+01 |
| Proansamitocin | 1.65E-02 | 9.65E+00 | 4.51E+00 | 5.21E+00 | 1.41E+01 |
| beta-Zearalanol Esi-11.729998 | 2.29E-02 | 9.53E+00 | 4.13E+00 | 4.80E+00 | 1.42E+01 |
| 6,8a-Seco-6,8a-deoxy-5-oxoavermectin ''1a'' aglycone | 3.45E-02 | 4.70E+00 | 3.82E+00 | 2.18E+00 | 7.21E+00 |
| C20 H31 N7 O4 | 3.14E-02 | -9.56E+00 | -4.21E+00 | -1.43E+01 | -4.81E+00 |
| Spirotaccagenin Esi-15.837004 | 2.84E-02 | 9.16E+00 | 4.12E+00 | 4.57E+00 | 1.37E+01 |
| 5'-(3',4'-dihydroxyphenyl)-gamma-valerolactone sulfate Esi-7.7850027 | 1.40E-02 | -3.19E+00 | -4.62E+00 | -4.61E+00 | -1.76E+00 |
| Vanillic acid 4-sulfate | 4.23E-02 | -8.94E+00 | -3.72E+00 | -1.39E+01 | -4.01E+00 |
| C19 H28 O12 | 1.24E-04 | -1.23E+01 | -6.88E+00 | -1.59E+01 | -8.62E+00 |
| Cyclotricuspidoside C | 4.74E-02 | 7.97E+00 | 3.62E+00 | 3.47E+00 | 1.25E+01 |
| TG(20:4(5Z,8Z,11Z,14Z)/14:0/18:3(9Z,12Z,15Z))[iso6] Esi-15.021998 | 4.89E-02 | 2.09E+00 | 3.62E+00 | 9.10E-01 | 3.27E+00 |
| Gravacridonediol glucoside Esi-7.3489995 | 3.28E-02 | -8.48E+00 | -3.90E+00 | -1.29E+01 | -4.04E+00 |
| Mosesin 4 Esi-16.183998 | 1.76E-02 | -9.43E+00 | -4.31E+00 | -1.39E+01 | -4.94E+00 |
| Streptothricin F | 2.80E-02 | -3.34E+00 | -4.12E+00 | -5.02E+00 | -1.67E+00 |
| L-2-(Hydroxymethyl)-1,2,3,4-butanetetrol Esi-1.146 | 1.66E-02 | -1.52E+00 | -4.29E+00 | -2.24E+00 | -7.94E-01 |
| PE(15:0/18:0) | 1.87E-02 | -1.62E+00 | -3.85E+00 | -2.45E+00 | -7.83E-01 |
| dTDP / Thymidine 5'-diphosphate Esi-6.188998 | 3.85E-03 | -2.90E+00 | -5.62E+00 | -3.98E+00 | -1.83E+00 |
| N-Acetyl-leu-leu-tyr | 1.65E-02 | 5.13E+00 | 4.37E+00 | 2.72E+00 | 7.54E+00 |
| Catechin 3',7-diglucoside Esi-4.7290025 | 1.34E-02 | -1.78E+00 | -4.47E+00 | -2.60E+00 | -9.66E-01 |
| Oxadixyl | 2.09E-02 | -1.43E+00 | -4.15E+00 | -2.14E+00 | -7.29E-01 |
| 3alpha,12alpha-Dihydroxy-5beta-pregnan-20-one diacetate Esi-15.709998 | 3.37E-02 | 8.57E+00 | 3.95E+00 | 4.09E+00 | 1.30E+01 |
| LysoPE(0:0/22:2(13Z,16Z)) Esi-12.850999 | 3.34E-02 | 8.59E+00 | 3.96E+00 | 4.12E+00 | 1.31E+01 |
| Diclocymet | 3.50E-02 | -8.99E+00 | -4.22E+00 | -1.35E+01 | -4.47E+00 |
| C20 H32 N7 S2 | 7.52E-03 | -2.47E+00 | -4.78E+00 | -3.52E+00 | -1.41E+00 |
| Fucoxanthin Esi-16.198 | 3.12E-02 | -2.29E+00 | -4.17E+00 | -3.44E+00 | -1.15E+00 |
| C13 H24 Cl N2 O7 | 4.99E-03 | -2.76E+00 | -5.49E+00 | -3.80E+00 | -1.71E+00 |
| C25 H32 N3 O13 | 4.00E-02 | -8.13E+00 | -3.71E+00 | -1.26E+01 | -3.65E+00 |
| Acronycidine Esi-9.027001 | 9.67E-03 | 1.06E+01 | 4.63E+00 | 5.92E+00 | 1.53E+01 |
| XMP | 1.52E-02 | -6.65E+00 | -4.07E+00 | -9.92E+00 | -3.38E+00 |
| Ustiloxin D Esi-5.4150004 | 2.76E-03 | -1.05E+01 | -5.50E+00 | -1.44E+01 | -6.59E+00 |
| Glaucarubol 15-O-beta-D-glucopyranoside Esi-5.3809977 | 2.99E-02 | -1.66E+00 | -3.97E+00 | -2.51E+00 | -8.04E-01 |
| Sambacin Esi-5.333003 | 3.27E-02 | -1.51E+00 | -3.91E+00 | -2.30E+00 | -7.22E-01 |
| Isopropyl apiosylglucoside | 3.34E-02 | -3.63E+00 | -3.88E+00 | -5.54E+00 | -1.72E+00 |
| C13 H2 N4 O5 S | 2.89E-02 | -8.40E+00 | -4.44E+00 | -1.24E+01 | -4.40E+00 |
| PE(20:4(8Z,11Z,14Z,17Z)/15:0) Esi-16.98 | 3.12E-03 | 1.94E+00 | 4.63E+00 | 1.11E+00 | 2.77E+00 |
| Ssioriside | 3.15E-02 | -9.73E+00 | -3.96E+00 | -1.48E+01 | -4.70E+00 |
| Gibberellin A97 Esi-12.615002 | 4.62E-04 | 1.18E+01 | 6.24E+00 | 7.94E+00 | 1.57E+01 |
| UDP-N-acetyl-D-galactosaminuronic acid Esi-14.954001 | 1.03E-03 | 7.07E+00 | 6.07E+00 | 4.67E+00 | 9.47E+00 |
| C13 H14 N7 O2 | 2.06E-02 | -1.48E+00 | -4.20E+00 | -2.21E+00 | -7.60E-01 |
| Malonylgenistin Esi-6.0999994 | 6.21E-03 | -1.00E+01 | -4.90E+00 | -1.42E+01 | -5.85E+00 |
| PE(20:5(5Z,8Z,11Z,14Z,17Z)/22:6(4Z,7Z,10Z,13Z,16Z,19Z)) | 2.01E-03 | 6.56E+00 | 4.81E+00 | 3.86E+00 | 9.27E+00 |
| Vanillic acid 4-sulfate Esi-3.2680004 | 4.61E-02 | -8.77E+00 | -3.67E+00 | -1.37E+01 | -3.87E+00 |
| Diphenylcarbazide | 3.39E-02 | 2.66E+00 | 3.84E+00 | 1.24E+00 | 4.08E+00 |
| Ononin Esi-2.3789997 | 4.05E-05 | -3.14E+00 | -7.40E+00 | -4.01E+00 | -2.27E+00 |
| (4S,8R)-8,9-Dihydroxy-p-menth-1(6)-en-2-one | 3.39E-02 | -8.85E+00 | -3.94E+00 | -1.35E+01 | -4.21E+00 |
| C33 H46 N10 O2 | 3.92E-02 | 2.84E+00 | 3.75E+00 | 1.29E+00 | 4.39E+00 |
| PE(14:0/20:5(5Z,8Z,11Z,14Z,17Z)) Esi-13.740998 | 1.24E-02 | 3.36E+00 | 4.11E+00 | 1.73E+00 | 4.98E+00 |
| Lippioside II | 3.25E-02 | -8.55E+00 | -4.03E+00 | -1.29E+01 | -4.17E+00 |
| DMG-MINO Esi-8.822999 | 3.84E-02 | -8.71E+00 | -3.74E+00 | -1.35E+01 | -3.95E+00 |
| C13 H18 Cl2 N3 O2 | 1.48E-03 | -1.08E+01 | -5.86E+00 | -1.45E+01 | -6.99E+00 |
| 4-Sulfobenzyl alcohol Esi-6.016999 | 8.12E-04 | -4.59E+00 | -5.93E+00 | -6.17E+00 | -3.01E+00 |
| Ipolamiide Esi-4.7129984 | 2.20E-02 | -2.47E+00 | -4.26E+00 | -3.66E+00 | -1.27E+00 |
| Pisumic acid Esi-8.412997 :1 | 3.14E-02 | -2.66E+00 | -3.94E+00 | -4.04E+00 | -1.28E+00 |
| (14alpha,17beta,20S,22R)-14,20-Epoxy-17-hydroxy-1-oxowitha-3,5,24-trienolide Esi-14.790002 | 3.32E-02 | -6.89E+00 | -4.01E+00 | -1.04E+01 | -3.34E+00 |
| PE(20:4(8Z,11Z,14Z,17Z)/15:0) Esi-16.508995 | 3.31E-03 | 1.92E+00 | 4.71E+00 | 1.10E+00 | 2.73E+00 |
| Chalcomoracin | 3.20E-02 | -9.01E+00 | -4.01E+00 | -1.36E+01 | -4.38E+00 |
| C9 H14 O4 | 2.76E-03 | -1.17E+01 | -5.40E+00 | -1.61E+01 | -7.25E+00 |
| C19 H28 Cl N3 O S Esi-3.5100002 | 4.44E-02 | -7.92E+00 | -3.99E+00 | -1.21E+01 | -3.73E+00 |
| L-Arabinose Esi-1.0400006 | 4.62E-04 | -1.84E+00 | -6.18E+00 | -2.45E+00 | -1.23E+00 |
| 7-Hydroxy-(S)-usnate Esi-14.523993 | 1.10E-02 | -3.25E+00 | -4.53E+00 | -4.71E+00 | -1.78E+00 |
| C22 H22 N17 O2 | 3.90E-02 | -1.31E+00 | -3.75E+00 | -2.03E+00 | -5.97E-01 |
| DHAP(18:0e) | 3.43E-02 | -8.47E+00 | -4.01E+00 | -1.29E+01 | -4.08E+00 |
| PG(18:3(9Z,12Z,15Z)/18:3(9Z,12Z,15Z)) | 3.01E-02 | 9.38E+00 | 4.08E+00 | 4.64E+00 | 1.41E+01 |
| Hesperetin 3',7-O-diglucuronide Esi-7.040999 | 1.30E-02 | -2.02E+00 | -4.62E+00 | -2.92E+00 | -1.12E+00 |
| 1-(3,4-Dimethoxyphenyl)-1,2-ethanediol 1-O-b-D-glucoside | 4.12E-02 | -8.47E+00 | -3.73E+00 | -1.31E+01 | -3.82E+00 |
| Galactopinitol B | 3.34E-02 | -3.80E+00 | -4.17E+00 | -5.71E+00 | -1.89E+00 |
| 4-Sulfobenzyl alcohol Esi-3.056 | 3.34E-02 | -2.40E+00 | -3.87E+00 | -3.66E+00 | -1.13E+00 |
| Tetrahydrofolic acid | 1.98E-07 | -9.45E-01 | -6.80E+00 | -1.22E+00 | -6.71E-01 |
| dTDP-D-mycaminose Esi-5.485999 | 4.16E-02 | -8.15E+00 | -3.80E+00 | -1.26E+01 | -3.72E+00 |
| Paeonolide Esi-4.960999 | 3.35E-02 | -1.28E+00 | -3.98E+00 | -1.95E+00 | -6.17E-01 |
| Hypercalin B Esi-15.832 | 3.35E-02 | 8.69E+00 | 3.91E+00 | 4.13E+00 | 1.33E+01 |
| C15 H17 Cl O4 | 4.84E-02 | -1.94E+00 | -3.61E+00 | -3.03E+00 | -8.42E-01 |
| Blumenol C O-[rhamnosyl-(1-6)-glucoside] Esi-5.2810016 :1 | 2.72E-02 | -1.97E+00 | -4.10E+00 | -2.96E+00 | -9.81E-01 |
| Ligustroside | 3.38E-02 | -2.07E+00 | -3.96E+00 | -3.14E+00 | -9.87E-01 |
| Loganin pentaacetate Esi-6.662001 | 1.25E-02 | -9.41E+00 | -4.54E+00 | -1.37E+01 | -5.16E+00 |
| Tetracenomycin X Esi-9.104002 | 3.34E-02 | -8.98E+00 | -3.88E+00 | -1.37E+01 | -4.25E+00 |
| C41 H83 N4 O4 | 2.79E-02 | 3.02E+00 | 4.09E+00 | 1.50E+00 | 4.55E+00 |
| Garcilivin A Esi-6.475999 | 3.32E-02 | -2.15E+00 | -3.90E+00 | -3.28E+00 | -1.02E+00 |
| Amprenavir Esi-16.204002 | 5.44E-03 | -1.07E+01 | -4.95E+00 | -1.51E+01 | -6.29E+00 |
| AS 1-1 Esi-15.851002 | 1.99E-02 | -8.59E+00 | -4.18E+00 | -1.28E+01 | -4.39E+00 |
| Leonoside B | 1.55E-02 | -4.52E+00 | -4.56E+00 | -6.57E+00 | -2.46E+00 |
| PS(18:0/18:1(9Z)) | 1.96E-02 | 5.39E+00 | 3.86E+00 | 2.61E+00 | 8.17E+00 |
| UDP-N-acetyl-D-galactosaminuronic acid Esi-14.945005 | 6.65E-04 | 1.15E+01 | 6.45E+00 | 7.83E+00 | 1.52E+01 |
| Ononin Esi-2.2549996 | 2.56E-04 | -3.56E+00 | -6.51E+00 | -4.68E+00 | -2.44E+00 |
| Glaucarubol 15-O-beta-D-glucopyranoside Esi-5.481002 | 3.10E-02 | -1.85E+00 | -3.98E+00 | -2.80E+00 | -8.98E-01 |
| UDP-N-acetyl-D-galactosaminuronic acid Esi-14.989999 | 6.98E-04 | 1.28E+01 | 6.10E+00 | 8.48E+00 | 1.71E+01 |
| 6,8a-Seco-6,8a-deoxy-5-oxoavermectin ''1a'' aglycone Esi-15.7739935 | 4.29E-02 | 3.99E+00 | 3.68E+00 | 1.77E+00 | 6.20E+00 |
| 10-Acetoxyoleuropein Esi-6.1239967 | 4.63E-03 | -2.62E+00 | -5.03E+00 | -3.68E+00 | -1.55E+00 |
| (R)-1-O-b-D-glucopyranosyl-1,3-octanediol Esi-5.475002 | 7.32E-03 | -3.14E+00 | -4.91E+00 | -4.45E+00 | -1.83E+00 |
| Khellol glucoside Esi-12.604003 | 3.45E-02 | -9.06E+00 | -3.85E+00 | -1.39E+01 | -4.23E+00 |
| Veranisatin C Esi-4.5540004 | 4.15E-02 | -3.59E+00 | -3.77E+00 | -5.56E+00 | -1.63E+00 |
| Acanthifolicin Esi-14.933003 | 4.89E-02 | 3.25E+00 | 3.67E+00 | 1.43E+00 | 5.08E+00 |
| Lamioside Esi-5.141999 | 1.75E-02 | -2.56E+00 | -4.39E+00 | -3.76E+00 | -1.36E+00 |
| C18 H16 N7 O2 Esi-8.0859995 | 2.37E-02 | 9.29E+00 | 4.63E+00 | 5.02E+00 | 1.36E+01 |
| PE(20:4(8Z,11Z,14Z,17Z)/15:0) Esi-16.389 | 3.44E-04 | 2.31E+00 | 5.63E+00 | 1.49E+00 | 3.13E+00 |
| C14 H2 N3 O12 | 1.61E-02 | -8.64E+00 | -4.50E+00 | -1.26E+01 | -4.67E+00 |
| Lucidone B | 1.07E-02 | -9.01E+00 | -4.69E+00 | -1.30E+01 | -5.06E+00 |
| Caffeic acid 3-sulfate Esi-4.2020006 | 1.92E-03 | -3.99E+00 | -5.55E+00 | -5.47E+00 | -2.52E+00 |
| Ethyl 7-epi-12-hydroxyjasmonate glucoside Esi-7.099 | 2.53E-02 | -1.71E+00 | -4.05E+00 | -2.57E+00 | -8.47E-01 |
| Physalin C Esi-5.238002 | 9.11E-03 | -2.08E+00 | -4.68E+00 | -3.00E+00 | -1.17E+00 |
| Cercosporin | 3.57E-04 | -1.20E+01 | -6.33E+00 | -1.59E+01 | -8.15E+00 |
| Morroniside Esi-5.3659987 | 2.53E-02 | -9.22E+00 | -4.06E+00 | -1.39E+01 | -4.58E+00 |
| Anhwiedelphinine Esi-16.200996 | 1.73E-02 | 6.26E+00 | 4.39E+00 | 3.32E+00 | 9.20E+00 |
| Se-Adenosylselenomethionine Esi-5.514 | 3.14E-02 | -9.12E+00 | -3.93E+00 | -1.39E+01 | -4.38E+00 |
| Kelampayoside A Esi-3.7449992 | 3.36E-02 | -8.08E+00 | -4.33E+00 | -1.21E+01 | -4.10E+00 |
| PE(20:0/P-18:1(11Z)) | 7.52E-03 | 5.76E+00 | 4.29E+00 | 3.10E+00 | 8.43E+00 |
| Ligustroside Esi-5.2760005 | 3.23E-02 | -1.24E+00 | -3.94E+00 | -1.88E+00 | -5.94E-01 |
| C15 H16 N7 O4 | 4.48E-02 | -8.99E+00 | -3.67E+00 | -1.40E+01 | -3.98E+00 |
| 4,4''-bis(N-feruloyl)serotonin | 1.80E-02 | -2.18E+00 | -4.28E+00 | -3.23E+00 | -1.14E+00 |
| Hygromycin A | 8.41E-08 | -1.34E+01 | -1.02E+01 | -1.61E+01 | -1.07E+01 |
| Phloroacetophenone 6'-[xylosyl-(1-6)-glucoside] Esi-3.9689999 | 1.10E-02 | -2.39E+00 | -4.55E+00 | -3.47E+00 | -1.32E+00 |
| C37 H81 N21 O | 3.34E-02 | 9.10E+00 | 4.07E+00 | 4.45E+00 | 1.37E+01 |
| Landomycin H | 4.80E-04 | -2.81E+00 | -6.24E+00 | -3.74E+00 | -1.89E+00 |
| C21 H22 N14 O6 | 3.37E-03 | -5.52E+00 | -5.30E+00 | -7.65E+00 | -3.38E+00 |
| Landomycin H Esi-3.185999 | 6.11E-04 | -3.44E+00 | -6.44E+00 | -4.54E+00 | -2.33E+00 |
| Chlorobenzilate Esi-4.6090007 | 4.11E-02 | -8.12E+00 | -3.79E+00 | -1.25E+01 | -3.70E+00 |
| Lucuminic acid Esi-4.0010014 | 4.49E-02 | -1.34E+00 | -3.79E+00 | -2.07E+00 | -6.06E-01 |
| Ganoderiol I Esi-14.431 | 4.37E-02 | -8.54E+00 | -3.67E+00 | -1.33E+01 | -3.78E+00 |
| C22 H19 N15 O3 | 2.52E-02 | 8.68E+00 | 4.10E+00 | 4.34E+00 | 1.30E+01 |
| Leiocarposide Esi-5.834 | 3.47E-02 | -3.71E+00 | -3.90E+00 | -5.67E+00 | -1.75E+00 |
| PI(20:3(5Z,8Z,11Z)/18:2(9Z,12Z)) Esi-15.946997 | 1.52E-02 | 6.42E+00 | 4.13E+00 | 3.30E+00 | 9.55E+00 |
| Isofucosterol 3-O-[6-O-(9-Octadecenoyl)-b-D-glucopyranoside] Esi-15.041008 | 4.28E-03 | 2.89E+00 | 5.38E+00 | 1.78E+00 | 4.00E+00 |
| 12-Oxo-2,3-dinor-10,15-phytodienoic acid Esi-14.130998 | 3.38E-02 | -7.83E+00 | -4.32E+00 | -1.17E+01 | -3.97E+00 |
| Gentiacaulein | 3.70E-02 | -8.28E+00 | -3.76E+00 | -1.28E+01 | -3.78E+00 |
| 5a,11a-Dehydrooxytetracycline | 3.14E-02 | -4.00E+00 | -4.33E+00 | -5.95E+00 | -2.05E+00 |
| Monuron | 2.70E-02 | -3.39E+00 | -4.06E+00 | -5.10E+00 | -1.68E+00 |
| Candesartan cilextil Esi-11.627006 | 4.21E-02 | 2.14E+00 | 3.79E+00 | 9.75E-01 | 3.31E+00 |
| 2'',4''-Diacetylafzelin Esi-6.9439993 | 4.41E-02 | -3.95E+00 | -3.73E+00 | -6.13E+00 | -1.77E+00 |
| 5''-(4-Hydroxy-(E)-cinnamoyl) alpha-L-arabinofuranosyl-(1-3)-beta-D-xylopyranosyl-(1-4)-D-xylopyranoside Esi-0.8600002 | 3.68E-02 | -7.55E+00 | -4.14E+00 | -1.14E+01 | -3.69E+00 |
| Hordatine A glucoside Esi-13.386999 | 3.34E-02 | 7.91E+00 | 4.35E+00 | 4.03E+00 | 1.18E+01 |
| Cordeauxione Esi-6.686002 | 4.84E-02 | 2.02E+00 | 3.70E+00 | 8.96E-01 | 3.15E+00 |
| Cnicin | 3.30E-02 | 8.31E+00 | 4.10E+00 | 4.09E+00 | 1.25E+01 |
| PE(20:0/P-18:1(11Z)) Esi-15.748996 | 8.84E-04 | 6.48E+00 | 5.07E+00 | 3.94E+00 | 9.02E+00 |
| (14alpha,17beta,20S,22R)-14,20-Epoxy-17-hydroxy-1-oxowitha-3,5,24-trienolide Esi-15.336 | 1.07E-02 | -3.55E+00 | -4.66E+00 | -5.11E+00 | -1.98E+00 |
| Xerocomic acid Esi-5.056 | 1.16E-05 | -1.22E+01 | -7.91E+00 | -1.53E+01 | -9.01E+00 |
| Nagilactone C Esi-10.444 | 3.66E-02 | 8.18E+00 | 3.77E+00 | 3.74E+00 | 1.26E+01 |
| Delphin | 3.91E-02 | -8.50E+00 | -3.73E+00 | -1.32E+01 | -3.84E+00 |
| (S)-N-[3-(3,4-Methylenedioxyphenyl)-2-(acetylthio)methyl-1-oxoprolyl]-(S)-alanine benzyl ester | 4.30E-02 | -8.32E+00 | -3.71E+00 | -1.29E+01 | -3.72E+00 |
| Luteone 7-glucoside Esi-4.925002 | 1.36E-03 | -2.52E+00 | -5.69E+00 | -3.42E+00 | -1.61E+00 |
| Bruceantin Esi-7.6549997 | 7.81E-03 | -1.72E+00 | -4.74E+00 | -2.46E+00 | -9.78E-01 |
| C36 H52 N10 | 4.87E-02 | -1.07E+00 | -3.61E+00 | -1.68E+00 | -4.64E-01 |
| Leucomycin A7 Esi-13.694 | 4.54E-02 | 7.74E+00 | 3.68E+00 | 3.43E+00 | 1.20E+01 |
| C42 H79 N8 Esi-16.270998 | 8.57E-03 | 1.00E+01 | 4.70E+00 | 5.67E+00 | 1.44E+01 |
| > limit Esi-14.300998 | 1.94E-02 | 4.56E+00 | 4.19E+00 | 2.34E+00 | 6.79E+00 |
| 3beta,15alpha-Diacetoxylanosta-8,24-dien-26-oic acid Esi-16.579998 | 8.06E-03 | 3.92E+00 | 5.04E+00 | 2.30E+00 | 5.53E+00 |
| Pteleine Esi-3.3379996 | 3.47E-02 | -8.33E+00 | -3.90E+00 | -1.27E+01 | -3.93E+00 |
| Garcimangosone D Esi-0.8479997 | 3.12E-02 | -2.13E+00 | -4.05E+00 | -3.21E+00 | -1.04E+00 |
| 2,4-Bis(acetamido)-2,4,6-trideoxy-beta-L-altropyranose Esi-3.0390007 | 3.64E-02 | -8.21E+00 | -4.18E+00 | -1.24E+01 | -4.04E+00 |
| NPC | 9.63E-04 | -1.76E+00 | -5.93E+00 | -2.36E+00 | -1.15E+00 |
| (S)-N-[3-(3,4-Methylenedioxyphenyl)-2-(acetylthio)methyl-1-oxoprolyl]glycine benzyl ester | 1.86E-02 | -8.66E+00 | -4.60E+00 | -1.26E+01 | -4.71E+00 |
| N-desmethylimatinib Esi-13.619002 | 1.07E-02 | -9.98E+00 | -4.72E+00 | -1.43E+01 | -5.62E+00 |
| Stigmatellin X Esi-13.869997 | 1.83E-02 | -9.53E+00 | -4.53E+00 | -1.39E+01 | -5.14E+00 |
| Lamiide Esi-2.9430006 | 3.85E-02 | -1.41E+00 | -3.73E+00 | -2.18E+00 | -6.38E-01 |
| Harpagoside Esi-4.374 | 2.14E-04 | -1.22E+01 | -8.46E+00 | -1.53E+01 | -9.17E+00 |
| C23 H28 N6 O4 Esi-14.939001 | 1.59E-02 | -4.65E+00 | -4.87E+00 | -6.67E+00 | -2.63E+00 |
| PE(22:2(13Z,16Z)/P-18:1(11Z)) Esi-15.733001 | 1.07E-02 | 5.27E+00 | 4.14E+00 | 2.74E+00 | 7.80E+00 |
| Coproporphyrin I | 3.54E-02 | -7.90E+00 | -3.79E+00 | -1.22E+01 | -3.64E+00 |
| (8Z,11Z,14Z,17Z)-Icosatetraenoyl-CoA Esi-14.254002 | 2.44E-02 | 2.82E+00 | 3.86E+00 | 1.35E+00 | 4.28E+00 |
| Shanzhiside Esi-4.5930014 | 1.44E-02 | -2.81E+00 | -4.41E+00 | -4.12E+00 | -1.51E+00 |
| C31 H22 N7 | 1.45E-02 | -1.87E+00 | -4.42E+00 | -2.74E+00 | -1.00E+00 |
| > limit Esi-16.209995 | 2.52E-02 | 7.99E+00 | 4.06E+00 | 3.97E+00 | 1.20E+01 |
| PC(20:1(11Z)/18:1(9Z)) Esi-16.724998 | 3.28E-02 | 2.34E+00 | 3.57E+00 | 1.04E+00 | 3.64E+00 |
| Janthitrem G Esi-12.208992 | 1.40E-02 | 1.84E+00 | 4.46E+00 | 9.93E-01 | 2.68E+00 |
| Lucidenic acid J Esi-13.8080015 | 3.44E-02 | 9.14E+00 | 3.81E+00 | 4.24E+00 | 1.40E+01 |
| Decuroside III Esi-8.600999 | 3.35E-02 | -7.93E+00 | -4.33E+00 | -1.18E+01 | -4.03E+00 |
| LysoPE(22:0/0:0) Esi-13.287002 | 3.34E-02 | -8.17E+00 | -4.35E+00 | -1.22E+01 | -4.17E+00 |
| PE(15:0/P-18:0) Esi-15.036004 | 3.57E-02 | -4.64E+00 | -3.51E+00 | -7.27E+00 | -2.02E+00 |
| Lepidimoic acid | 6.33E-03 | -1.61E+00 | -5.16E+00 | -2.26E+00 | -9.65E-01 |
| Cartormin Esi-5.3150015 | 1.55E-02 | -1.74E+00 | -4.38E+00 | -2.55E+00 | -9.26E-01 |
| Citrusin B Esi-6.4849997 | 3.36E-02 | -2.07E+00 | -3.94E+00 | -3.15E+00 | -9.88E-01 |
| > limit Esi-16.231997 | 1.80E-02 | 8.25E+00 | 4.28E+00 | 4.30E+00 | 1.22E+01 |
| Cinncassiol D2 glucoside Esi-8.619002 | 3.34E-02 | -8.43E+00 | -3.86E+00 | -1.29E+01 | -3.96E+00 |
| DG(14:0/14:0/0:0) Esi-14.076003 | 3.63E-02 | 4.99E+00 | 3.50E+00 | 2.16E+00 | 7.83E+00 |
| C33 H20 N18 O2 | 3.34E-02 | -2.03E+00 | -3.88E+00 | -3.10E+00 | -9.57E-01 |
| PE(15:0/22:2(13Z,16Z)) | 4.44E-03 | 5.59E+00 | 4.58E+00 | 3.16E+00 | 8.02E+00 |
| C40 H11 N7 O3 | 3.39E-03 | 6.74E+00 | 5.33E+00 | 4.14E+00 | 9.34E+00 |
| PG(18:0/18:1(11Z)) Esi-14.945005 | 3.63E-02 | -8.01E+00 | -3.77E+00 | -1.24E+01 | -3.67E+00 |
| 6-Acetylpicropolin Esi-1.0429999 | 1.07E-02 | -9.09E+00 | -4.75E+00 | -1.30E+01 | -5.14E+00 |
| C21 H22 N14 O6 Esi-2.7839987 | 2.29E-03 | -5.33E+00 | -5.76E+00 | -7.24E+00 | -3.42E+00 |
| 1-O-Galloyl-beta-D-glucose Esi-1.3030003 | 3.18E-02 | -1.98E+00 | -3.97E+00 | -3.00E+00 | -9.55E-01 |
| PE(20:0/P-18:1(11Z)) Esi-15.419999 | 2.87E-02 | 4.73E+00 | 3.70E+00 | 2.18E+00 | 7.27E+00 |
| Disenecionyl cis-khellactone Esi-14.0079975 | 4.20E-02 | -2.03E+00 | -3.74E+00 | -3.14E+00 | -9.15E-01 |
| Phosphatidylserine | 3.94E-02 | -1.42E+00 | -3.77E+00 | -2.20E+00 | -6.47E-01 |
| E3040 glucuronide | 2.46E-03 | -1.10E+01 | -6.67E+00 | -1.45E+01 | -7.50E+00 |
| Flavine mononucleotide (FMN) Esi-4.695 | 8.41E-11 | 7.78E+00 | 8.32E+00 | 5.94E+00 | 9.63E+00 |
| 7-Deoxyloganate Esi-6.2319994 | 1.61E-02 | -9.44E+00 | -4.49E+00 | -1.38E+01 | -5.09E+00 |
| C23 H32 N2 O2 | 3.47E-02 | -9.39E+00 | -3.82E+00 | -1.44E+01 | -4.36E+00 |
| Verproside | 2.89E-02 | -1.73E+00 | -3.99E+00 | -2.61E+00 | -8.41E-01 |
| (7E,7'R,8'R)-e-Viniferin 3',5'-diglucoside | 2.80E-02 | -3.88E+00 | -4.08E+00 | -5.83E+00 | -1.92E+00 |
| Doxycycline Esi-4.583 | 1.65E-02 | -9.26E+00 | -4.95E+00 | -1.32E+01 | -5.27E+00 |
| Nb-trans-Feruloylserotonin glucoside Esi-11.467 | 3.50E-02 | 9.00E+00 | 4.00E+00 | 4.31E+00 | 1.37E+01 |
| C13 H2 N4 O5 S Esi-12.509002 | 3.34E-02 | -7.99E+00 | -4.01E+00 | -1.21E+01 | -3.87E+00 |
| Isofucosterol 3-O-[6-O-(9-Octadecenoyl)-b-D-glucopyranoside] Esi-15.008998 | 4.56E-02 | 2.60E+00 | 3.64E+00 | 1.14E+00 | 4.06E+00 |
| C17 H20 N7 O7 | 3.49E-02 | -8.50E+00 | -3.82E+00 | -1.31E+01 | -3.94E+00 |
| PC(18:0/P-18:0) Esi-15.851003 | 1.45E-02 | -3.58E+00 | -3.89E+00 | -5.39E+00 | -1.76E+00 |
| C22 H32 N7 O4 | 3.63E-02 | 2.20E+00 | 3.78E+00 | 1.01E+00 | 3.40E+00 |
| Chlorobenzilate Esi-3.9719996 | 1.99E-02 | -2.28E+00 | -4.19E+00 | -3.39E+00 | -1.17E+00 |
| C29 H26 N21 O | 3.44E-02 | -1.40E+00 | -3.82E+00 | -2.14E+00 | -6.49E-01 |
| C19 H28 N7 O4 Esi-7.2769976 | 3.84E-02 | -1.89E+00 | -3.74E+00 | -2.92E+00 | -8.56E-01 |
| Somniferine Esi-6.602998 | 2.75E-02 | -2.03E+00 | -4.01E+00 | -3.06E+00 | -9.97E-01 |
| C14 H14 N7 O5 | 3.47E-02 | -8.41E+00 | -3.95E+00 | -1.28E+01 | -4.00E+00 |
| C27 H40 N3 O3 | 2.12E-02 | 8.84E+00 | 4.17E+00 | 4.50E+00 | 1.32E+01 |
| 3'-Ketolactose Esi-1.0509999 | 1.65E-02 | -1.31E+00 | -4.33E+00 | -1.93E+00 | -6.92E-01 |
| (14alpha,17beta,20S,22R)-14,20-Epoxy-17-hydroxy-1-oxowitha-3,5,24-trienolide Esi-15.314001 | 5.78E-03 | -3.85E+00 | -5.13E+00 | -5.40E+00 | -2.30E+00 |
| PE(14:0/16:1(9Z)) Esi-14.845999 | 3.34E-02 | 6.19E+00 | 3.59E+00 | 2.76E+00 | 9.62E+00 |
| 1D-Myo-inositol 3,4-bisphosphate Esi-13.791001 | 4.61E-04 | -8.11E+00 | -5.47E+00 | -1.11E+01 | -5.15E+00 |
| C23 H28 N20 O9 | 3.45E-02 | -2.40E+00 | -3.98E+00 | -3.66E+00 | -1.15E+00 |
| PE(18:3(6Z,9Z,12Z)/18:1(9Z)) Esi-14.338002 | 4.28E-02 | 4.70E+00 | 3.43E+00 | 1.97E+00 | 7.42E+00 |
| 6-Methylthiopurine ribonucleotide Esi-8.343001 | 8.66E-03 | -9.49E+00 | -4.68E+00 | -1.36E+01 | -5.34E+00 |
| C19 H22 N20 O3 | 3.34E-02 | -1.76E+00 | -3.86E+00 | -2.69E+00 | -8.26E-01 |
| C37 H32 N24 O | 1.57E-02 | -1.81E+00 | -4.34E+00 | -2.66E+00 | -9.59E-01 |
| PC(15:0/18:0) | 3.80E-02 | 2.98E+00 | 3.41E+00 | 1.26E+00 | 4.71E+00 |
| Bopindolol | 3.69E-02 | -2.88E+00 | -3.78E+00 | -4.44E+00 | -1.32E+00 |
| (7E,7'R,8'R)-e-Viniferin 3',5'-diglucoside Esi-5.8659983 | 1.69E-03 | -3.99E+00 | -5.70E+00 | -5.42E+00 | -2.55E+00 |
| Loganin pentaacetate Esi-6.389001 | 4.15E-03 | -9.85E+00 | -5.10E+00 | -1.38E+01 | -5.90E+00 |
| Pentacarboxyl porphyrinogen III Esi-7.4940004 | 3.92E-02 | -2.05E+00 | -3.81E+00 | -3.16E+00 | -9.40E-01 |
| Kaempferol 3-O-arabinoside Esi-3.7379992 | 3.73E-02 | -8.63E+00 | -3.78E+00 | -1.33E+01 | -3.95E+00 |
| 3-O-beta-D-Galactopyranosylproanthocyanidin A5' Esi-5.809002 | 2.40E-03 | -2.55E+00 | -5.42E+00 | -3.51E+00 | -1.59E+00 |
| Cinnamtannin A1 Esi-0.8190002 | 3.34E-02 | -8.41E+00 | -3.85E+00 | -1.29E+01 | -3.95E+00 |
| Trichotomine Esi-5.632003 | 2.75E-02 | -2.12E+00 | -4.12E+00 | -3.18E+00 | -1.06E+00 |
| Nitrosylhaem Esi-6.369998 | 7.62E-03 | -2.87E+00 | -4.92E+00 | -4.07E+00 | -1.67E+00 |
| PE(20:4(5Z,8Z,11Z,14Z)/22:6(4Z,7Z,10Z,13Z,16Z,19Z)) Esi-15.696005 | 1.86E-02 | -5.45E+00 | -3.86E+00 | -8.25E+00 | -2.65E+00 |
| 5-Methyl-THF Esi-6.6069984 | 2.92E-03 | -6.07E+00 | -4.83E+00 | -8.59E+00 | -3.56E+00 |
| Destomysin Esi-5.358998 | 2.21E-02 | -2.44E+00 | -4.16E+00 | -3.64E+00 | -1.24E+00 |
| Hesperetin 3',7-O-diglucuronide Esi-7.052001 | 5.28E-03 | -2.37E+00 | -5.11E+00 | -3.33E+00 | -1.42E+00 |
| Repaglinide Esi-7.0249977 | 4.23E-02 | -1.83E+00 | -3.68E+00 | -2.85E+00 | -8.15E-01 |
| 5,6-Dimethoxysterigmatocystin | 3.35E-02 | -2.52E+00 | -3.85E+00 | -3.86E+00 | -1.18E+00 |
| PE(14:0/22:0) | 8.66E-03 | 5.34E+00 | 4.24E+00 | 2.84E+00 | 7.85E+00 |
| Cyclochlorotine | 1.85E-02 | -3.29E+00 | -4.44E+00 | -4.83E+00 | -1.76E+00 |
| 7-(4-Carboxy-3-hydroxy-3-methylbutanoyl)sudachitin 4'-glucoside | 2.77E-02 | -2.22E+00 | -4.02E+00 | -3.35E+00 | -1.09E+00 |
| 10-Acetoxyoleuropein Esi-4.806 | 3.34E-02 | -8.67E+00 | -3.94E+00 | -1.32E+01 | -4.14E+00 |
| 3-O-beta-D-Galactopyranosylcinnamtannin B1 | 3.23E-02 | -2.45E+00 | -3.92E+00 | -3.72E+00 | -1.17E+00 |
| Cartormin Esi-5.498997 | 1.05E-02 | -1.86E+00 | -4.60E+00 | -2.69E+00 | -1.03E+00 |
| Isovitexin 2''-O-[4-hydroxy-(E)-cinnamoyl-(-6)-beta-D-glucopyranosyl] 4'-O-beta-D-glucopyranoside | 3.88E-02 | -3.50E+00 | -3.92E+00 | -5.36E+00 | -1.64E+00 |
| Nb-trans-Feruloylserotonin glucoside Esi-6.8029985 | 3.18E-02 | -2.29E+00 | -3.93E+00 | -3.49E+00 | -1.10E+00 |
| Cassiaside B2 Esi-4.5529995 | 7.52E-03 | -5.33E+00 | -4.99E+00 | -7.54E+00 | -3.13E+00 |
| (14alpha,17beta,20S,22R)-14,20-Epoxy-17-hydroxy-1-oxowitha-3,5,24-trienolide Esi-14.895998 | 4.45E-02 | -7.36E+00 | -3.77E+00 | -1.14E+01 | -3.33E+00 |
| C34 H34 N5 O2 | 2.71E-02 | -8.27E+00 | -4.17E+00 | -1.24E+01 | -4.17E+00 |
| PE(20:0/24:0) | 1.93E-02 | 4.90E+00 | 3.87E+00 | 2.38E+00 | 7.42E+00 |
| C14 H27 N12 O2 S4 | 7.57E-03 | -1.61E+00 | -4.78E+00 | -2.29E+00 | -9.20E-01 |
| PE(20:3(5Z,8Z,11Z)/22:4(7Z,10Z,13Z,16Z)) | 6.12E-07 | 8.59E+00 | 7.04E+00 | 6.16E+00 | 1.10E+01 |
| 4',6'-Dihydroxy-2'-methoxyacetophenone 6'-glucoside Esi-5.1370006 | 1.03E-02 | -9.39E+00 | -4.76E+00 | -1.35E+01 | -5.32E+00 |
| 3alpha,7beta,12alpha-Trihydroxy-5alpha-cholan-24-oic Acid Esi-14.940001 | 3.74E-02 | 7.97E+00 | 3.79E+00 | 3.66E+00 | 1.23E+01 |
| (14alpha,17beta,20S,22R)-14,20-Epoxy-17-hydroxy-1-oxowitha-3,5,24-trienolide Esi-14.941 | 4.66E-03 | -6.02E+00 | -5.61E+00 | -8.26E+00 | -3.77E+00 |
| Tolylfluanid Esi-12.507002 | 5.07E-04 | -1.07E+01 | -6.13E+00 | -1.43E+01 | -7.16E+00 |
| C27 H28 N7 Esi-14.501002 | 3.34E-02 | -3.06E+00 | -4.04E+00 | -4.63E+00 | -1.49E+00 |
| PE(18:2(9Z,12Z)/15:0) Esi-16.362 | 4.72E-02 | -4.22E+00 | -3.41E+00 | -6.69E+00 | -1.75E+00 |
| Cinnamtannin A1 Esi-5.464001 | 3.09E-02 | -2.94E+00 | -3.97E+00 | -4.45E+00 | -1.42E+00 |
| Janthitrem G Esi-12.570005 | 1.45E-02 | 1.93E+00 | 4.39E+00 | 1.03E+00 | 2.82E+00 |
| Glucosyl (2E,6E,10x)-10,11-dihydroxy-2,6-farnesadienoate Esi-6.078998 | 1.80E-02 | -1.95E+00 | -4.23E+00 | -2.89E+00 | -1.01E+00 |
| Tetracenomycin X Esi-13.065998 | 1.62E-02 | -9.95E+00 | -4.30E+00 | -1.47E+01 | -5.22E+00 |
| Decuroside III Esi-8.432004 | 4.34E-02 | -2.11E+00 | -3.84E+00 | -3.26E+00 | -9.69E-01 |
| Vitamin D2 3-glucuronide Esi-15.791 | 3.34E-02 | 8.75E+00 | 4.04E+00 | 4.25E+00 | 1.32E+01 |
| C10 H31 Cl N10 O S4 | 1.10E-02 | -9.55E+00 | -4.56E+00 | -1.38E+01 | -5.27E+00 |
| 2-(3,5-Dichlorophenylcarbamoyl)-1,2-dimethylcyclopropane-1-carboxylic acid | 2.24E-02 | -7.19E+00 | -4.25E+00 | -1.07E+01 | -3.70E+00 |
| Kelampayoside A Esi-4.8390007 | 7.64E-03 | -2.95E+00 | -4.88E+00 | -4.19E+00 | -1.70E+00 |
| C31 H30 N13 O Esi-14.666008 | 3.35E-02 | 2.36E+00 | 3.94E+00 | 1.13E+00 | 3.60E+00 |
| PE(22:2(13Z,16Z)/24:1(15Z)) Esi-15.794004 | 2.80E-02 | 4.40E+00 | 3.73E+00 | 2.05E+00 | 6.75E+00 |
| C37 H32 N24 O Esi-7.9379997 | 1.38E-02 | -1.84E+00 | -4.42E+00 | -2.69E+00 | -9.90E-01 |
| Cortolone-3-glucuronide Esi-8.115 | 3.32E-02 | -2.87E+00 | -3.93E+00 | -4.36E+00 | -1.37E+00 |
| 3,4,5-Trimethoxyphenyl glucoside Esi-4.294001 | 3.20E-02 | -2.09E+00 | -3.92E+00 | -3.17E+00 | -1.00E+00 |
| PS(18:0/20:4(8Z,11Z,14Z,17Z)) Esi-14.150999 | 1.58E-02 | 6.03E+00 | 3.95E+00 | 2.99E+00 | 9.07E+00 |
| (3b,6b,8b,12a)-8,12-Epoxy-7(11)-eremophilene-6-angeloyloxy-8,12-dimethoxy-3-ol Esi-14.1530075 | 1.90E-03 | 3.27E+00 | 6.16E+00 | 2.16E+00 | 4.37E+00 |
| 8-Pentanoylneosolaniol Esi-14.640999 | 2.14E-02 | -9.26E+00 | -4.44E+00 | -1.36E+01 | -4.90E+00 |
| 4-Hydroxy-3-(16-methylheptadecyl)-2H-pyran-2-one Esi-15.296998 | 2.84E-02 | -8.74E+00 | -4.00E+00 | -1.32E+01 | -4.27E+00 |
| PE(20:0/dm18:0) Esi-15.829994 | 3.34E-03 | 5.83E+00 | 4.66E+00 | 3.34E+00 | 8.32E+00 |
| PE(20:4(8Z,11Z,14Z,17Z)/20:2(11Z,14Z)) | 3.32E-02 | -8.30E-01 | -3.59E+00 | -1.29E+00 | -3.71E-01 |
| C28 H29 N9 O2 S | 2.14E-02 | -1.35E+00 | -4.15E+00 | -2.02E+00 | -6.88E-01 |
| 3-O-beta-D-Galactopyranosylcinnamtannin B1 Esi-5.272002 | 4.95E-02 | -2.71E+00 | -3.60E+00 | -4.24E+00 | -1.17E+00 |
| Hemin | 3.26E-02 | -8.40E+00 | -3.98E+00 | -1.27E+01 | -4.06E+00 |
| C17 H48 N16 O6 S | 3.34E-02 | -8.51E+00 | -4.16E+00 | -1.28E+01 | -4.23E+00 |
| Candesartan cilextil Esi-11.635995 | 4.84E-02 | 3.29E+00 | 3.67E+00 | 1.45E+00 | 5.14E+00 |
| Usambarensine Esi-14.688 | 4.51E-02 | -9.35E+00 | -3.67E+00 | -1.46E+01 | -4.13E+00 |
| PE(14:0/P-16:0) | 5.01E-03 | 7.33E+00 | 4.46E+00 | 4.07E+00 | 1.06E+01 |
| Avermectin A1a aglycone Esi-13.645997 | 3.38E-02 | 8.54E+00 | 3.83E+00 | 3.99E+00 | 1.31E+01 |
| Glaucarubolone 15-O-beta-D-glucopyranoside Esi-6.9299965 | 3.35E-02 | -1.62E+00 | -3.88E+00 | -2.48E+00 | -7.64E-01 |
| Lusitanicoside Esi-5.2960014 | 3.32E-02 | -2.16E+00 | -3.89E+00 | -3.30E+00 | -1.03E+00 |
| Spinacetin 3-(2''-feruloylgentiobioside) Esi-4.380998 | 4.34E-02 | -3.42E+00 | -3.78E+00 | -5.29E+00 | -1.55E+00 |
| PIP2(16:0/18:1(11Z)) Esi-9.864999 | 1.16E-03 | -4.17E+00 | -5.20E+00 | -5.77E+00 | -2.56E+00 |
| PC(20:3(5Z,8Z,11Z)/20:0) | 3.76E-02 | 3.30E+00 | 3.41E+00 | 1.39E+00 | 5.21E+00 |
| PE(22:4(7Z,10Z,13Z,16Z)/22:6(4Z,7Z,10Z,13Z,16Z,19Z)) | 1.51E-04 | -7.05E+00 | -5.61E+00 | -9.55E+00 | -4.56E+00 |
| C32 H42 N3 O25 | 3.11E-02 | -8.66E+00 | -3.96E+00 | -1.31E+01 | -4.19E+00 |
| Phytolaccoside B Esi-10.1329975 | 3.85E-02 | -1.77E+00 | -3.73E+00 | -2.74E+00 | -8.02E-01 |
| 1-O-Galloyl-beta-D-glucose Esi-1.7700001 | 4.12E-02 | -1.99E+00 | -3.71E+00 | -3.09E+00 | -8.94E-01 |
| Spinacetin 3-(2''-feruloylgentiobioside) Esi-4.2549996 | 1.75E-02 | -4.58E+00 | -4.44E+00 | -6.72E+00 | -2.44E+00 |
| Cinnamtannin A1 Esi-5.4179997 | 3.09E-02 | -2.68E+00 | -3.97E+00 | -4.07E+00 | -1.30E+00 |
| 3-Oxotetradecanoyl-CoA Esi-6.8879995 | 3.34E-02 | -1.69E+00 | -3.59E+00 | -2.63E+00 | -7.55E-01 |
| Pachymic acid Esi-15.762001 | 6.82E-03 | 3.26E+00 | 4.84E+00 | 1.88E+00 | 4.63E+00 |
| DG(22:6(4Z,7Z,10Z,13Z,16Z,19Z)/20:3(5Z,8Z,11Z)/0:0) Esi-16.088005 | 1.58E-02 | -5.51E+00 | -4.44E+00 | -8.07E+00 | -2.96E+00 |
| Furohyperforin Esi-15.510002 | 1.56E-02 | 9.75E+00 | 4.34E+00 | 5.16E+00 | 1.43E+01 |
| PE(15:0/20:3(5Z,8Z,11Z)) Esi-15.494001 | 4.95E-02 | 4.26E+00 | 3.41E+00 | 1.76E+00 | 6.75E+00 |
| PE(14:1(9Z)/15:0) | 3.67E-03 | 3.90E+00 | 4.64E+00 | 2.23E+00 | 5.58E+00 |
| PE(14:0/16:1(9Z)) Esi-15.362001 | 1.24E-02 | 5.04E+00 | 4.16E+00 | 2.62E+00 | 7.46E+00 |
| Phenothrin Esi-14.609994 | 3.46E-02 | 6.18E+00 | 3.82E+00 | 2.87E+00 | 9.49E+00 |
| C30 H30 N20 O3 | 3.34E-02 | -2.03E+00 | -3.85E+00 | -3.11E+00 | -9.54E-01 |
| C36 H77 N24 | 2.76E-03 | 2.59E+00 | 5.33E+00 | 1.60E+00 | 3.58E+00 |
| 14-Deacetylnudicauline Esi-5.3020015 | 2.50E-02 | -2.52E+00 | -4.11E+00 | -3.78E+00 | -1.26E+00 |
| C35 H28 N21 O4 Esi-6.6849985 | 4.05E-02 | -1.93E+00 | -3.82E+00 | -2.97E+00 | -8.84E-01 |
| Clerodendrin A Esi-8.086999 | 4.44E-02 | -7.87E+00 | -3.69E+00 | -1.22E+01 | -3.49E+00 |
| > limit Esi-5.9629984 | 1.37E-02 | -5.57E+00 | -4.46E+00 | -8.12E+00 | -3.01E+00 |
| 2-(4-Allyl-2,6-dimethoxyphenoxy)-1-(3-hydroxy-4,5-dimethoxyphenyl)-1-propanol | 7.52E-03 | 1.01E+01 | 5.68E+00 | 6.29E+00 | 1.39E+01 |
| Cinnamtannin A2 Esi-5.3240013 | 1.58E-02 | -3.56E+00 | -4.38E+00 | -5.22E+00 | -1.89E+00 |
| Kaempferol 3-[2''-(6'''-coumaroylglucosyl)-rhamnoside] 7-glucoside Esi-4.8910017 | 1.71E-02 | -2.93E+00 | -4.42E+00 | -4.30E+00 | -1.56E+00 |
| PE(14:1(9Z)/16:1(9Z)) Esi-15.371001 | 7.68E-04 | 5.83E+00 | 5.16E+00 | 3.58E+00 | 8.07E+00 |
| PE(18:2(9Z,12Z)/18:1(11Z)) Esi-16.838 | 4.54E-02 | -3.99E+00 | -3.44E+00 | -6.30E+00 | -1.67E+00 |
| 3beta,15alpha-Diacetoxylanosta-8,24-dien-26-oic acid Esi-15.79101 | 1.99E-02 | 2.68E+00 | 4.28E+00 | 1.39E+00 | 3.97E+00 |
| > limit Esi-15.9070015 | 6.91E-06 | 1.07E+01 | 8.13E+00 | 8.01E+00 | 1.34E+01 |
| PE(14:0/22:0) Esi-15.308998 | 3.34E-02 | 2.50E+00 | 3.62E+00 | 1.12E+00 | 3.87E+00 |
| Reduced flavine adenine dinucleotide (FADH2) | 3.30E-02 | 2.77E+00 | 3.61E+00 | 1.25E+00 | 4.30E+00 |
| 2-Octaprenyl-3-methyl-6-methoxy-1,4-benzoquinone Esi-15.764 | 2.52E-02 | 2.82E+00 | 4.15E+00 | 1.42E+00 | 4.23E+00 |
| PI(16:0/18:1(11Z)) Esi-15.311002 | 4.92E-05 | -8.94E+00 | -7.09E+00 | -1.15E+01 | -6.37E+00 |
| PE(14:1(9Z)/15:0) Esi-13.4140005 | 2.39E-02 | 3.96E+00 | 3.90E+00 | 1.92E+00 | 6.01E+00 |
| Androstane-3,17-diol dipropionate Esi-15.897998 | 3.71E-02 | 8.28E+00 | 3.90E+00 | 3.88E+00 | 1.27E+01 |
| PE(18:4(6Z,9Z,12Z,15Z)/P-16:0) | 1.10E-04 | 8.60E+00 | 5.69E+00 | 5.60E+00 | 1.16E+01 |
| C20 H30 N7 O4 Esi-8.306 | 7.62E-03 | -2.30E+00 | -4.77E+00 | -3.29E+00 | -1.32E+00 |
| C22 H42 N3 O7 S | 2.23E-02 | 9.17E+00 | 4.12E+00 | 4.62E+00 | 1.37E+01 |
| 3'-Keto-3'-deoxy-AMP | 1.29E-02 | -8.25E+00 | -4.66E+00 | -1.19E+01 | -4.59E+00 |
| Leiocarposide Esi-5.9149976 | 3.31E-02 | -6.27E+00 | -4.21E+00 | -9.39E+00 | -3.14E+00 |
| Cinitapride Esi-8.354003 | 1.90E-02 | -2.35E+00 | -4.34E+00 | -3.47E+00 | -1.24E+00 |
| DG(22:6(4Z,7Z,10Z,13Z,16Z,19Z)/20:3(5Z,8Z,11Z)/0:0) Esi-16.02 | 1.20E-02 | -5.05E+00 | -4.56E+00 | -7.32E+00 | -2.78E+00 |
| Leonoside B Esi-4.192001 | 5.53E-03 | -4.44E+00 | -5.01E+00 | -6.26E+00 | -2.62E+00 |
| DG(22:6(4Z,7Z,10Z,13Z,16Z,19Z)/20:3(5Z,8Z,11Z)/0:0) Esi-15.927 | 3.40E-02 | -5.21E+00 | -4.00E+00 | -7.91E+00 | -2.51E+00 |
| PE(15:0/20:0) Esi-13.024998 | 1.42E-02 | 7.16E+00 | 4.00E+00 | 3.60E+00 | 1.07E+01 |
| PE(20:0/18:3(6Z,9Z,12Z)) Esi-14.050997 | 5.51E-03 | 3.85E+00 | 4.45E+00 | 2.13E+00 | 5.56E+00 |
| PE(15:0/20:0) Esi-12.977998 | 1.10E-02 | 7.33E+00 | 4.11E+00 | 3.79E+00 | 1.09E+01 |
| Oleandolide Esi-8.345 | 4.45E-02 | -8.96E+00 | -3.67E+00 | -1.40E+01 | -3.96E+00 |
| Kiwiionoside Esi-5.7359996 | 4.38E-02 | -9.47E+00 | -3.77E+00 | -1.47E+01 | -4.28E+00 |
| Camptothecin Esi-4.966002 | 3.27E-02 | -8.74E+00 | -4.25E+00 | -1.31E+01 | -4.41E+00 |
| Acuminoside Esi-8.835002 | 7.02E-03 | -1.03E+01 | -4.95E+00 | -1.45E+01 | -6.00E+00 |
| Methyl-2-alpha-L-fucopyranosyl-beta-D-galactoside Esi-2.5540004 | 3.10E-02 | -9.95E+00 | -4.06E+00 | -1.50E+01 | -4.90E+00 |
| 4-Hydroxybenzenesulfonic acid | 1.62E-03 | -1.23E+01 | -5.83E+00 | -1.66E+01 | -7.93E+00 |
| PC(22:5(7Z,10Z,13Z,16Z,19Z)/18:4(6Z,9Z,12Z,15Z)) | 2.39E-02 | -3.53E+00 | -3.67E+00 | -5.42E+00 | -1.63E+00 |
| Saxitoxin Esi-4.029 | 3.23E-02 | -9.55E+00 | -3.93E+00 | -1.45E+01 | -4.58E+00 |
| Purothionin AII Esi-6.925002 | 1.71E-02 | -9.43E+00 | -4.80E+00 | -1.36E+01 | -5.27E+00 |
| 1-(3-Methyl-2-butenoyl)-6-apiosylglucose Esi-2.3989997 | 1.27E-02 | -1.07E+01 | -4.53E+00 | -1.56E+01 | -5.88E+00 |
| PI(22:4(10Z,13Z,16Z,19Z)/18:0) Esi-16.485994 | 1.75E-03 | -8.21E+00 | -5.15E+00 | -1.14E+01 | -5.01E+00 |
| Hydroxysafflor yellow A Esi-6.06 | 3.31E-03 | -1.22E+01 | -6.35E+00 | -1.63E+01 | -8.08E+00 |
| Diphenoxylic acid(DPA) | 3.45E-02 | -8.33E+00 | -3.80E+00 | -1.28E+01 | -3.86E+00 |
| 1-(3-Methyl-2-butenoyl)-6-apiosylglucose Esi-1.7989999 | 2.89E-02 | -9.74E+00 | -4.01E+00 | -1.47E+01 | -4.77E+00 |
| Pantoyllactone glucoside Esi-3.243 | 1.61E-02 | -9.69E+00 | -4.36E+00 | -1.42E+01 | -5.13E+00 |
| (E)-Resveratrol 3-glucoside 4'-sulfate | 8.98E-03 | -9.63E+00 | -4.67E+00 | -1.39E+01 | -5.42E+00 |
| PE(16:1(9Z)/22:6(4Z,7Z,10Z,13Z,16Z,19Z)) | 4.66E-02 | -5.06E+00 | -3.38E+00 | -8.03E+00 | -2.09E+00 |
| 6-Deoxyerythronolide B Esi-10.975999 | 3.40E-02 | -8.74E+00 | -3.85E+00 | -1.34E+01 | -4.09E+00 |
| 1-(3-Methyl-2-butenoyl)-6-apiosylglucose Esi-1.6730002 | 4.01E-04 | -1.21E+01 | -6.27E+00 | -1.61E+01 | -8.19E+00 |
| 8-Acetoxy-4'-methoxypinoresinol 4-glucoside Esi-6.8099995 | 1.28E-02 | -1.02E+01 | -4.54E+00 | -1.48E+01 | -5.58E+00 |
| 3'-Keto-3'-deoxy-AMP Esi-12.678 | 9.16E-03 | -9.06E+00 | -4.89E+00 | -1.29E+01 | -5.22E+00 |
| Monocrotaline Esi-3.6889994 | 3.18E-02 | -9.58E+00 | -3.94E+00 | -1.45E+01 | -4.61E+00 |
| Nifursol | 4.42E-02 | -8.41E+00 | -3.78E+00 | -1.30E+01 | -3.81E+00 |
| Verbasoside Esi-6.7040005 | 3.39E-02 | -9.02E+00 | -3.84E+00 | -1.38E+01 | -4.21E+00 |
| Morroniside Esi-2.752999 | 5.73E-03 | -1.08E+01 | -4.93E+00 | -1.53E+01 | -6.33E+00 |
| PE(14:1(9Z)/18:4(6Z,9Z,12Z,15Z)) Esi-13.339001 | 1.01E-05 | 7.82E+00 | 6.31E+00 | 5.36E+00 | 1.03E+01 |
| 3-(3,4-Dihydroxyphenyl)lactate Esi-2.1679995 | 3.99E-02 | -8.76E+00 | -4.08E+00 | -1.33E+01 | -4.22E+00 |
| 2,3,4,5,6-Penta-O-acetyl-D-glucose Esi-2.6790004 | 3.52E-04 | -1.20E+01 | -6.35E+00 | -1.59E+01 | -8.16E+00 |
| Plumieride Esi-4.2519994 | 5.95E-03 | -1.03E+01 | -4.91E+00 | -1.46E+01 | -6.00E+00 |
| 1,2-Di-(9Z,12Z,15Z-octadecatrienoyl)-3-(Galactosyl-alpha-1-6-Galactosyl-beta-1)-glycerol Esi-17.001003 | 1.60E-02 | -1.04E+01 | -4.32E+00 | -1.53E+01 | -5.47E+00 |
| Gambierol Esi-13.5320015 | 3.64E-02 | 8.71E+00 | 3.90E+00 | 4.09E+00 | 1.33E+01 |
| Pubesenolide Esi-15.716002 | 7.63E-03 | -1.06E+01 | -5.63E+00 | -1.47E+01 | -6.61E+00 |
| 2-Hydroxybenzaldehyde O-[xylosyl-(1-6)-glucoside] Esi-3.7760007 | 3.34E-02 | -9.22E+00 | -3.97E+00 | -1.40E+01 | -4.44E+00 |
| Bruceantin Esi-7.031999 | 1.75E-02 | -9.42E+00 | -4.42E+00 | -1.38E+01 | -5.02E+00 |
| 3'-Keto-3'-deoxy-AMP Esi-12.677001 | 1.07E-02 | -9.05E+00 | -4.80E+00 | -1.30E+01 | -5.15E+00 |
| Artomunoxanthentrione epoxide Esi-2.2760005 | 2.70E-02 | -9.82E+00 | -4.14E+00 | -1.47E+01 | -4.93E+00 |
| Leonuridine Esi-2.2760005 | 2.81E-03 | -1.19E+01 | -5.42E+00 | -1.64E+01 | -7.40E+00 |
| Lucuminic acid Esi-4.8590007 | 1.62E-02 | -9.25E+00 | -4.30E+00 | -1.36E+01 | -4.86E+00 |
| Melleolide M | 9.57E-03 | -9.91E+00 | -4.63E+00 | -1.43E+01 | -5.53E+00 |
| Sudachiin A Esi-1.0020001 | 4.51E-02 | -7.93E+00 | -3.65E+00 | -1.24E+01 | -3.49E+00 |
| Narceinone Esi-6.5380015 | 3.47E-02 | -8.60E+00 | -3.81E+00 | -1.32E+01 | -3.98E+00 |
| Quercetin 3-beta-laminaribioside Esi-6.557999 | 1.52E-02 | -9.27E+00 | -4.69E+00 | -1.34E+01 | -5.15E+00 |
| Rugosinone | 1.86E-02 | -9.15E+00 | -4.26E+00 | -1.36E+01 | -4.75E+00 |
| Asperuloside tetraacetate | 3.30E-02 | -8.49E+00 | -4.13E+00 | -1.28E+01 | -4.21E+00 |
| alpha-Terthienyl | 3.45E-02 | -9.11E+00 | -3.81E+00 | -1.40E+01 | -4.22E+00 |
| 2,3,4,5,6-Penta-O-acetyl-D-glucose Esi-2.695 | 2.29E-03 | -1.08E+01 | -5.64E+00 | -1.48E+01 | -6.87E+00 |
| Morroniside Esi-2.306 | 3.30E-02 | -9.16E+00 | -4.11E+00 | -1.38E+01 | -4.52E+00 |
| Acetyl adenylate Esi-10.079 | 1.07E-02 | -9.71E+00 | -4.56E+00 | -1.41E+01 | -5.36E+00 |
| N-Methylcalystegine B2 Esi-2.139 | 3.50E-04 | -1.21E+01 | -6.47E+00 | -1.59E+01 | -8.26E+00 |
| 2-Amino-2-deoxyisochorismate Esi-3.794999 | 5.81E-03 | -1.01E+01 | -4.92E+00 | -1.43E+01 | -5.92E+00 |
| 4-Demethylsimmondsin 2'-(E)-ferulate | 3.08E-02 | -8.99E+00 | -4.04E+00 | -1.36E+01 | -4.41E+00 |
| Plumieride Esi-4.2519994 :1 | 1.96E-03 | -1.12E+01 | -5.52E+00 | -1.54E+01 | -7.08E+00 |
| trans-Caffeic acid [apiosyl-(1-6)-glucosyl] ester Esi-3.0089996 | 6.88E-03 | -1.02E+01 | -4.85E+00 | -1.44E+01 | -5.87E+00 |
| Volicitin | 4.44E-02 | -7.85E+00 | -4.01E+00 | -1.20E+01 | -3.71E+00 |
| GW 409544 Esi-5.527 | 1.27E-02 | -9.78E+00 | -4.59E+00 | -1.42E+01 | -5.40E+00 |
| (1R*,2R*,4R*,8S*)-p-Menthane-1,2,8,9-tetrol 9-glucoside Esi-7.543 | 2.70E-02 | -8.62E+00 | -4.02E+00 | -1.30E+01 | -4.24E+00 |
| Dihydroferulic acid 4-sulfate Esi-4.48 | 3.34E-02 | -8.34E+00 | -4.05E+00 | -1.26E+01 | -4.06E+00 |
| Linalool oxide D 3-[apiosyl-(1-6)-glucoside] Esi-8.4349985 | 3.32E-02 | -8.81E+00 | -3.89E+00 | -1.34E+01 | -4.18E+00 |
| Undecaprenyl phosphate mannose Esi-14.936997 | 2.83E-02 | 9.39E+00 | 4.10E+00 | 4.67E+00 | 1.41E+01 |
| Salicin 6-phosphate Esi-14.524001 | 3.35E-02 | -9.11E+00 | -3.91E+00 | -1.39E+01 | -4.32E+00 |
| 6-O-p-Coumaroyl-D-glucose Esi-4.6040015 | 3.34E-02 | -8.21E+00 | -4.19E+00 | -1.23E+01 | -4.09E+00 |
| Isodomoic acid A Esi-2.799 | 1.31E-02 | -1.00E+01 | -4.72E+00 | -1.44E+01 | -5.60E+00 |
| C24 H19 N12 | 1.26E-02 | -9.04E+00 | -4.47E+00 | -1.32E+01 | -4.91E+00 |
| Saxitoxin Esi-3.8219995 | 3.07E-02 | -9.79E+00 | -3.98E+00 | -1.48E+01 | -4.75E+00 |
| ?-lactic acid Esi-4.063001 | 2.76E-03 | -1.01E+01 | -5.97E+00 | -1.37E+01 | -6.59E+00 |
| PC(20:0/18:3(9Z,12Z,15Z)) | 5.16E-03 | -4.37E+00 | -4.30E+00 | -6.37E+00 | -2.36E+00 |
| Miglitol | 2.88E-03 | -1.07E+01 | -5.34E+00 | -1.48E+01 | -6.58E+00 |
| 6-Caffeoylsucrose | 3.34E-02 | -8.19E+00 | -4.35E+00 | -1.22E+01 | -4.17E+00 |
| 5-Methyl-THF Esi-9.696 | 4.27E-03 | -5.83E+00 | -4.56E+00 | -8.37E+00 | -3.28E+00 |
| C21 H33 N8 | 3.10E-02 | -8.42E+00 | -3.98E+00 | -1.28E+01 | -4.08E+00 |
| C22 H18 N14 | 5.01E-03 | -1.03E+01 | -5.91E+00 | -1.40E+01 | -6.58E+00 |
| CGP 28-392 Esi-2.3170002 | 3.44E-02 | -9.76E+00 | -3.81E+00 | -1.50E+01 | -4.53E+00 |
| Suspensolide F Esi-4.244001 | 2.86E-03 | -1.04E+01 | -5.43E+00 | -1.43E+01 | -6.45E+00 |
| Aloesin | 2.61E-05 | -1.16E+01 | -7.53E+00 | -1.47E+01 | -8.44E+00 |
| 2,4-Bis(acetamido)-2,4,6-trideoxy-beta-L-altropyranose Esi-3.238999 | 4.87E-02 | -8.45E+00 | -3.96E+00 | -1.30E+01 | -3.93E+00 |
| 4-Hydroxyphenylacetonitrile triacetylrhamnoside | 7.52E-03 | -9.95E+00 | -4.79E+00 | -1.42E+01 | -5.71E+00 |
| Methyl cellulose Esi-6.633002 | 1.42E-02 | -9.05E+00 | -4.39E+00 | -1.33E+01 | -4.84E+00 |
| Pelargonidin 3-(6-p-coumaroyl)glucoside | 7.71E-03 | -9.38E+00 | -4.90E+00 | -1.33E+01 | -5.44E+00 |
| Myricetin Esi-3.1220005 | 1.42E-02 | -9.19E+00 | -4.47E+00 | -1.34E+01 | -4.97E+00 |
| 7-Epiloganin tetraacetate Esi-7.293 | 2.39E-02 | -9.54E+00 | -4.12E+00 | -1.43E+01 | -4.80E+00 |
| Swertiamarin Esi-4.6540003 | 3.20E-02 | -9.54E+00 | -4.36E+00 | -1.42E+01 | -4.90E+00 |
| DG(20:5(5Z,8Z,11Z,14Z,17Z)/20:3(8Z,11Z,14Z)/0:0) Esi-15.845998 | 1.21E-02 | -9.02E+00 | -4.81E+00 | -1.29E+01 | -5.12E+00 |
| C23 H18 N20 O | 3.82E-02 | -7.89E+00 | -4.13E+00 | -1.19E+01 | -3.84E+00 |
| Plumieride Esi-3.7060006 | 6.33E-03 | -1.04E+01 | -4.91E+00 | -1.48E+01 | -6.09E+00 |
| PE(20:2(11Z,14Z)/16:0) | 1.75E-02 | -5.82E+00 | -3.89E+00 | -8.79E+00 | -2.84E+00 |
| (S)-Tetrahydropalmatine | 8.31E-03 | -9.64E+00 | -4.70E+00 | -1.38E+01 | -5.45E+00 |
| C11 H12 Cl N3 O4 | 1.55E-02 | -9.23E+00 | -4.50E+00 | -1.35E+01 | -5.00E+00 |
| 1'-Acetoxyeugenol acetate Esi-7.14 | 3.66E-02 | -8.20E+00 | -3.90E+00 | -1.26E+01 | -3.85E+00 |
| Citronellyl beta-sophoroside Esi-7.0249996 | 3.32E-02 | -9.42E+00 | -4.01E+00 | -1.43E+01 | -4.57E+00 |
| Furaneol 4-(6-malonylglucoside) Esi-3.055 | 1.10E-02 | -1.05E+01 | -4.72E+00 | -1.51E+01 | -5.92E+00 |
| Patellamide C Esi-4.3519998 | 3.63E-02 | -5.26E+00 | -3.79E+00 | -8.10E+00 | -2.42E+00 |
| Catechin 3',7-diglucoside Esi-6.14 | 4.01E-04 | -1.25E+01 | -6.35E+00 | -1.65E+01 | -8.46E+00 |
| 7,8-Dihydrovomifoliol 9-[apiosyl-(1-6)-glucoside] Esi-6.878999 | 1.61E-02 | -9.49E+00 | -4.49E+00 | -1.39E+01 | -5.12E+00 |
| Temefos | 3.34E-02 | -8.33E+00 | -3.93E+00 | -1.27E+01 | -3.97E+00 |
| Apiosylglucosyl 4-hydroxybenzoate Esi-2.2239997 | 3.34E-02 | -8.96E+00 | -4.36E+00 | -1.33E+01 | -4.58E+00 |
| Decarbamoylgonyautoxin 1 | 3.41E-02 | -8.73E+00 | -3.82E+00 | -1.34E+01 | -4.06E+00 |
| Adenosine | 3.47E-03 | -4.96E+00 | -4.59E+00 | -7.10E+00 | -2.81E+00 |
| Sulbactam | 2.96E-02 | -8.95E+00 | -4.01E+00 | -1.35E+01 | -4.38E+00 |
| 6''-Acetylliquiritin Esi-2.8069994 | 3.34E-02 | -8.07E+00 | -4.36E+00 | -1.20E+01 | -4.13E+00 |
| Citronellyl beta-sophoroside Esi-9.099999 | 3.74E-02 | -8.40E+00 | -3.80E+00 | -1.29E+01 | -3.86E+00 |
| Bruceoside A Esi-6.380999 | 3.25E-02 | -8.94E+00 | -3.99E+00 | -1.35E+01 | -4.33E+00 |
| Acuminoside Esi-8.9609995 | 3.14E-02 | -8.97E+00 | -3.94E+00 | -1.36E+01 | -4.32E+00 |
| 6'-Methoxypolygoacetophenoside Esi-4.6139994 | 1.59E-02 | -9.09E+00 | -4.39E+00 | -1.33E+01 | -4.84E+00 |
| Myriocin Esi-11.968998 | 5.08E-03 | -9.58E+00 | -5.00E+00 | -1.35E+01 | -5.67E+00 |
| Piperonyl sulfoxide Esi-3.4869998 | 3.44E-02 | -8.39E+00 | -4.01E+00 | -1.28E+01 | -4.04E+00 |
| C23 H34 O16 Esi-2.812 | 2.76E-03 | -1.11E+01 | -6.56E+00 | -1.47E+01 | -7.51E+00 |
| C18 H20 N14 O3 | 7.52E-03 | -9.80E+00 | -5.68E+00 | -1.35E+01 | -6.12E+00 |
| dTDP / Thymidine 5'-diphosphate Esi-6.194 | 5.78E-03 | -1.11E+01 | -5.77E+00 | -1.51E+01 | -6.99E+00 |
| Phrymarolin I Esi-3.2309992 | 2.98E-03 | -1.03E+01 | -5.33E+00 | -1.42E+01 | -6.32E+00 |
| 6S,9R-Dihydroxy-4,7E-megastigmadien-3-one 9-[apiosyl-(1-6)-glucoside] Esi-5.279 | 1.24E-02 | -1.01E+01 | -4.79E+00 | -1.45E+01 | -5.74E+00 |
| 1-(beta-D-Glucopyranosyloxy)-3-octanone Esi-5.001 | 1.76E-02 | -9.62E+00 | -4.89E+00 | -1.38E+01 | -5.43E+00 |
| Melledonal C | 4.03E-03 | -1.07E+01 | -5.45E+00 | -1.47E+01 | -6.61E+00 |
| PE(20:5(5Z,8Z,11Z,14Z,17Z)/24:1(15Z)) | 1.09E-05 | -8.16E+00 | -6.28E+00 | -1.07E+01 | -5.58E+00 |
| beta-Hydroxyacteoside Esi-6.5019994 | 1.50E-02 | -9.29E+00 | -4.41E+00 | -1.36E+01 | -4.97E+00 |
| Irisolidone 7-O-glucuronide | 3.34E-02 | -8.24E+00 | -4.36E+00 | -1.23E+01 | -4.21E+00 |
| N-Acetyl-L-phenylalanine Esi-5.4380016 | 7.71E-03 | -1.03E+01 | -5.52E+00 | -1.42E+01 | -6.33E+00 |
| PE(20:0/dm18:0) Esi-15.760003 | 1.36E-04 | 6.76E+00 | 5.73E+00 | 4.41E+00 | 9.11E+00 |
| Oxyapramycin Esi-7.4459996 | 1.33E-02 | -9.03E+00 | -4.62E+00 | -1.31E+01 | -4.99E+00 |
| C17 H12 N9 O4 | 1.07E-02 | -1.03E+01 | -4.65E+00 | -1.49E+01 | -5.77E+00 |
| C14 H18 N9 O3 | 3.34E-02 | -8.93E+00 | -4.37E+00 | -1.33E+01 | -4.57E+00 |
| Myriocin Esi-11.8099985 | 2.27E-02 | -8.47E+00 | -4.16E+00 | -1.26E+01 | -4.29E+00 |
| Isodonal Esi-5.0169992 | 3.57E-02 | -8.56E+00 | -3.79E+00 | -1.32E+01 | -3.94E+00 |
| Undecaprenyl phosphate mannose Esi-15.106002 | 3.60E-02 | 8.57E+00 | 3.93E+00 | 4.05E+00 | 1.31E+01 |
| Sudachiin A Esi-0.8809998 | 9.38E-03 | -9.22E+00 | -4.74E+00 | -1.32E+01 | -5.22E+00 |
| Cytidine diphosphate choline (CDPcholine) | 1.97E-03 | -1.14E+01 | -6.64E+00 | -1.50E+01 | -7.73E+00 |
| Nigakilactone B Esi-14.852 | 1.99E-02 | 9.25E+00 | 4.17E+00 | 4.72E+00 | 1.38E+01 |
| beta-D-4-Deoxy-delta4-GlcA-(1-4)-beta-D-Glc-(1-4)-alpha-L-Rha-(1-3)-beta-D-Glc Esi-1.226 | 1.40E-02 | -8.97E+00 | -4.46E+00 | -1.31E+01 | -4.85E+00 |
| 1-Palmitoyl Lysophosphatidic Acid Esi-13.829999 | 2.96E-03 | -1.15E+01 | -5.41E+00 | -1.58E+01 | -7.11E+00 |
| 8-Acetoxy-4'-methoxypinoresinol 4-glucoside Esi-6.810999 | 3.14E-02 | -9.40E+00 | -4.04E+00 | -1.42E+01 | -4.60E+00 |
| Isonocardicin C Esi-5.111 | 2.01E-03 | -1.05E+01 | -5.71E+00 | -1.43E+01 | -6.71E+00 |
| Demethyloleuropein Esi-5.0120006 | 1.34E-02 | -9.01E+00 | -4.49E+00 | -1.31E+01 | -4.90E+00 |
| 2,3-Butanediol apiosylglucoside Esi-3.0909998 | 1.35E-02 | -9.69E+00 | -4.69E+00 | -1.40E+01 | -5.40E+00 |
| C10 H2 Cl3 O S5 | 4.24E-02 | -7.55E+00 | -3.83E+00 | -1.16E+01 | -3.46E+00 |
| Kanamycin A 3'-phosphate Esi-6.283 | 1.93E-02 | -9.10E+00 | -4.36E+00 | -1.34E+01 | -4.79E+00 |
| PE(15:0/20:0) Esi-13.035003 | 9.31E-03 | 7.37E+00 | 4.19E+00 | 3.87E+00 | 1.09E+01 |
| C24 H24 N17 O3 | 3.65E-02 | -8.30E+00 | -3.88E+00 | -1.27E+01 | -3.88E+00 |
| PE(14:1(9Z)/22:0) | 7.62E-03 | -5.58E+00 | -4.31E+00 | -8.15E+00 | -3.00E+00 |
| Paeonolide Esi-0.8240001 | 3.34E-03 | -9.17E+00 | -5.24E+00 | -1.27E+01 | -5.59E+00 |
| Cinncassiol D2 glucoside Esi-7.7839985 | 3.58E-02 | -8.22E+00 | -3.82E+00 | -1.26E+01 | -3.81E+00 |
| Dutasteride Esi-8.130001 | 4.54E-02 | -7.87E+00 | -3.68E+00 | -1.22E+01 | -3.49E+00 |
| Morroniside Esi-2.8499994 | 3.17E-03 | -1.10E+01 | -5.39E+00 | -1.52E+01 | -6.80E+00 |
| Chalcomoracin Esi-4.5789995 | 3.34E-02 | -8.41E+00 | -4.37E+00 | -1.25E+01 | -4.30E+00 |
| Methyl-2-alpha-L-fucopyranosyl-beta-D-galactoside Esi-2.6850004 | 3.88E-02 | -9.13E+00 | -3.73E+00 | -1.41E+01 | -4.13E+00 |
| 8-Hydroxyhesperetin 7-[6-acetylglucosyl-(1-2)-glucoside] Esi-7.8639994 | 1.65E-02 | -9.03E+00 | -4.95E+00 | -1.29E+01 | -5.14E+00 |
| beta-Hydroxyacteoside Esi-6.486 | 3.76E-03 | -1.03E+01 | -5.16E+00 | -1.44E+01 | -6.23E+00 |
| Asperuloside tetraacetate Esi-5.6459985 | 1.43E-02 | -9.08E+00 | -4.39E+00 | -1.33E+01 | -4.85E+00 |
| 12-nitro-9Z,12Z-octadecadienoic acid Esi-8.516001 | 4.36E-02 | -8.06E+00 | -3.71E+00 | -1.25E+01 | -3.60E+00 |
| Nafenopin glucuronide Esi-4.654 | 1.14E-02 | -9.48E+00 | -4.53E+00 | -1.38E+01 | -5.20E+00 |
| Garcimangosone D Esi-3.999 | 3.14E-02 | -8.74E+00 | -3.94E+00 | -1.33E+01 | -4.21E+00 |
| alpha-Viniferin | 1.43E-02 | -1.04E+01 | -4.48E+00 | -1.52E+01 | -5.63E+00 |
| Gentiobiosyl 2-methyl-6-oxo-2E,4E-heptadienoate Esi-5.3320003 | 2.75E-02 | -8.87E+00 | -4.01E+00 | -1.34E+01 | -4.35E+00 |
| (14alpha,17beta,20S,22R)-14,20-Epoxy-17-hydroxy-1-oxowitha-3,5,24-trienolide Esi-6.8520007 | 2.77E-03 | -1.09E+01 | -6.37E+00 | -1.46E+01 | -7.29E+00 |
| Tetrahydroaldosterone-3-glucuronide Esi-8.447999 | 2.92E-02 | -8.61E+00 | -3.98E+00 | -1.30E+01 | -4.19E+00 |
| trans-Caffeic acid [apiosyl-(1-6)-glucosyl] ester Esi-3.097 | 2.37E-02 | -9.04E+00 | -4.09E+00 | -1.36E+01 | -4.52E+00 |
| Neoacrimarine H Esi-2.7999995 | 3.41E-02 | -8.21E+00 | -3.98E+00 | -1.25E+01 | -3.93E+00 |
| Sesamolinol 4'-O-b-D-glucosyl (1-6)-O-b-D-glucoside Esi-1.0389998 | 1.34E-02 | -9.07E+00 | -4.43E+00 | -1.32E+01 | -4.89E+00 |
| Shoyuflavone B Esi-5.0670004 | 7.08E-08 | -1.32E+01 | -1.02E+01 | -1.59E+01 | -1.06E+01 |
| Fluocinolone Esi-14.923003 | 4.82E-02 | 5.22E+00 | 3.75E+00 | 2.33E+00 | 8.11E+00 |
| Ipolamiide Esi-3.021 | 8.40E-04 | -1.14E+01 | -6.10E+00 | -1.53E+01 | -7.59E+00 |
| Mollicellin C | 3.14E-02 | -8.58E+00 | -4.18E+00 | -1.29E+01 | -4.30E+00 |
| Artonin R Esi-5.265 | 1.95E-02 | -9.02E+00 | -4.23E+00 | -1.34E+01 | -4.65E+00 |
| (12S,15S)-15-O-Demethyl-10,29-dideoxy-11,12-dihydro-striatin C Esi-12.670999 | 3.34E-02 | -7.73E+00 | -4.06E+00 | -1.17E+01 | -3.77E+00 |
| Austalide C Esi-6.62 | 3.34E-02 | -7.98E+00 | -4.37E+00 | -1.19E+01 | -4.09E+00 |
| 2'-Oxoaloesol 7-glucoside Esi-14.173998 | 1.20E-05 | -1.14E+01 | -7.88E+00 | -1.44E+01 | -8.45E+00 |
| CMP-N-acetylneuraminic acid Esi-7.849999 | 3.14E-02 | -8.52E+00 | -4.03E+00 | -1.29E+01 | -4.17E+00 |
| Neoacrimarine G Esi-5.6790004 | 2.14E-04 | -1.17E+01 | -7.84E+00 | -1.49E+01 | -8.59E+00 |
| Pyranocyanin B Esi-6.247001 | 2.53E-02 | -8.73E+00 | -4.17E+00 | -1.30E+01 | -4.42E+00 |
| Morroniside Esi-2.643 | 2.83E-02 | -9.47E+00 | -4.11E+00 | -1.42E+01 | -4.71E+00 |
| Loquatoside Esi-7.221 | 2.13E-02 | -8.47E+00 | -4.33E+00 | -1.25E+01 | -4.42E+00 |
| 1,2,10-Trihydroxydihydro-trans-linalyl oxide 7-O-beta-D-glucopyranoside Esi-5.622001 | 1.86E-02 | -9.00E+00 | -4.21E+00 | -1.34E+01 | -4.64E+00 |
| CE(22:6(4Z,7Z,10Z,13Z,16Z,19Z)) Esi-16.519001 | 3.02E-02 | 8.97E+00 | 4.01E+00 | 4.38E+00 | 1.36E+01 |
| C22 H22 N18 O5 | 2.31E-02 | -8.69E+00 | -4.14E+00 | -1.30E+01 | -4.39E+00 |
| PE(20:4(8Z,11Z,14Z,17Z)/22:0) Esi-15.847996 | 3.58E-03 | 5.64E+00 | 4.60E+00 | 3.20E+00 | 8.08E+00 |
| C16 H18 N7 O5 | 3.63E-02 | -8.91E+00 | -3.88E+00 | -1.36E+01 | -4.17E+00 |
| Destomysin Esi-4.8450003 | 3.34E-02 | -8.46E+00 | -4.35E+00 | -1.26E+01 | -4.32E+00 |
| C56 H80 N3 O5 Esi-16.992002 | 3.73E-03 | -1.05E+01 | -5.53E+00 | -1.45E+01 | -6.56E+00 |
| O-Demethylpuromycin Esi-6.4110007 | 1.62E-02 | -9.63E+00 | -4.98E+00 | -1.38E+01 | -5.50E+00 |
| 24-Hydroxyglycyrrhetic acid Esi-14.898995 | 4.56E-02 | 7.88E+00 | 3.76E+00 | 3.54E+00 | 1.22E+01 |
| Bruceantin Esi-6.718 | 1.61E-02 | -9.50E+00 | -4.46E+00 | -1.39E+01 | -5.10E+00 |
| Deoxyloganic acid tetraacetate Esi-5.676 | 1.59E-02 | -8.75E+00 | -4.36E+00 | -1.29E+01 | -4.64E+00 |
| C9 H20 Cl2 N8 O3 S3 | 7.62E-03 | -1.04E+01 | -5.65E+00 | -1.44E+01 | -6.49E+00 |
| Herbimycin Esi-5.2960005 | 3.15E-02 | -8.64E+00 | -4.01E+00 | -1.31E+01 | -4.21E+00 |
| PE(14:1(9Z)/14:1(9Z)) Esi-12.653 | 4.46E-04 | -6.49E+00 | -5.45E+00 | -8.87E+00 | -4.11E+00 |
| C18 H40 N2 O2 S3 | 4.99E-02 | -7.49E+00 | -3.69E+00 | -1.17E+01 | -3.30E+00 |
| Deutzioside pentaacetate Esi-6.8440013 | 1.62E-02 | -9.02E+00 | -4.97E+00 | -1.29E+01 | -5.15E+00 |
| Deutzioside pentaacetate Esi-6.241 | 7.52E-03 | -1.01E+01 | -5.67E+00 | -1.40E+01 | -6.33E+00 |
| > limit Esi-16.267 | 1.86E-02 | 8.16E+00 | 4.26E+00 | 4.23E+00 | 1.21E+01 |
| Bruceantin Esi-6.483001 | 1.61E-02 | -9.65E+00 | -4.98E+00 | -1.38E+01 | -5.52E+00 |
| Tetrahydrofolic acid Esi-7.588999 | 5.01E-16 | -8.03E+00 | -1.06E+01 | -9.54E+00 | -6.53E+00 |
| > limit Esi-14.821007 :1 | 1.42E-02 | -5.50E+00 | -4.77E+00 | -7.91E+00 | -3.09E+00 |
| Calophyllolide Esi-14.509002 | 3.49E-02 | -8.20E+00 | -3.85E+00 | -1.26E+01 | -3.83E+00 |
| Cinncassiol D2 glucoside Esi-8.194 | 8.28E-03 | -9.16E+00 | -5.56E+00 | -1.27E+01 | -5.65E+00 |
| Tetrahydroaldosterone-3-glucuronide Esi-8.441001 | 3.34E-02 | -8.01E+00 | -4.36E+00 | -1.19E+01 | -4.10E+00 |
| Cercosporin Esi-3.3449998 | 3.34E-02 | -7.68E+00 | -4.36E+00 | -1.14E+01 | -3.93E+00 |
| Cappariloside B Esi-4.600999 | 6.70E-03 | -1.07E+01 | -4.86E+00 | -1.52E+01 | -6.19E+00 |
| Glucoliquiritin apioside Esi-1.047 | 1.18E-02 | -8.88E+00 | -4.58E+00 | -1.29E+01 | -4.90E+00 |
| Hydroxysafflor yellow A Esi-4.7900004 | 3.40E-02 | -9.04E+00 | -3.90E+00 | -1.38E+01 | -4.27E+00 |
| Ipecoside Esi-5.475999 | 2.14E-02 | -1.03E+01 | -4.16E+00 | -1.54E+01 | -5.25E+00 |
| 1-Hydroxy-3,6,7-trimethoxy-2-(3-methyl-2-butenyl)-8-(2-hydroxy-3-methyl-3-butenyl)-xanthone Esi-14.835002 | 3.14E-02 | -8.16E+00 | -4.17E+00 | -1.22E+01 | -4.09E+00 |
| O-1,4-?-L-Dihydrostreptosyl-streptidine 6-phosphate | 4.64E-02 | -7.63E+00 | -3.76E+00 | -1.18E+01 | -3.43E+00 |
| PE(20:3(8Z,11Z,14Z)/18:3(6Z,9Z,12Z)) | 3.24E-06 | -8.01E+00 | -6.66E+00 | -1.04E+01 | -5.61E+00 |
| Curacin A Esi-7.908999 | 4.06E-02 | -7.60E+00 | -3.74E+00 | -1.18E+01 | -3.43E+00 |
| PE(24:0/20:5(5Z,8Z,11Z,14Z,17Z)) Esi-14.932002 | 3.52E-03 | -5.66E+00 | -4.61E+00 | -8.10E+00 | -3.22E+00 |
| Landomycin D Esi-6.171 | 1.48E-02 | -8.72E+00 | -4.62E+00 | -1.26E+01 | -4.80E+00 |
| C20 H14 Cl O2 | 1.55E-02 | -9.78E+00 | -4.34E+00 | -1.44E+01 | -5.18E+00 |
| 1'-Hydroxy-?-carotene glucoside Esi-16.073 | 4.21E-02 | -8.05E+00 | -3.87E+00 | -1.24E+01 | -3.72E+00 |
| GSSG / L-Gluthathione (oxidized) | 4.75E-02 | -3.65E+00 | -3.42E+00 | -5.78E+00 | -1.52E+00 |
| > limit Esi-14.828001 | 1.84E-02 | -5.04E+00 | -4.56E+00 | -7.36E+00 | -2.73E+00 |
| PE(20:5(5Z,8Z,11Z,14Z,17Z)/24:1(15Z)) Esi-15.779001 | 1.09E-02 | -4.85E+00 | -4.14E+00 | -7.17E+00 | -2.52E+00 |
| > limit Esi-6.078001 | 3.49E-02 | -4.80E+00 | -3.79E+00 | -7.38E+00 | -2.22E+00 |
| PE(20:0/dm18:0) Esi-16.754 | 8.20E-06 | -7.56E+00 | -6.43E+00 | -9.90E+00 | -5.22E+00 |
| Artabsinolide A | 1.93E-02 | 9.65E+00 | 4.39E+00 | 5.10E+00 | 1.42E+01 |
| 3S-hydroxy-octanoyl-CoA Esi-14.324 | 1.65E-03 | 6.24E+00 | 5.20E+00 | 3.82E+00 | 8.65E+00 |
| C18 H47 Cl N10 O S3 | 9.38E-03 | 1.16E+01 | 4.78E+00 | 6.59E+00 | 1.66E+01 |
| (8Z,11Z,14Z,17Z)-Icosatetraenoyl-CoA Esi-13.984 | 1.66E-02 | 5.94E+00 | 4.26E+00 | 3.10E+00 | 8.79E+00 |
| Withaperuvin B Esi-12.697999 | 1.59E-02 | 8.91E+00 | 4.36E+00 | 4.72E+00 | 1.31E+01 |
| C22 H34 O4 Esi-12.546998 | 7.98E-03 | 1.02E+01 | 4.82E+00 | 5.87E+00 | 1.46E+01 |
| Uridine 5'-diphosphoglucuronic acid | 2.67E-02 | 3.81E+00 | 3.64E+00 | 1.75E+00 | 5.88E+00 |
| Valtratum | 7.57E-03 | 1.10E+01 | 5.66E+00 | 6.89E+00 | 1.52E+01 |
| Uridine 5'-diphosphoglucuronic acid Esi-0.89 | 8.28E-05 | 5.60E+00 | 5.60E+00 | 3.62E+00 | 7.58E+00 |
| Valtratum Esi-12.533 | 7.84E-03 | 1.06E+01 | 5.60E+00 | 6.58E+00 | 1.47E+01 |
| PE(22:6(4Z,7Z,10Z,13Z,16Z,19Z)/22:4(7Z,10Z,13Z,16Z)) | 1.66E-07 | -9.38E+00 | -7.33E+00 | -1.19E+01 | -6.84E+00 |
| N-desmethylimatinib Esi-13.561997 | 3.34E-02 | -8.77E+00 | -4.08E+00 | -1.32E+01 | -4.30E+00 |
| Saquinavir Esi-16.208998 | 1.88E-02 | 6.51E+00 | 4.24E+00 | 3.37E+00 | 9.66E+00 |
| PIP(16:1(9Z)/16:1(9Z)) | 1.68E-02 | 5.85E+00 | 4.25E+00 | 3.05E+00 | 8.66E+00 |
| PE(20:2(11Z,14Z)/20:2(11Z,14Z)) | 6.33E-03 | 4.89E+00 | 4.59E+00 | 2.75E+00 | 7.04E+00 |
| PE(22:4(7Z,10Z,13Z,16Z)/24:0) Esi-13.719001 | 7.83E-03 | 5.29E+00 | 4.30E+00 | 2.84E+00 | 7.74E+00 |
| Rhodojaponin I Esi-12.712998 | 3.50E-02 | 8.60E+00 | 3.80E+00 | 3.97E+00 | 1.32E+01 |
| Grayanotoxin I Esi-14.937999 | 3.50E-02 | 8.32E+00 | 3.82E+00 | 3.85E+00 | 1.28E+01 |
| PE(20:2(11Z,14Z)/20:2(11Z,14Z)) Esi-3.264 | 6.49E-03 | 4.79E+00 | 4.58E+00 | 2.68E+00 | 6.89E+00 |
| Uridine 5'-diphosphoglucuronic acid Esi-1.0959998 | 3.33E-02 | 3.34E+00 | 3.53E+00 | 1.47E+00 | 5.20E+00 |
| Acrovestone Esi-15.186997 | 3.33E-02 | 8.46E+00 | 4.08E+00 | 4.15E+00 | 1.28E+01 |
| 3S-hydroxy-octanoyl-CoA Esi-14.512999 | 1.23E-05 | 5.93E+00 | 6.34E+00 | 4.07E+00 | 7.79E+00 |
| Pipercyclobutanamide A Esi-13.802001 | 3.34E-02 | 8.73E+00 | 3.88E+00 | 4.12E+00 | 1.33E+01 |
| Telmisartan | 3.35E-02 | 9.28E+00 | 4.34E+00 | 4.72E+00 | 1.38E+01 |
| Corchoionol C 9-glucoside Esi-9.175001 | 3.44E-02 | 8.21E+00 | 3.97E+00 | 3.92E+00 | 1.25E+01 |
| PE(18:4(6Z,9Z,12Z,15Z)/22:6(4Z,7Z,10Z,13Z,16Z,19Z)) Esi-14.485995 | 4.93E-02 | 4.58E+00 | 3.36E+00 | 1.87E+00 | 7.29E+00 |
| PE(22:5(4Z,7Z,10Z,13Z,16Z)/20:3(5Z,8Z,11Z)) Esi-16.972 | 2.14E-02 | -5.07E+00 | -3.80E+00 | -7.72E+00 | -2.42E+00 |
| Tetrahydrofolic acid Esi-14.392999 | 1.09E-03 | 3.44E+00 | 4.86E+00 | 2.04E+00 | 4.83E+00 |
| Withaperuvin F Esi-14.871001 | 1.53E-02 | 8.99E+00 | 4.55E+00 | 4.90E+00 | 1.31E+01 |
| Uridine 5'-diphosphoglucuronic acid Esi-1.269 | 3.34E-02 | 3.11E+00 | 3.53E+00 | 1.37E+00 | 4.84E+00 |
| PE(22:2(13Z,16Z)/22:6(4Z,7Z,10Z,13Z,16Z,19Z)) Esi-16.509998 | 3.34E-03 | -6.61E+00 | -4.60E+00 | -9.47E+00 | -3.76E+00 |
| Propinol adenylate Esi-4.214 | 3.40E-02 | 9.84E+00 | 4.31E+00 | 4.98E+00 | 1.47E+01 |
| PE(20:0/dm18:0) Esi-14.9400015 | 7.55E-03 | 6.11E+00 | 4.32E+00 | 3.29E+00 | 8.92E+00 |
| PE(15:0/22:6(4Z,7Z,10Z,13Z,16Z,19Z)) Esi-16.442 | 1.68E-03 | 5.74E+00 | 5.06E+00 | 3.47E+00 | 8.01E+00 |
| (8Z,11Z,14Z,17Z)-Icosatetraenoyl-CoA Esi-14.495 | 3.80E-02 | 4.65E+00 | 3.73E+00 | 2.10E+00 | 7.19E+00 |
| PE(15:0/22:5(4Z,7Z,10Z,13Z,16Z)) Esi-16.338 | 8.05E-03 | 5.49E+00 | 4.26E+00 | 2.93E+00 | 8.05E+00 |
| Undecaprenyl phosphate mannose Esi-15.316 | 1.70E-02 | 9.32E+00 | 4.92E+00 | 5.28E+00 | 1.34E+01 |
| L-2-Aminoadipate adenylate Esi-13.192997 | 3.81E-02 | -5.80E+00 | -3.89E+00 | -8.90E+00 | -2.71E+00 |
| PI(16:0/22:3(10Z,13Z,16Z)) Esi-15.289999 | 3.76E-02 | 4.50E+00 | 3.73E+00 | 2.04E+00 | 6.96E+00 |
| PE(18:4(6Z,9Z,12Z,15Z)/22:6(4Z,7Z,10Z,13Z,16Z,19Z)) Esi-14.345997 | 3.47E-02 | 4.82E+00 | 3.53E+00 | 2.11E+00 | 7.53E+00 |
| PI(22:6(4Z,7Z,10Z,13Z,16Z,19Z)/18:0) Esi-15.561997 | 3.27E-02 | 5.97E+00 | 3.70E+00 | 2.74E+00 | 9.21E+00 |
| GSSG / L-Gluthathione (oxidized) Esi-14.804 | 2.17E-02 | 3.39E+00 | 3.94E+00 | 1.66E+00 | 5.11E+00 |
| Mecarbam | 2.86E-03 | -1.34E+01 | -6.49E+00 | -1.78E+01 | -8.99E+00 |
| Dihydrolipoamide | 5.00E-07 | -1.32E+01 | -8.23E+00 | -1.65E+01 | -9.98E+00 |
| L-Iduronate 2-sulfate | 2.81E-03 | -1.35E+01 | -6.51E+00 | -1.79E+01 | -9.06E+00 |
| 2-Mercaptobenzothiazole | 8.31E-03 | -1.12E+01 | -5.55E+00 | -1.55E+01 | -6.91E+00 |
| Clitocine | 2.77E-03 | -1.30E+01 | -6.53E+00 | -1.73E+01 | -8.77E+00 |
| 4-Bromophenylacetate | 2.69E-03 | -1.34E+01 | -6.58E+00 | -1.78E+01 | -9.08E+00 |
| C6 H N O8 S2 | 7.80E-03 | -1.15E+01 | -5.61E+00 | -1.59E+01 | -7.15E+00 |
| 2,3-Dihydro-2-S-glutathionyl-3-hydroxy bromobenzene | 7.62E-03 | -1.25E+01 | -5.64E+00 | -1.72E+01 | -7.78E+00 |
| C28 H18 N20 O6 | 3.23E-02 | -9.67E+00 | -4.18E+00 | -1.45E+01 | -4.84E+00 |
| 4-Bromophenylacetate Esi-4.7179995 | 2.76E-03 | -1.26E+01 | -6.54E+00 | -1.67E+01 | -8.49E+00 |
| Amidithion Esi-12.791 | 2.88E-03 | -1.26E+01 | -6.47E+00 | -1.67E+01 | -8.44E+00 |
| C17 H13 N7 O4 S2 | 2.81E-03 | -1.23E+01 | -6.51E+00 | -1.63E+01 | -8.28E+00 |
| Famotidine | 3.35E-02 | -9.03E+00 | -4.33E+00 | -1.35E+01 | -4.59E+00 |
| Amidithion Esi-12.554 | 2.98E-03 | -1.16E+01 | -6.44E+00 | -1.54E+01 | -7.74E+00 |
| 3'-Keto-3'-deoxy-AMP Esi-10.9 | 7.71E-03 | -1.11E+01 | -5.62E+00 | -1.53E+01 | -6.86E+00 |
| Glucocapparin | 1.70E-02 | -1.01E+01 | -4.92E+00 | -1.44E+01 | -5.71E+00 |
| dCDP / 2'-Deoxycytidine 5'-diphosphate | 7.52E-03 | -1.18E+01 | -5.67E+00 | -1.63E+01 | -7.39E+00 |
| C12 H5 N7 O2 S2 | 7.57E-03 | -1.12E+01 | -5.66E+00 | -1.54E+01 | -6.99E+00 |
| 2-Methyl-4-amino-5-hydroxymethylpyrimidine diphosphate | 3.39E-02 | -9.24E+00 | -4.31E+00 | -1.38E+01 | -4.68E+00 |
| Dihydro-4,6-dimethyl-2-(1-methylethyl)-4H-1,3,5-dithiazine | 2.48E-03 | -1.32E+01 | -6.66E+00 | -1.74E+01 | -8.95E+00 |
| PE(20:2(11Z,14Z)/14:0) Esi-16.048998 | 4.61E-04 | -7.21E+00 | -5.44E+00 | -9.86E+00 | -4.56E+00 |
| Luteoforol Esi-13.218001 | 1.65E-02 | -8.82E+00 | -4.32E+00 | -1.30E+01 | -4.65E+00 |
| (2'S)-Deoxymyxol 2'-(2,4-di-O-methyl-?-L-fucoside) Esi-16.996 | 3.40E-02 | -8.55E+00 | -4.03E+00 | -1.30E+01 | -4.14E+00 |
| Ononin Esi-2.7830005 | 1.24E-02 | -9.79E+00 | -4.77E+00 | -1.41E+01 | -5.52E+00 |
| 2-Mercaptobenzothiazole Esi-9.344001 | 3.34E-02 | -8.43E+00 | -4.35E+00 | -1.26E+01 | -4.30E+00 |
| Digallate Esi-9.904 | 1.65E-02 | -9.52E+00 | -4.96E+00 | -1.36E+01 | -5.43E+00 |
| 3'-Keto-3'-deoxy-AMP Esi-12.634002 | 3.34E-02 | -9.65E+00 | -4.37E+00 | -1.44E+01 | -4.94E+00 |
| Thiodiacetic acid | 2.42E-03 | -1.28E+01 | -6.69E+00 | -1.69E+01 | -8.74E+00 |
| Cinncassiol C1 19-glucoside Esi-6.754999 | 3.07E-02 | -9.00E+00 | -3.97E+00 | -1.36E+01 | -4.36E+00 |
| EPTC | 2.48E-03 | -1.21E+01 | -6.65E+00 | -1.60E+01 | -8.23E+00 |
| Cinnamtannin D2 | 4.63E-03 | -9.95E+00 | -5.26E+00 | -1.39E+01 | -6.05E+00 |
| 2-Mercaptobenzothiazole Esi-9.904 | 2.51E-03 | -1.12E+01 | -6.64E+00 | -1.48E+01 | -7.62E+00 |
| L-gamma-glutamyl-L-isoleucine Esi-3.0820005 | 1.43E-02 | -9.42E+00 | -4.41E+00 | -1.38E+01 | -5.05E+00 |
| 3'-Keto-3'-deoxy-AMP Esi-12.854003 | 3.34E-02 | -8.85E+00 | -4.36E+00 | -1.32E+01 | -4.52E+00 |
| C15 H7 N3 O13 | 1.64E-02 | -9.74E+00 | -4.96E+00 | -1.39E+01 | -5.56E+00 |
| PE(20:1(11Z)/18:3(6Z,9Z,12Z)) | 3.69E-02 | -4.36E+00 | -3.49E+00 | -6.85E+00 | -1.88E+00 |
| (R)-3,4-Dihydro-2-methyl-2-(4,8,12-trimethyl-3,7,11-tridecatrienyl)-2H-1-benzopyran-6-ol Esi-15.888 | 3.34E-02 | -8.27E+00 | -4.37E+00 | -1.23E+01 | -4.23E+00 |
| PI(16:0/18:0) Esi-14.615998 | 2.03E-03 | -7.43E+00 | -5.07E+00 | -1.04E+01 | -4.49E+00 |
| Uscharidin Esi-15.989 | 3.97E-02 | 8.72E+00 | 4.16E+00 | 4.26E+00 | 1.32E+01 |
| <none> Esi-0.83600014 | 4.08E-02 | 7.92E+00 | 3.82E+00 | 3.63E+00 | 1.22E+01 |
| LPA(0:0/16:0) Esi-9.822 | 8.87E-04 | 6.15E+00 | 5.03E+00 | 3.73E+00 | 8.57E+00 |
| Estrone 3-sulfate | 1.18E-02 | 5.18E+00 | 4.17E+00 | 2.70E+00 | 7.67E+00 |
| PE(15:0/P-16:0) Esi-16.992998 | 3.48E-02 | 3.86E+00 | 3.54E+00 | 1.69E+00 | 6.02E+00 |
| PE(15:0/18:0) Esi-16.937 | 2.25E-02 | 3.60E+00 | 3.93E+00 | 1.76E+00 | 5.45E+00 |
| PA(16:0/16:0)[rac] Esi-15.755 | 9.61E-04 | 4.78E+00 | 5.21E+00 | 2.94E+00 | 6.61E+00 |
| PE(15:0/20:3(8Z,11Z,14Z)) Esi-15.158002 | 3.34E-02 | 5.04E+00 | 3.57E+00 | 2.24E+00 | 7.85E+00 |
| L-Tyrosine methyl ester 4-sulfate Esi-12.752999 | 3.52E-02 | -9.36E+00 | -4.26E+00 | -1.41E+01 | -4.68E+00 |
| C31 H10 N9 O13 | 3.38E-02 | -9.56E+00 | -4.32E+00 | -1.43E+01 | -4.85E+00 |
| PE(20:5(5Z,8Z,11Z,14Z,17Z)/P-18:1(11Z)) Esi-16.335 | 2.70E-02 | -4.39E+00 | -3.80E+00 | -6.71E+00 | -2.08E+00 |
| (R)-1-O-b-D-glucopyranosyl-1,3-octanediol Esi-10.488999 | 7.52E-03 | -1.15E+01 | -5.68E+00 | -1.58E+01 | -7.16E+00 |
| Mecarphon | 3.44E-02 | -8.96E+00 | -4.30E+00 | -1.34E+01 | -4.52E+00 |
| ADP / Adenosine 5'-diphosphate | 6.46E-136 | -8.69E+00 | -3.14E+01 | -9.24E+00 | -8.15E+00 |
| C26 H24 N14 S2 | 2.89E-02 | -9.82E+00 | -3.98E+00 | -1.49E+01 | -4.78E+00 |
| 4,4'-dinitrostilbene Esi-7.5989995 | 3.34E-02 | -8.36E+00 | -4.35E+00 | -1.24E+01 | -4.27E+00 |
| LPA(0:0/18:0) Esi-12.185999 | 6.19E-04 | 5.74E+00 | 5.15E+00 | 3.53E+00 | 7.95E+00 |
| Gatifloxacin | 3.34E-02 | -8.36E+00 | -4.38E+00 | -1.24E+01 | -4.29E+00 |
| PE(20:0/dm18:0) Esi-16.979002 | 2.38E-03 | -6.24E+00 | -4.82E+00 | -8.81E+00 | -3.66E+00 |
| PS(18:0/20:4(8Z,11Z,14Z,17Z)) Esi-16.744001 | 9.29E-07 | -7.81E+00 | -7.74E+00 | -9.84E+00 | -5.78E+00 |
| C16 H35 Cl N4 O4 | 3.39E-02 | 8.64E+00 | 4.05E+00 | 4.19E+00 | 1.31E+01 |
| PE(20:4(8Z,11Z,14Z,17Z)/22:0) Esi-13.239999 | 2.20E-02 | 3.92E+00 | 3.94E+00 | 1.92E+00 | 5.91E+00 |
| PS(18:0/20:0) Esi-14.913001 | 7.55E-03 | 4.34E+00 | 4.50E+00 | 2.40E+00 | 6.29E+00 |
| > limit Esi-15.753001 :1 | 8.30E-03 | 8.50E+00 | 5.56E+00 | 5.24E+00 | 1.18E+01 |
| 3-oxo-octanoyl-CoA | 2.43E-02 | 4.14E+00 | 3.89E+00 | 2.00E+00 | 6.28E+00 |
| 3-oxo-octanoyl-CoA Esi-14.174 | 2.31E-02 | 3.90E+00 | 3.92E+00 | 1.90E+00 | 5.91E+00 |
| PS(16:0/16:0) Esi-15.214 | 2.39E-02 | 4.82E+00 | 3.78E+00 | 2.28E+00 | 7.36E+00 |
| Aflatoxin-M1-8,9-epoxide Esi-4.001 | 3.34E-02 | -8.05E+00 | -4.37E+00 | -1.20E+01 | -4.12E+00 |
| Acetic acid Esi-8.024001 | 3.26E-02 | -2.91E+00 | -3.59E+00 | -4.51E+00 | -1.30E+00 |
| PE(14:0/P-18:1(11Z)) Esi-14.643 | 1.37E-02 | 5.23E+00 | 4.04E+00 | 2.66E+00 | 7.81E+00 |
| PE(14:0/P-18:1(11Z)) Esi-14.597 | 3.12E-02 | 4.81E+00 | 3.66E+00 | 2.20E+00 | 7.43E+00 |
| Acetyl-CoA | 1.62E-15 | -2.65E+00 | -9.58E+00 | -3.19E+00 | -2.11E+00 |
| 3-Octanol | 1.07E-02 | -4.55E+00 | -5.15E+00 | -6.41E+00 | -2.69E+00 |
| Austin | 2.61E-05 | 1.44E+01 | 7.94E+00 | 1.07E+01 | 1.82E+01 |
| Ailanthinone | 8.31E-03 | 1.07E+01 | 4.69E+00 | 6.03E+00 | 1.53E+01 |
| C34 H51 N O2 | 3.23E-02 | -8.83E+00 | -3.96E+00 | -1.34E+01 | -4.26E+00 |
| Magnoflorine Esi+5.628 | 3.24E-02 | 1.06E+01 | 4.04E+00 | 5.19E+00 | 1.61E+01 |
| C32 H49 N4 O | 1.01E-02 | -1.02E+01 | -4.66E+00 | -1.47E+01 | -5.73E+00 |
| C27 H30 O8 | 2.71E-03 | 1.18E+01 | 5.83E+00 | 7.60E+00 | 1.60E+01 |
| Myricanol 5-glucoside Esi+12.561002 | 2.29E-03 | 1.13E+01 | 5.77E+00 | 7.26E+00 | 1.54E+01 |
| Aspirin | 3.38E-02 | -9.48E+00 | -4.05E+00 | -1.44E+01 | -4.60E+00 |
| C27 H30 N3 O8 | 1.92E-03 | 1.08E+01 | 5.54E+00 | 6.85E+00 | 1.48E+01 |
| Safranin Esi+10.847997 | 4.87E-03 | 1.12E+01 | 5.46E+00 | 6.94E+00 | 1.55E+01 |
| C9 H19 N O | 1.96E-02 | -1.12E+00 | -4.33E+00 | -1.65E+00 | -5.85E-01 |
| L-Carnitine | 8.98E-05 | -1.84E+00 | -5.56E+00 | -2.49E+00 | -1.18E+00 |
| Atrovirinone Esi+11.502 | 1.02E-04 | 1.27E+01 | 6.90E+00 | 8.96E+00 | 1.65E+01 |
| C27 H54 N6 O7 | 4.29E-02 | -2.60E+00 | -3.76E+00 | -4.03E+00 | -1.18E+00 |
| C41 H46 N O8 | 2.48E-02 | 1.06E+01 | 4.06E+00 | 5.28E+00 | 1.60E+01 |
| 1-Nitrohexane | 1.72E-03 | 2.25E+00 | 6.15E+00 | 1.49E+00 | 3.01E+00 |
| 5-O-p-Coumaroylnigrumin Esi+12.287998 | 4.47E-04 | 1.25E+01 | 6.35E+00 | 8.47E+00 | 1.65E+01 |
| Tanavulgarol Esi+1.2039998 | 1.84E-02 | -1.91E+00 | -4.31E+00 | -2.82E+00 | -9.99E-01 |
| Medinoterb acetate | 4.21E-02 | -4.15E+00 | -3.73E+00 | -6.43E+00 | -1.87E+00 |
| alpha-Terpineol butanoate | 4.01E-04 | -2.74E+00 | -6.26E+00 | -3.64E+00 | -1.85E+00 |
| C30 H43 N O3 | 4.60E-03 | -1.07E+01 | -5.75E+00 | -1.46E+01 | -6.78E+00 |
| Myristicanol A Esi+13.464998 | 6.40E-03 | 1.12E+01 | 4.92E+00 | 6.52E+00 | 1.58E+01 |
| Clusin Esi+13.324998 | 2.81E-03 | 1.10E+01 | 5.50E+00 | 6.86E+00 | 1.51E+01 |
| Valeroidine | 2.24E-02 | -9.87E+00 | -4.28E+00 | -1.46E+01 | -5.10E+00 |
| C41 H44 N O9 | 3.42E-02 | 9.42E+00 | 3.83E+00 | 4.39E+00 | 1.44E+01 |
| Lactodifucotetraose Esi+13.322999 | 8.31E-03 | 1.11E+01 | 5.55E+00 | 6.85E+00 | 1.54E+01 |
| HC Blue No.1 | 4.27E-02 | -7.67E+00 | -3.94E+00 | -1.18E+01 | -3.58E+00 |
| PC(14:1(9Z)/20:5(5Z,8Z,11Z,14Z,17Z)) | 1.21E-08 | 1.79E+00 | 7.35E+00 | 1.31E+00 | 2.27E+00 |
| C18 H32 Cl2 N2 O4 S | 2.10E-02 | -2.02E+00 | -4.19E+00 | -3.01E+00 | -1.03E+00 |
| Myricanol 5-glucoside Esi+12.558 | 1.66E-02 | 9.45E+00 | 4.36E+00 | 5.00E+00 | 1.39E+01 |
| Flumethasone pivalate Esi+12.981001 | 4.39E-03 | 9.46E+00 | 5.10E+00 | 5.66E+00 | 1.33E+01 |
| Dunnione | 1.85E-02 | 8.75E+00 | 4.22E+00 | 4.51E+00 | 1.30E+01 |
| S-Adenosylmethionine | 3.63E-08 | -9.23E-01 | -6.92E+00 | -1.18E+00 | -6.61E-01 |
| C51 H86 N3 O14 | 1.53E-03 | -1.21E+01 | -6.63E+00 | -1.60E+01 | -8.26E+00 |
| PE(18:0/14:0) Esi+12.691002 | 1.99E-02 | 5.48E+00 | 3.87E+00 | 2.66E+00 | 8.31E+00 |
| Shanzhiside Esi+3.6849997 | 3.34E-02 | -9.30E+00 | -3.87E+00 | -1.42E+01 | -4.38E+00 |
| C10 H21 N O5 Esi+1.0029998 | 3.44E-02 | -2.03E+00 | -3.88E+00 | -3.10E+00 | -9.55E-01 |
| alpha-Terpineol butanoate Esi+1.123 | 3.07E-04 | -2.25E+00 | -6.48E+00 | -2.96E+00 | -1.54E+00 |
| Pheophytin a Esi+15.683004 | 1.97E-03 | 1.40E+01 | 5.74E+00 | 9.01E+00 | 1.91E+01 |
| Clethodim Esi+3.1679995 | 3.68E-02 | -1.93E+00 | -3.84E+00 | -2.96E+00 | -8.93E-01 |
| Docosanoic acid | 2.68E-02 | 9.18E+00 | 4.06E+00 | 4.55E+00 | 1.38E+01 |
| 5,10-Methylenetetrahydrofolate | 3.52E-02 | -3.66E+00 | -3.47E+00 | -5.74E+00 | -1.57E+00 |
| Italidipyrone Esi+12.1919985 | 5.28E-08 | 1.53E+01 | 1.10E+01 | 1.24E+01 | 1.81E+01 |
| Deltorphin C Esi+14.067995 | 4.30E-02 | 9.60E+00 | 3.76E+00 | 4.34E+00 | 1.49E+01 |
| C25 H48 N3 O6 Esi+14.398993 | 4.57E-02 | -2.39E+00 | -3.71E+00 | -3.71E+00 | -1.06E+00 |
| Choline Esi+1.3120002 | 4.80E-02 | -8.77E-01 | -3.33E+00 | -1.40E+00 | -3.57E-01 |
| L-Carnitine Esi+1.0449996 | 1.02E-04 | -1.72E+00 | -5.52E+00 | -2.33E+00 | -1.10E+00 |
| Atrovirinone Esi+11.498997 | 9.16E-05 | 1.34E+01 | 6.92E+00 | 9.42E+00 | 1.73E+01 |
| Clusin Esi+13.328999 | 3.39E-02 | 9.38E+00 | 4.07E+00 | 4.56E+00 | 1.42E+01 |
| Caryoptosidic acid Esi+3.6950002 | 3.35E-02 | -9.44E+00 | -3.84E+00 | -1.45E+01 | -4.42E+00 |
| 4-Hydroxyphenylpyruvic acid (HPPA) Esi+7.277 | 4.28E-02 | -6.67E+00 | -3.50E+00 | -1.05E+01 | -2.85E+00 |
| Kanokoside D Esi+13.6690035 | 1.11E-02 | 9.40E+00 | 4.56E+00 | 5.18E+00 | 1.36E+01 |
| L-4-Hydroxyglutamate semialdehyde | 2.96E-02 | -1.30E+00 | -4.04E+00 | -1.96E+00 | -6.38E-01 |
| Gelsedine | 3.49E-02 | -8.25E+00 | -3.79E+00 | -1.27E+01 | -3.81E+00 |
| Caryoptosidic acid Esi+4.572 | 4.89E-02 | -7.39E+00 | -3.87E+00 | -1.14E+01 | -3.38E+00 |
| Pheophytin a Esi+15.855001 | 7.52E-03 | 1.20E+01 | 5.56E+00 | 7.41E+00 | 1.65E+01 |
| PI(20:2(11Z,14Z)/18:2(9Z,12Z)) Esi+14.630002 | 3.20E-07 | 1.08E+01 | 7.62E+00 | 7.95E+00 | 1.36E+01 |
| 6-Epi-7-isocucurbic acid glucoside Esi+5.4489994 | 4.65E-02 | -8.61E+00 | -3.66E+00 | -1.34E+01 | -3.79E+00 |
| C28 H36 N3 O17 | 2.49E-02 | -9.07E+00 | -4.21E+00 | -1.35E+01 | -4.62E+00 |
| C7 H15 O3 | 1.42E-02 | 2.08E+00 | 4.43E+00 | 1.12E+00 | 3.04E+00 |
| Platydesminium Esi+1.134 | 4.65E-02 | -5.78E+00 | -3.97E+00 | -8.86E+00 | -2.70E+00 |
| L-isoleucyl-L-proline Esi+1.2010001 | 1.65E-03 | -2.96E+00 | -5.70E+00 | -4.02E+00 | -1.89E+00 |
| C32 H55 N O3 Esi+16.345 | 3.50E-02 | 7.94E+00 | 4.15E+00 | 3.90E+00 | 1.20E+01 |
| Diosbulbin E | 7.62E-03 | -1.14E+01 | -4.96E+00 | -1.62E+01 | -6.67E+00 |
| PC(14:1(9Z)/18:4(6Z,9Z,12Z,15Z)) | 1.75E-02 | 1.99E+00 | 3.86E+00 | 9.67E-01 | 3.01E+00 |
| PC(20:4(8Z,11Z,14Z,17Z)/20:4(8Z,11Z,14Z,17Z)) | 2.26E-04 | 5.25E+00 | 5.26E+00 | 3.28E+00 | 7.21E+00 |
| C34 H58 N17 O2 | 1.10E-02 | -9.82E+00 | -4.91E+00 | -1.40E+01 | -5.65E+00 |
| trans-Zeatin-7-?-D-glucoside | 1.59E-02 | -1.03E+01 | -4.35E+00 | -1.51E+01 | -5.43E+00 |
| Diethylene glycol | 6.79E-03 | -2.40E+00 | -5.57E+00 | -3.30E+00 | -1.49E+00 |
| C31 H30 N7 O3 | 3.89E-02 | 8.16E+00 | 3.91E+00 | 3.82E+00 | 1.25E+01 |
| Nilvadipine | 1.59E-02 | 1.09E+01 | 4.83E+00 | 6.13E+00 | 1.56E+01 |
| alpha-Cyclocitral Esi+11.418 | 1.94E-02 | 9.08E+00 | 4.29E+00 | 4.72E+00 | 1.34E+01 |
| 2,3-Butanediol apiosylglucoside Esi+3.0839999 | 3.25E-02 | -9.70E+00 | -4.01E+00 | -1.47E+01 | -4.71E+00 |
| Dihydromorphine | 4.21E-02 | 8.32E+00 | 3.80E+00 | 3.79E+00 | 1.29E+01 |
| 2,6-Di-tert-butyl-4-ethylphenol | 3.33E-02 | -9.78E+00 | -3.93E+00 | -1.49E+01 | -4.68E+00 |
| Red chlorophyll catabolite Esi+13.712001 | 3.44E-02 | 8.73E+00 | 4.29E+00 | 4.39E+00 | 1.31E+01 |
| Pseudoaconitine Esi+14.052001 | 3.63E-02 | 8.57E+00 | 4.04E+00 | 4.12E+00 | 1.30E+01 |
| trans-2,3-Dihydroxycinnamate Esi+4.704 | 1.99E-02 | -9.54E+00 | -4.58E+00 | -1.39E+01 | -5.16E+00 |
| 6-Hydroxyindolelactate Esi+6.900001 | 3.39E-02 | 8.68E+00 | 4.31E+00 | 4.39E+00 | 1.30E+01 |
| ibopamine Esi+8.212999 | 3.97E-02 | -8.79E+00 | -3.71E+00 | -1.36E+01 | -3.96E+00 |
| Carboxynorspermidine Esi+3.2750006 | 7.08E-04 | 1.18E+01 | 6.41E+00 | 8.01E+00 | 1.56E+01 |
| Antirrhinoside Esi+3.2389991 | 1.99E-02 | -9.59E+00 | -4.17E+00 | -1.43E+01 | -4.89E+00 |
| PC(14:1(9Z)/18:4(6Z,9Z,12Z,15Z)) Esi+12.56 | 1.39E-02 | 1.70E+00 | 3.98E+00 | 8.55E-01 | 2.55E+00 |
| (S)-Piperazine-2-carboxylic acid Esi+1.021 | 2.21E-02 | 2.21E+00 | 4.14E+00 | 1.12E+00 | 3.30E+00 |
| (1xi,3S)-1,2,3,4-Tetrahydro-1-methyl-beta-carboline-1,3-dicarboxylic acid Esi+0.816 | 3.27E-02 | -2.07E+00 | -4.01E+00 | -3.13E+00 | -1.01E+00 |
| PC(14:0/14:0) | 5.00E-03 | 4.38E+00 | 4.32E+00 | 2.38E+00 | 6.38E+00 |
| Scytalone | 2.16E-02 | -9.82E+00 | -4.13E+00 | -1.47E+01 | -4.97E+00 |
| 4,5-Dimethyl-2-(1-methylethyl)oxazole | 9.38E-03 | 9.89E+00 | 4.79E+00 | 5.64E+00 | 1.41E+01 |
| PC(18:3(6Z,9Z,12Z)/18:1(9Z)) | 2.87E-02 | 2.34E+00 | 3.65E+00 | 1.07E+00 | 3.61E+00 |
| Pheophorbide a Esi+13.593001 | 5.65E-03 | 1.06E+01 | 5.30E+00 | 6.46E+00 | 1.48E+01 |
| Acetylcarnitine | 3.48E-04 | -2.01E+00 | -5.86E+00 | -2.71E+00 | -1.32E+00 |
| Multifidol Esi+13.479997 | 3.52E-02 | 9.04E+00 | 4.26E+00 | 4.52E+00 | 1.36E+01 |
| C25 H36 N7 Esi+14.067996 | 4.27E-02 | 8.36E+00 | 3.68E+00 | 3.72E+00 | 1.30E+01 |
| C26 H54 Cl N4 O3 | 3.44E-02 | -8.51E+00 | -3.85E+00 | -1.30E+01 | -3.98E+00 |
| C10 H22 O4 | 1.02E-02 | 2.32E+00 | 4.93E+00 | 1.34E+00 | 3.30E+00 |
| PC(14:0/14:0) Esi+15.738004 | 1.38E-04 | 5.27E+00 | 5.38E+00 | 3.34E+00 | 7.20E+00 |
| Levocabastine Esi+12.300999 | 3.55E-02 | -8.57E+00 | -3.83E+00 | -1.32E+01 | -3.98E+00 |
| 1,2,10-Trihydroxydihydro-trans-linalyl oxide 7-O-beta-D-glucopyranoside Esi+1.2339998 | 3.74E-03 | -1.05E+01 | -5.56E+00 | -1.44E+01 | -6.56E+00 |
| Polixetonium chloride | 2.39E-02 | -2.08E+00 | -4.18E+00 | -3.10E+00 | -1.06E+00 |
| PC(22:1(13Z)/18:0) | 2.38E-03 | 4.21E+00 | 4.63E+00 | 2.42E+00 | 6.01E+00 |
| Sucrose | 5.60E-03 | -1.41E+00 | -4.52E+00 | -2.03E+00 | -7.85E-01 |
| 3'-N'-Acetylfusarochromanone | 4.18E-02 | -8.39E+00 | -3.81E+00 | -1.29E+01 | -3.83E+00 |
| 3-Hydroxydodecanedioic acid Esi+6.689 | 2.13E-02 | 1.05E+01 | 4.14E+00 | 5.32E+00 | 1.57E+01 |
| 2-(4-Allyl-2-methoxyphenoxy)-1-(4-hydroxy-3-methoxyphenyl)-1-propanol Esi+6.8029985 | 3.62E-02 | -8.47E+00 | -3.88E+00 | -1.30E+01 | -3.97E+00 |
| C20 H39 N O4 Esi+12.076002 | 4.71E-02 | -8.42E+00 | -3.62E+00 | -1.32E+01 | -3.68E+00 |
| C3 H11 N6 O Esi+1.021 | 3.65E-02 | 1.97E+00 | 3.77E+00 | 9.00E-01 | 3.03E+00 |
| C39 H44 N4 O7 | 3.42E-02 | 8.46E+00 | 3.83E+00 | 3.95E+00 | 1.30E+01 |
| Red chlorophyll catabolite Esi+13.712001 :1 | 3.44E-02 | 8.73E+00 | 4.29E+00 | 4.40E+00 | 1.31E+01 |
| 3-Hydroxydodecanedioic acid Esi+6.5930014 | 3.39E-02 | 9.55E+00 | 3.96E+00 | 4.56E+00 | 1.45E+01 |
| 3,4-Dihydro-7-methoxy-2-methylene-3-oxo-2H-1,4-benzoxazine-5-carboxylic acid | 1.71E-02 | -9.21E+00 | -4.92E+00 | -1.32E+01 | -5.22E+00 |
| PC(18:1(11Z)/14:1(9Z)) | 1.75E-07 | 5.55E+00 | 6.83E+00 | 3.95E+00 | 7.16E+00 |
| Sphinganine Esi+15.623996 | 2.49E-03 | -1.14E+01 | -5.50E+00 | -1.57E+01 | -7.15E+00 |
| Dimethyl suberate Esi+11.440998 | 4.66E-02 | -9.07E+00 | -3.64E+00 | -1.42E+01 | -3.97E+00 |
| Diethyl fumarate Esi+4.119001 | 3.05E-02 | -9.13E+00 | -3.98E+00 | -1.38E+01 | -4.43E+00 |
| C12 H23 N O3 | 1.10E-02 | -1.09E+01 | -4.89E+00 | -1.55E+01 | -6.23E+00 |
| L-Carnitine Esi+1.1829997 | 8.26E-03 | -2.32E+00 | -4.20E+00 | -3.42E+00 | -1.23E+00 |
| PC(15:0/20:4(5Z,8Z,11Z,14Z)) | 3.01E-24 | 1.15E+01 | 1.34E+01 | 9.76E+00 | 1.31E+01 |
| alpha-Terpineol butanoate Esi+0.79999965 | 3.48E-02 | -1.67E+00 | -3.84E+00 | -2.56E+00 | -7.78E-01 |
| PC(18:3(9Z,12Z,15Z)/20:1(11Z)) | 8.54E-03 | -3.87E+00 | -4.11E+00 | -5.72E+00 | -2.01E+00 |
| 2-Amino-3-methyl-9H-pyrido[2,3-b]indole | 4.21E-02 | 8.66E+00 | 3.81E+00 | 3.96E+00 | 1.34E+01 |
| Lipoamide Esi+1.1430002 | 2.48E-03 | -1.28E+00 | -4.63E+00 | -1.82E+00 | -7.32E-01 |
| (1x,2x)-Guaiacylglycerol 3-glucoside Esi+4.6230006 | 3.50E-02 | -8.90E+00 | -3.91E+00 | -1.36E+01 | -4.20E+00 |
| Simulanoquinoline Esi+12.804 | 1.58E-02 | 9.45E+00 | 4.57E+00 | 5.16E+00 | 1.37E+01 |
| C14 H26 O6 | 1.51E-03 | -1.39E+01 | -5.68E+00 | -1.89E+01 | -8.88E+00 |
| S-Adenosylmethionine Esi+6.8519993 | 7.78E-11 | 5.16E+00 | 7.96E+00 | 3.88E+00 | 6.43E+00 |
| (3S,5S)-Carbapenam-3-carboxylic acid | 4.34E-02 | 9.10E+00 | 3.70E+00 | 4.06E+00 | 1.41E+01 |
| Kiwiionoside Esi+5.7480006 | 4.66E-02 | -8.37E+00 | -3.73E+00 | -1.30E+01 | -3.74E+00 |
| 4-Hydroxyphenylpyruvic acid (HPPA) Esi+6.963 | 2.55E-03 | -7.85E+00 | -5.05E+00 | -1.10E+01 | -4.72E+00 |
| Labriformin Esi+13.718999 | 3.45E-02 | 9.33E+00 | 4.29E+00 | 4.69E+00 | 1.40E+01 |
| PC(20:5(5Z,8Z,11Z,14Z,17Z)/P-18:1(11Z)) Esi+14.279 | 5.01E-03 | -4.11E+00 | -4.32E+00 | -5.99E+00 | -2.23E+00 |
| C15 H22 O | 5.02E-03 | -2.07E+00 | -5.07E+00 | -2.91E+00 | -1.23E+00 |
| trans-3-Octen-2-ol Esi+5.3390007 | 3.38E-02 | 7.82E+00 | 4.28E+00 | 3.94E+00 | 1.17E+01 |
| C10 H22 O4 Esi+6.5859995 | 8.51E-03 | 2.04E+00 | 4.82E+00 | 1.17E+00 | 2.91E+00 |
| PC(14:0/14:0) Esi+15.104997 | 9.21E-03 | 4.01E+00 | 4.08E+00 | 2.07E+00 | 5.94E+00 |
| C21 H40 O10 | 3.50E-02 | -8.66E+00 | -4.17E+00 | -1.30E+01 | -4.27E+00 |
| 8-Oxodeoxycoformycin Esi+12.744002 | 3.34E-02 | 7.81E+00 | 4.35E+00 | 3.98E+00 | 1.16E+01 |
| Filicin Esi+13.479999 | 4.00E-04 | 1.33E+01 | 6.32E+00 | 9.01E+00 | 1.76E+01 |
| Ethyl vanillin isobutyrate Esi+6.164001 | 9.34E-03 | -1.01E+01 | -4.65E+00 | -1.46E+01 | -5.68E+00 |
| 5'-Deoxy-5'-fluoroadenosine Esi+5.358001 | 3.30E-02 | -9.02E+00 | -4.38E+00 | -1.34E+01 | -4.64E+00 |
| C29 H48 N16 O3 | 4.23E-02 | -8.89E+00 | -3.73E+00 | -1.38E+01 | -4.00E+00 |
| C12 H22 N14 O3 | 4.53E-02 | -7.95E+00 | -3.64E+00 | -1.24E+01 | -3.49E+00 |
| Methyleugenol | 1.61E-02 | 8.80E+00 | 4.99E+00 | 5.04E+00 | 1.26E+01 |
| PE(15:0/16:0) | 6.65E-04 | 4.67E+00 | 5.39E+00 | 2.94E+00 | 6.40E+00 |
| 8S-HODE Esi+11.869999 | 4.68E-02 | -8.11E+00 | -3.65E+00 | -1.27E+01 | -3.56E+00 |
| Isoferulic acid Esi+5.367998 | 4.97E-05 | -1.30E+01 | -7.28E+00 | -1.66E+01 | -9.32E+00 |
| Spiromesifen Esi+5.0250006 | 3.87E-02 | -7.97E+00 | -3.84E+00 | -1.23E+01 | -3.69E+00 |
| 3-Acetyl-6-methoxybenzaldehyde | 1.70E-02 | -8.51E+00 | -4.92E+00 | -1.22E+01 | -4.83E+00 |
| 6-Amino-9H-purine-9-propanoic acid Esi+7.4700003 | 1.97E-03 | 1.04E+01 | 5.74E+00 | 6.64E+00 | 1.41E+01 |
| Primary fluorescent chlorophyll catabolite Esi+13.199998 | 1.32E-02 | 1.01E+01 | 4.55E+00 | 5.53E+00 | 1.47E+01 |
| C18 H40 N7 O15 | 4.97E-02 | 7.46E+00 | 3.68E+00 | 3.28E+00 | 1.16E+01 |
| Capillartemisin B Esi+10.821001 | 3.63E-02 | 8.98E+00 | 3.79E+00 | 4.13E+00 | 1.38E+01 |
| Pheophytin a Esi+16.351004 | 9.05E-03 | 1.32E+01 | 5.32E+00 | 7.96E+00 | 1.85E+01 |
| Simulanoquinoline Esi+13.324998 | 3.23E-02 | 9.28E+00 | 4.02E+00 | 4.52E+00 | 1.40E+01 |
| NNAL-N-glucuronide Esi+5.144 | 2.78E-02 | -9.52E+00 | -4.11E+00 | -1.43E+01 | -4.75E+00 |
| PC(14:0/14:0) Esi+14.22 | 1.43E-02 | 3.79E+00 | 3.89E+00 | 1.87E+00 | 5.71E+00 |
| 2-cis,6-cis-farnesyl diphosphate Esi+2.9749994 | 1.93E-02 | -9.63E+00 | -4.20E+00 | -1.43E+01 | -4.94E+00 |
| PC(15:0/22:5(4Z,7Z,10Z,13Z,16Z)) | 1.83E-02 | -3.06E+00 | -3.78E+00 | -4.66E+00 | -1.46E+00 |
| C32 H18 N | 2.66E-02 | -7.89E+00 | -4.18E+00 | -1.18E+01 | -3.99E+00 |
| C21 H26 N24 Esi+13.720997 | 2.87E-02 | 9.11E+00 | 4.14E+00 | 4.56E+00 | 1.37E+01 |
| S-Adenosylmethionine Esi+5.265 | 4.09E-09 | 4.26E+00 | 7.34E+00 | 3.12E+00 | 5.39E+00 |
| (3beta,5alpha,9alpha,14alpha,22E,24R)-3,5,9,14-Tetrahydroxyergosta-7,22-dien-6-one Esi+7.052001 | 4.75E-02 | -8.40E+00 | -3.62E+00 | -1.31E+01 | -3.66E+00 |
| MS 3 Esi+12.9819975 | 3.49E-02 | 8.34E+00 | 3.81E+00 | 3.86E+00 | 1.28E+01 |
| 8-(3,3-Dimethylallyl)-3-methylgalangin | 3.61E-02 | 7.70E+00 | 4.24E+00 | 3.83E+00 | 1.16E+01 |
| Dihydrozeatin Esi+1.0480002 | 9.93E-03 | -2.33E+00 | -4.88E+00 | -3.31E+00 | -1.34E+00 |
| PI(20:2(11Z,14Z)/16:0) | 3.62E-04 | 8.65E+00 | 5.85E+00 | 5.67E+00 | 1.16E+01 |
| C26 H24 N O2 S | 3.34E-02 | -8.71E+00 | -3.87E+00 | -1.33E+01 | -4.11E+00 |
| trans-3-Octen-2-ol Esi+5.3379993 | 1.49E-04 | 1.31E+01 | 8.68E+00 | 9.90E+00 | 1.63E+01 |
| 2,6-Cyclolycopene-1,5-diol | 3.05E-02 | -2.66E+00 | -3.99E+00 | -4.03E+00 | -1.30E+00 |
| PC(24:1(15Z)/20:3(5Z,8Z,11Z)) | 3.36E-07 | 5.00E+00 | 6.76E+00 | 3.54E+00 | 6.45E+00 |
| 26-Hydroxybrassinolide Esi+11.786 | 3.34E-02 | 8.97E+00 | 4.35E+00 | 4.57E+00 | 1.34E+01 |
| PC(14:0/14:0) Esi+14.201997 | 1.30E-02 | 3.84E+00 | 3.94E+00 | 1.92E+00 | 5.76E+00 |
| all-trans-Undecaprenyl phosphate | 4.46E-02 | 8.16E+00 | 3.78E+00 | 3.69E+00 | 1.26E+01 |
| [8]-Paradol | 1.62E-02 | -9.41E+00 | -4.97E+00 | -1.34E+01 | -5.37E+00 |
| C19 H19 N8 O | 9.21E-03 | -9.27E+00 | -4.80E+00 | -1.32E+01 | -5.29E+00 |
| C14 H29 Cl2 O S | 3.32E-02 | 9.32E+00 | 3.95E+00 | 4.48E+00 | 1.42E+01 |
| PC(18:0/24:1(15Z)) | 1.03E-07 | 5.74E+00 | 7.00E+00 | 4.12E+00 | 7.36E+00 |
| Moschamindole | 3.44E-02 | -8.01E+00 | -4.23E+00 | -1.20E+01 | -3.99E+00 |
| C14 H12 N | 3.17E-03 | 1.01E+01 | 5.24E+00 | 6.13E+00 | 1.40E+01 |
| Dutasteride Esi+5.785001 | 4.87E-02 | -8.21E+00 | -3.64E+00 | -1.28E+01 | -3.58E+00 |
| HC Blue No.1 Esi+1.125 | 4.13E-02 | -8.17E+00 | -3.95E+00 | -1.25E+01 | -3.84E+00 |
| Dimethoxane Esi+11.439 | 2.48E-02 | -8.85E+00 | -4.19E+00 | -1.32E+01 | -4.49E+00 |
| Thenylchlor | 1.07E-02 | -9.71E+00 | -4.56E+00 | -1.41E+01 | -5.36E+00 |
| C7 H20 N5 O2 | 4.46E-03 | 1.57E+00 | 5.10E+00 | 9.40E-01 | 2.20E+00 |
| Pseudoaconitine Esi+13.977 | 4.66E-02 | 9.81E+00 | 3.65E+00 | 4.31E+00 | 1.53E+01 |
| Dikegulac Esi+6.175001 | 1.94E-02 | 8.83E+00 | 4.19E+00 | 4.52E+00 | 1.31E+01 |
| PC(14:0/20:5(5Z,8Z,11Z,14Z,17Z)) | 1.14E-02 | -3.71E+00 | -3.99E+00 | -5.55E+00 | -1.88E+00 |
| C22 H33 N13 | 1.10E-02 | -9.20E+00 | -4.70E+00 | -1.32E+01 | -5.16E+00 |
| C12 H16 O3 | 1.65E-02 | 9.39E+00 | 4.95E+00 | 5.35E+00 | 1.34E+01 |
| LysoPC(20:3(5Z,8Z,11Z)) Esi+13.712003 | 3.45E-02 | -6.13E+00 | -3.59E+00 | -9.55E+00 | -2.72E+00 |
| C23 H47 Cl N6 O2 S3 Esi+13.069 | 3.64E-02 | 8.19E+00 | 3.79E+00 | 3.77E+00 | 1.26E+01 |
| Roxburghine B Esi+5.7180004 | 2.89E-02 | -9.41E+00 | -4.02E+00 | -1.42E+01 | -4.61E+00 |
| Kynostatin 272 Esi+13.475999 | 3.35E-02 | 7.97E+00 | 4.34E+00 | 4.06E+00 | 1.19E+01 |
| PC(14:1(9Z)/15:0) Esi+13.127006 | 1.14E-02 | 3.27E+00 | 4.00E+00 | 1.66E+00 | 4.88E+00 |
| N-trans-Feruloyl-4-O-methyldopamine Esi+6.031 | 3.80E-02 | 8.40E+00 | 3.77E+00 | 3.83E+00 | 1.30E+01 |
| Carpacin Esi+13.330001 | 5.16E-03 | 1.10E+01 | 5.44E+00 | 6.76E+00 | 1.52E+01 |
| C24 H23 N12 | 4.19E-02 | -7.97E+00 | -3.78E+00 | -1.23E+01 | -3.62E+00 |
| PI(18:3(9Z,12Z,15Z)/18:1(11Z)) Esi+14.121 | 1.60E-02 | -5.61E+00 | -4.30E+00 | -8.27E+00 | -2.94E+00 |
| NNAL-N-glucuronide Esi+5.052001 | 9.71E-03 | -1.02E+01 | -4.62E+00 | -1.48E+01 | -5.70E+00 |
| Tetrahydropteridine | 3.14E-02 | 7.94E+00 | 4.35E+00 | 4.08E+00 | 1.18E+01 |
| PE(18:0/14:0) Esi+12.691002 :1 | 1.99E-02 | 5.48E+00 | 3.87E+00 | 2.66E+00 | 8.31E+00 |
| N3'-Acetylneomycin Esi+14.600004 | 1.61E-02 | 9.12E+00 | 4.85E+00 | 5.14E+00 | 1.31E+01 |
| PI(20:2(11Z,14Z)/16:0) Esi+13.699998 | 6.32E-04 | 8.01E+00 | 5.53E+00 | 5.10E+00 | 1.09E+01 |
| Nigakilactone L Esi+13.273998 | 3.30E-02 | 8.64E+00 | 3.93E+00 | 4.13E+00 | 1.32E+01 |
| C22 H41 Cl2 N2 O4 | 1.30E-02 | -9.08E+00 | -4.53E+00 | -1.32E+01 | -4.97E+00 |
| PI(20:3(5Z,8Z,11Z)/18:1(11Z)) Esi+14.531002 | 3.34E-02 | 6.21E+00 | 3.67E+00 | 2.82E+00 | 9.60E+00 |
| Tryptophyl-Threonine | 3.08E-02 | -2.15E+00 | -3.95E+00 | -3.26E+00 | -1.04E+00 |
| PI(20:2(11Z,14Z)/18:2(9Z,12Z)) Esi+14.845 | 1.20E-02 | 7.59E+00 | 4.17E+00 | 3.95E+00 | 1.12E+01 |
| Tridemorph Esi+16.444 | 3.67E-03 | 1.03E+01 | 5.47E+00 | 6.42E+00 | 1.43E+01 |
| C19 H38 N16 O7 | 3.40E-02 | 8.44E+00 | 3.95E+00 | 4.02E+00 | 1.29E+01 |
| Caffeoyl tyrosine | 4.07E-02 | 8.27E+00 | 3.71E+00 | 3.71E+00 | 1.28E+01 |
| PC(14:1(9Z)/22:6(4Z,7Z,10Z,13Z,16Z,19Z)) | 2.64E-03 | -3.43E+00 | -4.58E+00 | -4.91E+00 | -1.95E+00 |
| Primary fluorescent chlorophyll catabolite Esi+13.464997 | 1.73E-02 | 1.07E+01 | 4.26E+00 | 5.58E+00 | 1.59E+01 |
| C33 H40 N13 O2 Esi+13.479998 | 3.60E-03 | 1.19E+01 | 5.25E+00 | 7.26E+00 | 1.66E+01 |
| Phyllanthusol A Esi+9.569 | 1.24E-02 | -9.10E+00 | -4.48E+00 | -1.33E+01 | -4.95E+00 |
| 5a,11a-Dehydrooxytetracycline Esi+8.813003 | 2.43E-02 | -8.70E+00 | -4.10E+00 | -1.30E+01 | -4.36E+00 |
| Finaconitine Esi+13.386996 | 7.32E-03 | 9.26E+00 | 4.93E+00 | 5.40E+00 | 1.31E+01 |
| Millefin Esi+8.518998 | 3.71E-02 | 8.78E+00 | 3.83E+00 | 4.07E+00 | 1.35E+01 |
| Tridemorph Esi+15.783998 | 7.30E-03 | 9.94E+00 | 5.72E+00 | 6.24E+00 | 1.37E+01 |
| PC(18:4(6Z,9Z,12Z,15Z)/16:0) Esi+13.938999 | 3.44E-02 | -1.65E+00 | -3.49E+00 | -2.59E+00 | -7.17E-01 |
| Chimyl alcohol Esi+12.925002 | 1.52E-02 | -9.66E+00 | -4.40E+00 | -1.42E+01 | -5.16E+00 |
| C17 H34 N20 O3 | 1.73E-02 | 9.08E+00 | 4.73E+00 | 5.03E+00 | 1.31E+01 |
| Coproporphyrinogen I Esi+13.647002 | 3.34E-02 | 9.47E+00 | 4.10E+00 | 4.66E+00 | 1.43E+01 |
| PE(14:0/14:1(9Z)) Esi+14.578002 | 5.80E-03 | 5.66E+00 | 4.46E+00 | 3.13E+00 | 8.19E+00 |
| LysoPC(20:4(5Z,8Z,11Z,14Z)) Esi+13.821 | 4.89E-02 | -5.78E+00 | -3.42E+00 | -9.16E+00 | -2.40E+00 |
| PE(14:0/14:1(9Z)) Esi+14.210002 | 2.98E-03 | 5.68E+00 | 4.72E+00 | 3.28E+00 | 8.07E+00 |
| Sambacin Esi+5.255999 | 3.39E-02 | -8.70E+00 | -3.84E+00 | -1.33E+01 | -4.07E+00 |
| PC(22:6(4Z,7Z,10Z,13Z,16Z,19Z)/15:0) | 3.35E-02 | -3.08E+00 | -3.49E+00 | -4.82E+00 | -1.34E+00 |
| PC(20:5(5Z,8Z,11Z,14Z,17Z)/P-18:1(11Z)) Esi+14.381998 | 3.34E-02 | -3.23E+00 | -3.51E+00 | -5.04E+00 | -1.42E+00 |
| 10-Carboxy-13-deoxycarminomycin Esi+5.7279983 | 4.28E-02 | -8.03E+00 | -4.09E+00 | -1.22E+01 | -3.85E+00 |
| C22 H20 N4 O2 | 3.34E-02 | 8.19E+00 | 4.05E+00 | 3.98E+00 | 1.24E+01 |
| 1,20-Eicosanediol Esi+13.8099985 | 3.45E-02 | -8.78E+00 | -4.29E+00 | -1.31E+01 | -4.42E+00 |
| Coproporphyrin I Esi+13.777001 | 3.75E-02 | 8.47E+00 | 4.21E+00 | 4.18E+00 | 1.28E+01 |
| C28 H32 N24 O4 | 3.34E-02 | 7.72E+00 | 4.37E+00 | 3.95E+00 | 1.15E+01 |
| C22 H47 Cl N7 O7 S | 5.01E-03 | 1.14E+01 | 4.99E+00 | 6.71E+00 | 1.60E+01 |
| Capsi-amide Esi+14.821 | 1.62E-02 | 8.45E+00 | 4.98E+00 | 4.83E+00 | 1.21E+01 |
| PE(15:0/16:0) Esi+14.828002 | 2.88E-03 | 4.30E+00 | 4.83E+00 | 2.51E+00 | 6.08E+00 |
| PI(20:2(11Z,14Z)/18:2(9Z,12Z)) Esi+15.036001 | 9.07E-03 | 7.53E+00 | 4.30E+00 | 4.03E+00 | 1.10E+01 |
| Alfentanil Esi+12.558 | 7.57E-03 | -1.00E+01 | -5.66E+00 | -1.38E+01 | -6.25E+00 |
| Xanthoplanine Esi+13.608997 | 7.62E-03 | 9.61E+00 | 5.65E+00 | 5.98E+00 | 1.32E+01 |
| PC(18:4(6Z,9Z,12Z,15Z)/20:5(5Z,8Z,11Z,14Z,17Z)) | 1.07E-02 | -2.77E+00 | -4.04E+00 | -4.12E+00 | -1.41E+00 |
| C35 H41 Cl N9 O | 2.14E-02 | 9.26E+00 | 4.16E+00 | 4.71E+00 | 1.38E+01 |
| Malvidin 3-sophoroside-5-glucoside | 1.61E-02 | -8.51E+00 | -4.98E+00 | -1.22E+01 | -4.87E+00 |
| PE(15:0/16:0) Esi+14.069 | 1.80E-02 | 5.56E+00 | 3.87E+00 | 2.71E+00 | 8.42E+00 |
| C25 H45 N Esi+13.6640005 | 4.99E-02 | -7.82E+00 | -3.62E+00 | -1.23E+01 | -3.39E+00 |
| PE(22:5(7Z,10Z,13Z,16Z,19Z)/15:0) Esi+14.401 | 4.57E-02 | -4.42E+00 | -3.43E+00 | -6.99E+00 | -1.85E+00 |
| Red chlorophyll catabolite Esi+13.7109995 | 1.81E-02 | 9.59E+00 | 4.87E+00 | 5.40E+00 | 1.38E+01 |
| C31 H26 N4 O3 | 4.50E-06 | 1.16E+01 | 8.23E+00 | 8.69E+00 | 1.44E+01 |
| Tsugaric acid C Esi+13.244998 | 1.07E-02 | -9.67E+00 | -4.57E+00 | -1.40E+01 | -5.34E+00 |
| Primary fluorescent chlorophyll catabolite Esi+13.464997 :1 | 3.22E-03 | 1.27E+01 | 6.37E+00 | 8.45E+00 | 1.69E+01 |
| KOdiA-PC Esi+12.681 | 3.35E-02 | -8.97E+00 | -4.04E+00 | -1.36E+01 | -4.35E+00 |
| Red chlorophyll catabolite Esi+12.976003 | 3.21E-02 | 8.40E+00 | 4.02E+00 | 4.09E+00 | 1.27E+01 |
| C37 H78 N2 S4 | 8.05E-03 | 1.07E+01 | 4.74E+00 | 6.06E+00 | 1.53E+01 |
| 5-O-?-D-Mycaminosyltylactone Esi+13.580001 | 1.67E-02 | -9.27E+00 | -4.32E+00 | -1.37E+01 | -4.88E+00 |
| (R)-1-O-b-D-glucopyranosyl-1,3-octanediol Esi+10.494998 | 1.47E-02 | -1.26E+01 | -4.42E+00 | -1.85E+01 | -6.78E+00 |
| Pheophorbide a Esi+13.037002 | 4.38E-02 | 9.49E+00 | 3.70E+00 | 4.23E+00 | 1.48E+01 |
| 1,2-Di-(9Z,12Z,15Z-octadecatrienoyl)-3-(Galactosyl-alpha-1-6-Galactosyl-beta-1)-glycerol Esi+16.208006 | 1.53E-02 | -1.00E+01 | -4.80E+00 | -1.44E+01 | -5.62E+00 |
| PC(15:0/20:4(5Z,8Z,11Z,14Z)) Esi+14.052 | 1.43E-14 | -9.29E+00 | -9.68E+00 | -1.12E+01 | -7.40E+00 |
| (-)-menthyl beta-D-glucoside Esi+13.088 | 4.67E-02 | -9.88E+00 | -3.67E+00 | -1.54E+01 | -4.35E+00 |
| Ribalinium | 3.73E-03 | -1.34E+01 | -6.23E+00 | -1.80E+01 | -8.85E+00 |
| Histidinyl-Gamma-glutamate | 3.58E-03 | -1.35E+01 | -5.78E+00 | -1.83E+01 | -8.59E+00 |
| 1,2-Di-(9Z,12Z,15Z-octadecatrienoyl)-3-(Galactosyl-alpha-1-6-Galactosyl-beta-1)-glycerol Esi+14.911002 | 1.14E-02 | -1.17E+01 | -5.12E+00 | -1.66E+01 | -6.88E+00 |
| C36 H56 O3 Esi+16.039 | 1.42E-02 | -1.07E+01 | -4.73E+00 | -1.54E+01 | -5.96E+00 |
| 16alpha,17alpha-Dihydroxyprogesterone acetophenide Esi+16.516003 | 3.51E-03 | -1.33E+01 | -5.69E+00 | -1.82E+01 | -8.44E+00 |
| EPTC Esi+13.395997 | 3.54E-03 | -1.33E+01 | -5.78E+00 | -1.81E+01 | -8.46E+00 |
| Pheophorbide a Esi+13.031003 | 1.80E-02 | 1.03E+01 | 4.26E+00 | 5.37E+00 | 1.53E+01 |
| C36 H56 O3 Esi+15.1919985 | 4.84E-02 | -8.80E+00 | -3.61E+00 | -1.38E+01 | -3.82E+00 |
| Morroniside Esi+2.799 | 1.94E-02 | -9.74E+00 | -4.21E+00 | -1.45E+01 | -5.01E+00 |
| 1-(4-Hydroxy-2-methylpyrimid-5-ylmethyl)-3-(beta-hydroxyethyl)-2-methylpyridinium Esi+7.502999 | 3.49E-02 | -8.96E+00 | -4.27E+00 | -1.34E+01 | -4.49E+00 |
| Dihydrofolic acid Esi+4.275001 | 1.32E-04 | -9.67E+00 | -6.05E+00 | -1.29E+01 | -6.46E+00 |
| Hopane-29-acetate | 8.31E-03 | -1.19E+01 | -4.88E+00 | -1.69E+01 | -6.86E+00 |
| C30 H43 N O3 Esi+13.069 | 3.81E-02 | -8.98E+00 | -3.85E+00 | -1.38E+01 | -4.16E+00 |
| C8 H5 N O7 | 2.88E-03 | -1.36E+01 | -6.48E+00 | -1.80E+01 | -9.10E+00 |
| Clavamycin F Esi+2.8530004 | 7.26E-04 | -1.33E+01 | -7.63E+00 | -1.71E+01 | -9.60E+00 |
| Swertiamarin Esi+5.035001 | 3.25E-02 | -9.99E+00 | -3.94E+00 | -1.52E+01 | -4.79E+00 |
| Cyclonormammein Esi+12.485995 | 3.76E-02 | 8.04E+00 | 3.78E+00 | 3.68E+00 | 1.24E+01 |
| Pheophytin a Esi+13.940001 | 7.02E-03 | 1.14E+01 | 4.82E+00 | 6.55E+00 | 1.62E+01 |
| Kelampayoside A Esi+3.0330005 | 3.61E-03 | -1.01E+01 | -5.18E+00 | -1.41E+01 | -6.14E+00 |
| SB 200646 Esi+6.5370007 | 3.44E-02 | -1.05E+01 | -3.82E+00 | -1.61E+01 | -4.88E+00 |
| Betulin Esi+15.4559965 | 1.14E-03 | -1.23E+01 | -5.77E+00 | -1.67E+01 | -7.97E+00 |
| Plumieride Esi+3.4529994 | 2.40E-03 | -1.17E+01 | -5.56E+00 | -1.60E+01 | -7.38E+00 |
| (E)-2,6-Dimethyl-2,5-heptadienoic acid | 2.43E-04 | -1.27E+01 | -6.58E+00 | -1.67E+01 | -8.76E+00 |
| PG(18:0/20:3(5Z,8Z,11Z)) Esi+13.919003 | 8.16E-03 | -1.12E+01 | -4.89E+00 | -1.59E+01 | -6.48E+00 |
| C12 H20 N11 O4 | 1.10E-02 | -9.99E+00 | -5.09E+00 | -1.41E+01 | -5.86E+00 |
| Benzyl gentiobioside Esi+3.34 | 1.61E-02 | -1.05E+01 | -4.55E+00 | -1.52E+01 | -5.68E+00 |
| 5-(3'-Carboxy-3'-oxopropenyl)-4,6-dihydroxypicolinate | 8.05E-03 | -1.09E+01 | -5.58E+00 | -1.51E+01 | -6.74E+00 |
| Arachidonic Acid (d8) | 5.54E-06 | -1.15E+01 | -8.02E+00 | -1.44E+01 | -8.56E+00 |
| Erythromycin D Esi+15.002 | 2.53E-02 | -1.13E+01 | -4.19E+00 | -1.68E+01 | -5.72E+00 |
| C30 H49 N O2 | 3.32E-02 | -9.02E+00 | -4.16E+00 | -1.36E+01 | -4.48E+00 |
| Carbenicillin Esi+5.269999 | 3.34E-02 | -9.27E+00 | -3.85E+00 | -1.42E+01 | -4.36E+00 |
| Morroniside Esi+2.648 | 4.66E-02 | -8.98E+00 | -3.64E+00 | -1.40E+01 | -3.94E+00 |
| Verbasoside Esi+5.927001 | 4.98E-03 | -1.05E+01 | -5.37E+00 | -1.45E+01 | -6.42E+00 |
| (7'R)-(+)-Lyoniresinol 9'-glucoside Esi+6.0849996 | 3.34E-02 | -9.65E+00 | -3.98E+00 | -1.46E+01 | -4.65E+00 |
| C29 H42 N6 O6 | 3.45E-02 | 9.26E+00 | 3.87E+00 | 4.35E+00 | 1.42E+01 |
| Benzoyl phosphate Esi+9.117999 | 7.52E-03 | -1.19E+01 | -5.68E+00 | -1.64E+01 | -7.46E+00 |
| (3R,7R)-1,3,7-Octanetriol Esi+10.496001 | 1.99E-02 | -1.00E+01 | -4.23E+00 | -1.49E+01 | -5.16E+00 |
| Silandrin | 3.34E-02 | -9.55E+00 | -4.34E+00 | -1.42E+01 | -4.86E+00 |
| Linalool 3,7-oxide beta-primeveroside Esi+6.129999 | 3.41E-02 | -9.23E+00 | -3.86E+00 | -1.41E+01 | -4.33E+00 |
| PC(15:0/14:1(9Z)) | 3.60E-03 | 4.18E+00 | 4.43E+00 | 2.32E+00 | 6.04E+00 |
| C15 H9 N2 O S | 3.84E-02 | -8.92E+00 | -3.78E+00 | -1.38E+01 | -4.08E+00 |
| C25 H31 N3 O3 | 3.52E-02 | -8.37E+00 | -3.81E+00 | -1.29E+01 | -3.87E+00 |
| 2-Methylpropyl glucosinolate | 1.75E-02 | -1.02E+01 | -4.90E+00 | -1.46E+01 | -5.76E+00 |
| Gentianose | 1.42E-02 | -9.87E+00 | -4.97E+00 | -1.41E+01 | -5.67E+00 |
| S-Adenosylmethionine Esi+12.137 | 9.01E-03 | 2.55E+00 | 4.04E+00 | 1.31E+00 | 3.79E+00 |
| 3'-Glucosyl-2',4',6'-trihydroxyacetophenone Esi+3.3180006 | 3.69E-02 | -9.38E+00 | -3.77E+00 | -1.45E+01 | -4.29E+00 |
| C21 H31 N15 O S | 3.86E-02 | -8.34E+00 | -3.73E+00 | -1.29E+01 | -3.78E+00 |
| Istamycin B0 Esi+9.606001 | 1.49E-02 | -9.62E+00 | -4.62E+00 | -1.39E+01 | -5.30E+00 |
| Tris(butoxyethyl)phosphate Esi+4.889 | 3.49E-02 | -9.02E+00 | -3.93E+00 | -1.38E+01 | -4.27E+00 |
| Coleone A Esi+6.951002 | 1.69E-05 | -1.31E+01 | -8.83E+00 | -1.62E+01 | -1.00E+01 |
| HDMBOA-Glc Esi+1.2420001 | 2.01E-03 | -1.03E+01 | -5.68E+00 | -1.40E+01 | -6.57E+00 |
| Citrusin F Esi+2.8660002 | 1.07E-02 | -9.78E+00 | -4.57E+00 | -1.42E+01 | -5.41E+00 |
| Verbasoside Esi+6.115999 | 2.41E-02 | -8.96E+00 | -4.08E+00 | -1.34E+01 | -4.48E+00 |
| PI(20:2(11Z,14Z)/18:2(9Z,12Z)) Esi+13.960001 | 3.23E-02 | 6.71E+00 | 3.68E+00 | 3.07E+00 | 1.04E+01 |
| Tanakamine | 3.31E-03 | -1.20E+01 | -5.71E+00 | -1.64E+01 | -7.62E+00 |
| (S)-Multifidol 2-[apiosyl-(1-6)-glucoside] Esi+3.841 | 3.32E-02 | -9.19E+00 | -3.89E+00 | -1.40E+01 | -4.36E+00 |
| Kuwanon Q Esi+7.817001 | 1.68E-02 | -8.96E+00 | -4.42E+00 | -1.31E+01 | -4.78E+00 |
| 8-Acetoxy-4'-methoxypinoresinol 4-glucoside Esi+6.327 | 3.45E-02 | -8.44E+00 | -4.18E+00 | -1.27E+01 | -4.17E+00 |
| EPTC Esi+13.158998 | 1.12E-02 | -1.05E+01 | -4.93E+00 | -1.50E+01 | -6.05E+00 |
| Vanillyl alcohol Esi+3.1499999 | 3.37E-02 | -9.24E+00 | -4.33E+00 | -1.38E+01 | -4.69E+00 |
| Isoferulic acid Esi+5.657 | 9.94E-03 | -1.08E+01 | -4.71E+00 | -1.55E+01 | -6.10E+00 |
| Fenbendazole | 2.48E-03 | -1.32E+01 | -6.65E+00 | -1.74E+01 | -8.94E+00 |
| Citrusin B Esi+6.321999 | 2.33E-02 | -9.56E+00 | -4.12E+00 | -1.43E+01 | -4.81E+00 |
| 4-Methylthiobutanaldoxime | 7.64E-03 | -1.13E+01 | -5.63E+00 | -1.55E+01 | -7.00E+00 |
| 5-Amino-6-(4-hydroxy-2-butenoyl)-2,2-dimethyl-4-chromanone Esi+12.102 | 2.81E-03 | -1.20E+01 | -6.51E+00 | -1.59E+01 | -8.06E+00 |
| (-)-Epicatechin Esi+13.397002 | 1.37E-02 | -1.00E+01 | -4.72E+00 | -1.44E+01 | -5.61E+00 |
| Profluralin Esi+3.0680006 | 1.45E-02 | -9.82E+00 | -5.01E+00 | -1.40E+01 | -5.66E+00 |
| 2-Methoxyestrone 3-sulfate | 3.14E-02 | -8.71E+00 | -4.04E+00 | -1.32E+01 | -4.27E+00 |
| C11 H25 Cl N3 O5 S2 | 3.34E-02 | -9.93E+00 | -3.91E+00 | -1.51E+01 | -4.72E+00 |
| Tetradecanoyl-CoA | 4.20E-06 | -6.91E+00 | -6.29E+00 | -9.09E+00 | -4.74E+00 |
| Levosimendan Esi+3.9800012 | 3.65E-02 | -8.67E+00 | -4.18E+00 | -1.31E+01 | -4.27E+00 |
| N10-Formyltetrahydrofolic acid Esi+3.8959987 | 4.75E-03 | -5.70E+00 | -4.49E+00 | -8.22E+00 | -3.17E+00 |
| Zizybeoside I Esi+3.4640005 | 3.44E-02 | -9.15E+00 | -3.88E+00 | -1.40E+01 | -4.30E+00 |
| PC(14:0/18:1(9Z)) | 2.32E-05 | -4.14E+00 | -6.00E+00 | -5.51E+00 | -2.77E+00 |
| Phenylethyl primeveroside Esi+4.7580013 | 3.83E-02 | -8.24E+00 | -3.73E+00 | -1.28E+01 | -3.74E+00 |
| PC(16:1(9Z)/14:1(9Z)) | 1.30E-02 | -3.78E+00 | -3.94E+00 | -5.67E+00 | -1.89E+00 |
| PE(22:2(13Z,16Z)/20:4(8Z,11Z,14Z,17Z)) Esi+13.845 | 1.98E-13 | -1.16E+01 | -1.09E+01 | -1.37E+01 | -9.47E+00 |
| trans-Caffeic acid [apiosyl-(1-6)-glucosyl] ester Esi+3.0680006 | 3.49E-02 | -8.91E+00 | -3.95E+00 | -1.36E+01 | -4.23E+00 |
| L-Hexanoylcarnitine | 4.46E-03 | -1.01E+01 | -5.07E+00 | -1.42E+01 | -6.04E+00 |
| C29 H56 N9 O2 S Esi+14.777005 | 4.46E-02 | 8.72E+00 | 3.67E+00 | 3.86E+00 | 1.36E+01 |
| PC(20:4(8Z,11Z,14Z,17Z)/20:2(11Z,14Z)) Esi+14.852999 | 1.36E-04 | 4.87E+00 | 5.44E+00 | 3.10E+00 | 6.63E+00 |
| (3beta,24(241)Z)-24-Ethylidenelanost-9(11)-en-3-ol | 2.87E-02 | -9.14E+00 | -4.01E+00 | -1.38E+01 | -4.47E+00 |
| PG(18:0/18:1(11Z)) Esi+13.872997 | 7.71E-03 | -1.02E+01 | -4.90E+00 | -1.44E+01 | -5.89E+00 |
| 6-O-Oleuropeoylsucrose Esi+1.2700002 | 3.87E-02 | -8.85E+00 | -3.78E+00 | -1.37E+01 | -4.04E+00 |
| 8-Hydroxy-5,6-octadienoic acid | 1.61E-02 | -9.71E+00 | -4.98E+00 | -1.39E+01 | -5.55E+00 |
| FAD / Flavin adenine dinucleotide | 2.61E-05 | 2.28E+00 | 5.58E+00 | 1.48E+00 | 3.08E+00 |
| C18 H27 N9 O2 | 3.46E-02 | -8.35E+00 | -3.82E+00 | -1.28E+01 | -3.87E+00 |
| Pravastatin Esi+12.876004 | 2.62E-02 | -9.94E+00 | -4.17E+00 | -1.49E+01 | -5.02E+00 |
| Histidinyl-Asparagine Esi+13.158998 | 1.62E-02 | -1.02E+01 | -4.97E+00 | -1.46E+01 | -5.84E+00 |
| Abscisic alcohol 11-glucoside Esi+6.4130006 | 5.51E-03 | -1.02E+01 | -5.17E+00 | -1.43E+01 | -6.12E+00 |
| C30 H49 N2 O | 3.04E-02 | -9.17E+00 | -3.98E+00 | -1.39E+01 | -4.46E+00 |
| Tetradecanoyl-CoA Esi+8.708 | 6.34E-09 | -7.97E+00 | -7.76E+00 | -1.00E+01 | -5.94E+00 |
| C34 H34 Cl N O | 3.47E-02 | -9.12E+00 | -3.85E+00 | -1.40E+01 | -4.26E+00 |
| vesnarinone Esi+5.362 | 1.59E-02 | -9.22E+00 | -4.42E+00 | -1.35E+01 | -4.93E+00 |
| (S)-Piperazine-2-carboxamide Esi+7.0869985 | 2.20E-02 | -8.58E+00 | -4.21E+00 | -1.28E+01 | -4.38E+00 |
| Fleroxacin Esi+1.1229999 | 1.25E-02 | -9.11E+00 | -4.47E+00 | -1.33E+01 | -4.95E+00 |
| Aspartyl-Lysine | 4.67E-02 | -7.78E+00 | -3.95E+00 | -1.19E+01 | -3.62E+00 |
| C16 H12 N7 O2 | 2.40E-03 | -1.07E+01 | -5.55E+00 | -1.47E+01 | -6.75E+00 |
| Carindone Esi+14.208998 | 2.44E-02 | -9.22E+00 | -4.24E+00 | -1.37E+01 | -4.72E+00 |
| Apramycin Esi+10.878999 | 4.29E-02 | -8.52E+00 | -3.78E+00 | -1.32E+01 | -3.87E+00 |
| Spirolucidine Esi+14.024001 | 2.76E-03 | -1.18E+01 | -6.55E+00 | -1.56E+01 | -7.97E+00 |
| Hesperaline Esi+3.3590004 | 8.05E-05 | -1.19E+01 | -7.05E+00 | -1.53E+01 | -8.43E+00 |
| C27 H58 N7 O21 S2 | 3.41E-02 | -8.41E+00 | -3.88E+00 | -1.29E+01 | -3.95E+00 |
| Dehydroxyzyleuton | 2.44E-03 | -1.26E+01 | -6.68E+00 | -1.67E+01 | -8.60E+00 |
| 3'-Demethylstaurosporine Esi+4.802001 | 4.04E-03 | -9.58E+00 | -5.14E+00 | -1.34E+01 | -5.77E+00 |
| Sambacin Esi+5.3209996 | 1.94E-02 | -9.00E+00 | -4.82E+00 | -1.30E+01 | -5.02E+00 |
| cis-Zeatin 9-glucoside | 9.21E-03 | -1.02E+01 | -4.65E+00 | -1.46E+01 | -5.70E+00 |
| Glucosyl (2E,6E,10x)-10,11-dihydroxy-2,6-farnesadienoate Esi+6.5959983 | 4.11E-02 | -8.51E+00 | -3.81E+00 | -1.31E+01 | -3.90E+00 |
| C14 H8 N2 S3 | 1.65E-02 | -9.95E+00 | -4.96E+00 | -1.42E+01 | -5.67E+00 |
| PG(18:0/20:3(5Z,8Z,11Z)) Esi+13.849 | 2.19E-02 | -1.02E+01 | -4.19E+00 | -1.52E+01 | -5.20E+00 |
| Clerodendrin A Esi+8.289998 | 2.99E-02 | -9.46E+00 | -3.97E+00 | -1.43E+01 | -4.59E+00 |
| Etoposide Esi+13.395003 | 7.62E-03 | -1.06E+01 | -5.65E+00 | -1.46E+01 | -6.59E+00 |
| Cycloate | 1.94E-02 | -9.16E+00 | -4.24E+00 | -1.36E+01 | -4.73E+00 |
| Garcimangosone D Esi+2.7439997 | 1.24E-02 | -1.00E+01 | -4.78E+00 | -1.44E+01 | -5.66E+00 |
| Paeonilactone A Esi+9.421999 | 3.17E-03 | -1.18E+01 | -5.73E+00 | -1.61E+01 | -7.52E+00 |
| D-Pantothenoyl-L-cysteine | 1.66E-02 | -9.88E+00 | -4.94E+00 | -1.41E+01 | -5.62E+00 |
| (2Z,6E)-3,7,11,15,19-Pentamethyl-2,6-eicosadien-1-ol | 2.71E-02 | -9.36E+00 | -4.34E+00 | -1.39E+01 | -4.84E+00 |
| PC(22:1(13Z)/22:6(4Z,7Z,10Z,13Z,16Z,19Z)) Esi+13.938999 | 3.06E-02 | 3.72E+00 | 3.57E+00 | 1.67E+00 | 5.78E+00 |
| C34 H36 N22 O4 | 3.39E-02 | -8.69E+00 | -4.31E+00 | -1.30E+01 | -4.40E+00 |
| Drospirenone Esi+14.3289995 | 3.34E-02 | -6.92E+00 | -4.37E+00 | -1.03E+01 | -3.54E+00 |
| UDP-3-O-(3-hydroxytetradecanoyl)-N-acetylglucosamine Esi+8.002 | 1.07E-02 | -1.00E+01 | -4.60E+00 | -1.45E+01 | -5.56E+00 |
| Dimethamine Esi+13.328002 | 1.07E-02 | -9.54E+00 | -4.60E+00 | -1.38E+01 | -5.30E+00 |
| Benzoyl phosphate Esi+13.194 | 3.38E-02 | -8.57E+00 | -4.32E+00 | -1.28E+01 | -4.34E+00 |
| Senampeline A Esi+5.8180003 | 1.29E-03 | -1.26E+01 | -6.97E+00 | -1.64E+01 | -8.75E+00 |
| 5,10-Methylenetetrahydrofolate Esi+3.3429995 | 1.66E-13 | -9.36E+00 | -9.62E+00 | -1.13E+01 | -7.44E+00 |
| Tetradecanoyl-CoA Esi+8.345999 | 3.07E-06 | -6.87E+00 | -6.35E+00 | -9.00E+00 | -4.73E+00 |
| Propionyl-CoA | 2.25E-02 | 3.43E+00 | 3.72E+00 | 1.61E+00 | 5.26E+00 |
| Dimethyl succinate | 3.39E-02 | -8.50E+00 | -4.31E+00 | -1.27E+01 | -4.30E+00 |
| Dihydrochelirubine | 3.34E-02 | -8.53E+00 | -4.37E+00 | -1.27E+01 | -4.37E+00 |
| Thiobenzamide S,S-dioxide | 2.46E-03 | -1.23E+01 | -6.67E+00 | -1.62E+01 | -8.37E+00 |
| Methyl 3-(2,3-dihydroxy-3-methylbutyl)-4-hydroxybenzoate Esi+2.6910005 | 8.17E-03 | -1.02E+01 | -4.71E+00 | -1.46E+01 | -5.76E+00 |
| alpha-L-Rhamnopyranosyl-(1-3)-alpha-D-galactopyranosyl-(1-3)-L-fucose Esi+3.256 | 3.47E-03 | -1.07E+01 | -5.19E+00 | -1.49E+01 | -6.49E+00 |
| Flumazenil | 2.51E-02 | -8.49E+00 | -4.20E+00 | -1.27E+01 | -4.31E+00 |
| PC(22:4(7Z,10Z,13Z,16Z)/22:6(4Z,7Z,10Z,13Z,16Z,19Z)) | 8.20E-06 | 5.17E+00 | 6.05E+00 | 3.49E+00 | 6.86E+00 |
| Z-Tyr-Leu-NHOH Esi+5.2540007 | 6.92E-03 | -9.44E+00 | -5.03E+00 | -1.33E+01 | -5.57E+00 |
| Quetiapine | 3.57E-02 | -9.01E+00 | -3.83E+00 | -1.38E+01 | -4.18E+00 |
| Benzyl gentiobioside Esi+4.1930003 | 1.94E-02 | -9.24E+00 | -4.22E+00 | -1.37E+01 | -4.76E+00 |
| C14 H29 N O S2 | 3.35E-02 | -8.07E+00 | -4.33E+00 | -1.20E+01 | -4.10E+00 |
| C31 H43 Cl O S | 2.75E-02 | -9.45E+00 | -4.13E+00 | -1.42E+01 | -4.74E+00 |
| Cucurbitacin P Esi+14.382001 | 1.48E-02 | -9.55E+00 | -4.37E+00 | -1.40E+01 | -5.09E+00 |
| PE(22:4(7Z,10Z,13Z,16Z)/P-18:0) | 1.31E-02 | -5.79E+00 | -4.05E+00 | -8.64E+00 | -2.95E+00 |
| Mahuannin D | 3.43E-02 | -8.44E+00 | -4.01E+00 | -1.28E+01 | -4.06E+00 |
| Purpurin | 2.48E-03 | -1.22E+01 | -6.66E+00 | -1.62E+01 | -8.33E+00 |
| PC(14:0/14:0) Esi+14.581999 | 1.74E-05 | 5.47E+00 | 5.84E+00 | 3.63E+00 | 7.31E+00 |
| Kentsin | 3.34E-02 | -8.62E+00 | -4.36E+00 | -1.28E+01 | -4.41E+00 |
| BQ 518 | 2.88E-03 | -1.06E+01 | -5.29E+00 | -1.47E+01 | -6.50E+00 |
| PG(18:0/22:5(4Z,7Z,10Z,13Z,16Z)) Esi+13.856002 | 3.34E-02 | -8.75E+00 | -3.92E+00 | -1.33E+01 | -4.16E+00 |
| Garcimangosone D Esi+4.632 | 3.34E-02 | -8.66E+00 | -4.37E+00 | -1.29E+01 | -4.44E+00 |
| Fluocortolone | 1.10E-02 | -9.24E+00 | -4.55E+00 | -1.34E+01 | -5.09E+00 |
| (S)-Multifidol 2-[apiosyl-(1-6)-glucoside] Esi+3.841 :1 | 3.34E-02 | -9.17E+00 | -3.87E+00 | -1.40E+01 | -4.33E+00 |
| Sorbitan palmitate Esi+13.377 | 3.64E-02 | -1.01E+01 | -3.84E+00 | -1.55E+01 | -4.69E+00 |
| 3'-Prenylapigenin 7-[rhamnosyl-(1-6)-glucoside] Esi+7.1900005 | 2.67E-03 | -1.02E+01 | -5.36E+00 | -1.41E+01 | -6.30E+00 |
| Methoxybrassenin B | 7.52E-03 | -1.08E+01 | -5.69E+00 | -1.48E+01 | -6.76E+00 |
| C20 H31 Cl N4 O4 | 4.63E-02 | -8.06E+00 | -3.63E+00 | -1.26E+01 | -3.53E+00 |
| trans-Brassidic acid Esi+16.070004 | 7.52E-03 | -1.05E+01 | -5.69E+00 | -1.44E+01 | -6.57E+00 |
| Tetrahydroaldosterone-3-glucuronide Esi+7.878999 | 2.23E-02 | -7.84E+00 | -4.12E+00 | -1.17E+01 | -3.95E+00 |
| Sildenafil Esi+7.826001 | 3.46E-02 | -7.28E+00 | -4.28E+00 | -1.09E+01 | -3.66E+00 |
| Oryzanol A Esi+13.929999 | 4.21E-02 | -8.42E+00 | -3.78E+00 | -1.30E+01 | -3.83E+00 |
| dCMP / 2'-Deoxycytidine 5'-monophosphate Esi+2.247 | 1.24E-02 | -1.04E+01 | -4.76E+00 | -1.49E+01 | -5.86E+00 |
| 7-Epiloganin tetraacetate Esi+4.777 | 3.30E-02 | -8.47E+00 | -4.11E+00 | -1.28E+01 | -4.18E+00 |
| C4 H6 Cl N2 S2 | 2.76E-03 | -1.08E+01 | -6.54E+00 | -1.43E+01 | -7.28E+00 |
| (7'R)-(+)-Lyoniresinol 9'-glucoside Esi+5.264 | 3.85E-02 | -9.16E+00 | -3.76E+00 | -1.41E+01 | -4.16E+00 |
| 2-(2-Methylpropoxy)naphthalene | 2.53E-03 | -1.15E+01 | -6.63E+00 | -1.53E+01 | -7.84E+00 |
| 1,2-Di-(9Z,12Z,15Z-octadecatrienoyl)-3-(Galactosyl-alpha-1-6-Galactosyl-beta-1)-glycerol Esi+14.911002 :1 | 1.14E-02 | -1.17E+01 | -5.12E+00 | -1.66E+01 | -6.89E+00 |
| UDP-3-O-(3-hydroxytetradecanoyl)-N-acetylglucosamine Esi+7.7399983 | 3.91E-02 | -8.59E+00 | -3.73E+00 | -1.33E+01 | -3.88E+00 |
| (S)-2-Hydroxy-2-phenylacetonitrile O-[b-D-apiosyl-(1-2)-b-D-glucoside] | 2.33E-02 | -8.30E+00 | -4.10E+00 | -1.24E+01 | -4.16E+00 |
| UK 78282 Esi+9.800001 | 3.12E-02 | -8.66E+00 | -4.18E+00 | -1.30E+01 | -4.34E+00 |
| PC(20:4(8Z,11Z,14Z,17Z)/20:2(11Z,14Z)) Esi+14.457 | 3.35E-08 | 6.30E+00 | 7.28E+00 | 4.59E+00 | 8.01E+00 |
| 7(14)-Bisabolene-2,3,10,11-tetrol Esi+9.385 | 2.79E-02 | -9.71E+00 | -4.03E+00 | -1.46E+01 | -4.78E+00 |
| C18 H29 N15 | 1.39E-02 | -9.03E+00 | -4.65E+00 | -1.31E+01 | -5.01E+00 |
| C38 H30 N27 O | 3.50E-02 | -8.04E+00 | -3.93E+00 | -1.23E+01 | -3.80E+00 |
| PC(14:0/14:0) Esi+14.581999 :1 | 2.09E-08 | 6.59E+00 | 7.25E+00 | 4.80E+00 | 8.39E+00 |
| PC(20:4(8Z,11Z,14Z,17Z)/20:2(11Z,14Z)) Esi+14.371998 | 2.29E-02 | 3.55E+00 | 3.69E+00 | 1.65E+00 | 5.44E+00 |
| Justicidin B | 7.52E-03 | -1.05E+01 | -5.70E+00 | -1.45E+01 | -6.60E+00 |
| Icariin Esi+6.8559995 | 3.34E-02 | -8.30E+00 | -4.36E+00 | -1.24E+01 | -4.24E+00 |
| PE(14:0/14:1(9Z)) Esi+15.372 | 7.93E-03 | -6.43E+00 | -4.28E+00 | -9.42E+00 | -3.44E+00 |
| C36 H56 O3 Esi+16.069 | 3.34E-02 | -8.99E+00 | -4.03E+00 | -1.36E+01 | -4.37E+00 |
| C30 H47 N O2 | 1.47E-02 | -1.08E+01 | -4.38E+00 | -1.58E+01 | -5.76E+00 |
| PE(14:0/P-18:0) Esi+14.229002 | 7.71E-03 | -6.62E+00 | -4.26E+00 | -9.71E+00 | -3.54E+00 |
| (3beta,17alpha,23R)-17,23-Epoxy-3,29-dihydroxy-27-norlanost-8-ene-15,24-dione Esi+15.899996 | 4.54E-02 | -7.63E+00 | -3.76E+00 | -1.18E+01 | -3.43E+00 |
| PC(14:0/14:0) Esi+15.380998 | 4.36E-04 | 4.28E+00 | 5.10E+00 | 2.63E+00 | 5.94E+00 |
| C31 H14 N15 O9 | 7.57E-03 | -1.05E+01 | -5.66E+00 | -1.45E+01 | -6.55E+00 |
| Eremosulphoxinolide A | 3.32E-03 | -1.01E+01 | -5.25E+00 | -1.40E+01 | -6.14E+00 |
| 1,2-Di-(9Z,12Z,15Z-octadecatrienoyl)-3-(Galactosyl-alpha-1-6-Galactosyl-beta-1)-glycerol Esi+14.911002 :2 | 1.14E-02 | -1.17E+01 | -5.12E+00 | -1.66E+01 | -6.89E+00 |
| UDP-3-O-(3-hydroxytetradecanoyl)-N-acetylglucosamine Esi+8.126002 | 4.21E-02 | -8.32E+00 | -3.68E+00 | -1.29E+01 | -3.71E+00 |
| PG(18:0/18:1(11Z)) Esi+13.877002 | 3.34E-02 | -8.70E+00 | -3.90E+00 | -1.33E+01 | -4.12E+00 |
| Antirrhinoside Esi+3.269 | 1.74E-02 | -9.44E+00 | -4.81E+00 | -1.36E+01 | -5.28E+00 |
| 7-Epiloganin tetraacetate Esi+4.8009996 | 3.28E-02 | -8.47E+00 | -4.11E+00 | -1.28E+01 | -4.18E+00 |
| 6-O-p-Coumaroyl-D-glucose Esi+3.1129994 | 1.67E-02 | -1.00E+01 | -4.94E+00 | -1.43E+01 | -5.68E+00 |
| C19 H25 N5 O | 1.07E-02 | -1.01E+01 | -4.66E+00 | -1.45E+01 | -5.62E+00 |
| PE(22:5(7Z,10Z,13Z,16Z,19Z)/P-16:0) Esi+15.088999 | 6.70E-03 | -4.19E+00 | -4.56E+00 | -6.04E+00 | -2.34E+00 |
| Biotripyrrin-b Esi+1.1889997 | 4.19E-02 | -7.70E+00 | -3.80E+00 | -1.19E+01 | -3.51E+00 |
| LysoPC(20:3(5Z,8Z,11Z)) Esi+6.055 | 4.96E-02 | -5.36E+00 | -3.43E+00 | -8.49E+00 | -2.23E+00 |
| Gentiobiosyl 2-methyl-6-oxo-2E,4E-heptadienoate Esi+3.0079997 | 1.37E-02 | -9.30E+00 | -4.65E+00 | -1.34E+01 | -5.16E+00 |
| 10-formyldihydrofolate Esi+3.4119997 | 2.81E-03 | -1.16E+01 | -5.39E+00 | -1.60E+01 | -7.21E+00 |
| UDP-3-O-(3-hydroxytetradecanoyl)-N-acetylglucosamine Esi+7.6080008 | 3.43E-02 | -8.70E+00 | -3.83E+00 | -1.34E+01 | -4.05E+00 |
| Antirrhinoside Esi+3.1020005 | 4.46E-03 | -1.06E+01 | -5.06E+00 | -1.49E+01 | -6.32E+00 |
| LysoPC(22:1(13Z)) Esi+13.822001 | 1.53E-02 | -6.77E+00 | -4.07E+00 | -1.01E+01 | -3.44E+00 |
| Isoferuloyl C1-glucuronide Esi+3.8690002 | 3.15E-02 | -8.99E+00 | -3.93E+00 | -1.37E+01 | -4.32E+00 |
| 3beta-Acetoxy-19alpha-hydroxy-12-ursene Esi+16.446 | 3.96E-02 | -7.83E+00 | -3.82E+00 | -1.21E+01 | -3.60E+00 |
| Valerosidatum Esi+8.886998 | 1.14E-02 | -9.21E+00 | -4.67E+00 | -1.33E+01 | -5.15E+00 |
| Congmuyenoside B | 3.34E-02 | -8.11E+00 | -3.87E+00 | -1.24E+01 | -3.82E+00 |
| Pyranocyanin B Esi+3.4809992 | 3.18E-02 | -8.31E+00 | -4.03E+00 | -1.26E+01 | -4.06E+00 |
| C18 H39 N O Esi+13.787999 | 3.79E-02 | 9.69E+00 | 3.81E+00 | 4.46E+00 | 1.49E+01 |
| Geldanamycin Esi+6.124 | 2.75E-02 | -8.84E+00 | -4.01E+00 | -1.33E+01 | -4.34E+00 |
| Stachyoside A Esi+3.35 | 3.34E-02 | -8.42E+00 | -4.37E+00 | -1.25E+01 | -4.31E+00 |
| (+)-Muscarine Esi+13.845 | 1.65E-02 | -9.39E+00 | -4.96E+00 | -1.34E+01 | -5.36E+00 |
| Ergocornine Esi+6.64 | 5.53E-03 | -9.63E+00 | -5.13E+00 | -1.35E+01 | -5.76E+00 |
| Ximelagatran Esi+14.786 | 3.34E-02 | -8.93E+00 | -4.36E+00 | -1.33E+01 | -4.56E+00 |
| Pseudaminic acid Esi+10.492999 | 3.34E-02 | -8.40E+00 | -4.38E+00 | -1.25E+01 | -4.31E+00 |
| Artonin Q Esi+4.8930006 | 1.70E-02 | -9.19E+00 | -4.92E+00 | -1.32E+01 | -5.21E+00 |
| 3'-Demethylstaurosporine Esi+4.7979994 | 2.70E-02 | -8.49E+00 | -4.15E+00 | -1.27E+01 | -4.27E+00 |
| Verbasoside Esi+5.9199996 | 3.45E-02 | -8.65E+00 | -4.00E+00 | -1.31E+01 | -4.16E+00 |
| Chivosazole F Esi+13.904002 | 7.62E-03 | -9.99E+00 | -5.64E+00 | -1.38E+01 | -6.21E+00 |
| PC(15:0/15:0) Esi+14.002998 | 9.24E-07 | -5.59E+00 | -6.56E+00 | -7.27E+00 | -3.91E+00 |
| Androcymbine Esi+6.9779987 | 3.11E-02 | -9.69E+00 | -3.97E+00 | -1.47E+01 | -4.70E+00 |
| C13 H31 N4 O4 S | 1.18E-02 | -9.62E+00 | -4.58E+00 | -1.39E+01 | -5.31E+00 |
| Tetracenomycin B1 | 1.62E-02 | -8.79E+00 | -4.97E+00 | -1.26E+01 | -5.03E+00 |
| PC(18:3(9Z,12Z,15Z)/18:2(9Z,12Z)) | 3.38E-02 | 2.44E+00 | 3.50E+00 | 1.06E+00 | 3.81E+00 |
| PC(16:0/15:0) | 8.01E-07 | -6.08E+00 | -6.51E+00 | -7.92E+00 | -4.24E+00 |
| Tetrahydrofolic acid Esi+5.83 | 7.45E-06 | -4.76E+00 | -6.13E+00 | -6.29E+00 | -3.23E+00 |
| Dihydrofolic acid Esi+3.2449992 | 3.18E-07 | -9.82E+00 | -7.70E+00 | -1.24E+01 | -7.27E+00 |
| Gentianose Esi+0.81299984 | 2.56E-02 | -8.83E+00 | -4.56E+00 | -1.29E+01 | -4.72E+00 |
| Jadomycin A Esi+3.2750003 | 7.61E-03 | -1.01E+01 | -5.65E+00 | -1.39E+01 | -6.27E+00 |
| dolichyl phosphate Esi+12.97 | 3.34E-02 | -7.75E+00 | -4.30E+00 | -1.16E+01 | -3.94E+00 |
| Istamycin A1 Esi+8.892999 | 4.29E-02 | -8.46E+00 | -3.81E+00 | -1.31E+01 | -3.86E+00 |
| Rumexoside | 4.92E-04 | -1.12E+01 | -6.15E+00 | -1.49E+01 | -7.45E+00 |
| D-Pinitol 2-O-alpha-D-Galactopyranosyl | 3.35E-02 | -7.92E+00 | -3.84E+00 | -1.21E+01 | -3.71E+00 |
| C5 H9 Cl N4 O | 1.54E-04 | -1.29E+01 | -6.70E+00 | -1.69E+01 | -8.99E+00 |
| PC(15:0/14:1(9Z)) Esi+13.906001 | 6.34E-09 | -6.58E+00 | -7.52E+00 | -8.31E+00 | -4.86E+00 |
| PE(20:4(8Z,11Z,14Z,17Z)/20:1(11Z)) Esi+15.210001 | 4.20E-06 | -8.30E+00 | -6.53E+00 | -1.08E+01 | -5.78E+00 |
| Indeloxazine | 7.62E-03 | -9.76E+00 | -5.64E+00 | -1.34E+01 | -6.07E+00 |
| 2-N,6-N-Bis(2,3-dihydroxybenzoyl)-L-lysine Esi+1.0500001 | 3.34E-02 | -7.92E+00 | -4.03E+00 | -1.20E+01 | -3.84E+00 |
| PE(20:4(8Z,11Z,14Z,17Z)/22:2(13Z,16Z)) | 6.34E-09 | -9.99E+00 | -8.11E+00 | -1.24E+01 | -7.55E+00 |
| Lymecycline Esi+6.4840007 | 1.32E-02 | -9.40E+00 | -4.72E+00 | -1.35E+01 | -5.27E+00 |
| Gibberellin A97 Esi+1.1900003 | 4.42E-02 | -7.99E+00 | -4.05E+00 | -1.22E+01 | -3.80E+00 |
| N-Feruloylglycine Esi+5.3649993 | 1.70E-02 | -9.90E+00 | -4.83E+00 | -1.42E+01 | -5.55E+00 |
| PC(18:3(9Z,12Z,15Z)/18:2(9Z,12Z)) Esi+13.871001 | 5.57E-03 | -4.16E+00 | -4.28E+00 | -6.08E+00 | -2.24E+00 |
| Tetradecanoyl-CoA Esi+8.551001 | 5.03E-06 | -6.81E+00 | -6.24E+00 | -8.97E+00 | -4.66E+00 |
| C15 H26 N O6 | 1.33E-02 | -9.52E+00 | -5.02E+00 | -1.35E+01 | -5.51E+00 |
| All trans decaprenyl diphosphate Esi+13.843001 | 3.40E-02 | -8.29E+00 | -3.88E+00 | -1.27E+01 | -3.91E+00 |
| Tetradecanoyl-CoA Esi+8.346 | 3.35E-08 | -7.66E+00 | -7.33E+00 | -9.73E+00 | -5.60E+00 |
| Amprenavir Esi+6.475999 | 3.34E-02 | -6.78E+00 | -4.35E+00 | -1.01E+01 | -3.46E+00 |
| (1R*,2R*,4R*,8S*)-p-Menthane-1,2,8,9-tetrol 9-glucoside Esi+1.2399999 | 3.80E-02 | -8.06E+00 | -3.74E+00 | -1.25E+01 | -3.66E+00 |
| Dolichyl b-D-glucosyl phosphate Esi+4.909 | 3.46E-02 | -8.22E+00 | -3.95E+00 | -1.25E+01 | -3.91E+00 |
| Phenylethyl primeveroside Esi+1.2299997 | 1.66E-02 | -8.68E+00 | -4.44E+00 | -1.27E+01 | -4.64E+00 |
| PC(20:5(5Z,8Z,11Z,14Z,17Z)/14:0) | 2.23E-06 | -5.47E+00 | -6.32E+00 | -7.17E+00 | -3.76E+00 |
| Landomycin D Esi+10.948999 | 3.26E-02 | -8.20E+00 | -4.11E+00 | -1.23E+01 | -4.05E+00 |
| PC(14:0/16:1(9Z)) | 3.17E-03 | 4.29E+00 | 4.48E+00 | 2.40E+00 | 6.17E+00 |
| Diphyllin | 1.65E-02 | -8.65E+00 | -4.32E+00 | -1.27E+01 | -4.55E+00 |
| PC(14:0/14:0) Esi+14.563999 | 2.38E-06 | 5.60E+00 | 6.29E+00 | 3.84E+00 | 7.35E+00 |
| 3-Caffeoylpelargonidin 5-glucoside Esi+5.658999 | 3.35E-02 | -8.14E+00 | -3.91E+00 | -1.24E+01 | -3.85E+00 |
| Myricanene B 5-[arabinosyl-(1-6)-glucoside] Esi+7.39 | 3.12E-02 | -8.56E+00 | -4.17E+00 | -1.28E+01 | -4.29E+00 |
| Ustiloxin C | 3.18E-02 | -8.99E+00 | -3.95E+00 | -1.36E+01 | -4.33E+00 |
| PC(18:0/22:5(7Z,10Z,13Z,16Z,19Z)) | 9.10E-05 | -5.12E+00 | -5.48E+00 | -6.96E+00 | -3.28E+00 |
| Deoxyloganin tetraacetate Esi+5.2979984 | 2.01E-02 | -8.82E+00 | -4.19E+00 | -1.31E+01 | -4.51E+00 |
| Morfamquat Esi+6.4700007 | 3.04E-02 | -8.18E+00 | -4.06E+00 | -1.23E+01 | -4.03E+00 |
| Dimepiperate Esi+13.976999 | 3.34E-02 | -8.16E+00 | -4.38E+00 | -1.21E+01 | -4.19E+00 |
| PC(20:4(5Z,8Z,11Z,14Z)/22:6(4Z,7Z,10Z,13Z,16Z,19Z)) | 2.81E-07 | -5.01E+00 | -7.06E+00 | -6.41E+00 | -3.60E+00 |
| (E,E)-4,4''-Bi(N-4-hydroxycinnamoylserotonin) Esi+6.803001 | 2.80E-02 | -8.59E+00 | -4.02E+00 | -1.30E+01 | -4.22E+00 |
| 22?-Hydroxy-5?-campestan-3-one Esi+15.787998 | 1.43E-02 | -9.89E+00 | -4.62E+00 | -1.43E+01 | -5.45E+00 |
| C19 H37 Cl N3 O2 S4 | 4.08E-02 | -7.82E+00 | -3.81E+00 | -1.21E+01 | -3.58E+00 |
| C23 H2 O24 | 3.34E-02 | -7.89E+00 | -4.38E+00 | -1.17E+01 | -4.04E+00 |
| Eugenol O-[3,4,5-Trihydroxybenzoyl-(-6)-b-D-glucopyranoside] | 1.33E-02 | -9.10E+00 | -4.43E+00 | -1.33E+01 | -4.91E+00 |
| Icariin Esi+6.8559995 :1 | 3.34E-02 | -8.42E+00 | -4.36E+00 | -1.25E+01 | -4.31E+00 |
| Aralionine A Esi+5.989999 | 3.34E-02 | -7.91E+00 | -4.06E+00 | -1.20E+01 | -3.86E+00 |
| 4-Megastigmene-6a,9R-diol 9-[apiosyl-(1-6)-glucoside] Esi+6.738999 | 1.96E-02 | -8.67E+00 | -4.18E+00 | -1.29E+01 | -4.44E+00 |
| Dihydroceramide Esi+12.921001 | 2.99E-02 | 8.83E+00 | 3.99E+00 | 4.30E+00 | 1.34E+01 |
| PE(18:4(6Z,9Z,12Z,15Z)/18:4(6Z,9Z,12Z,15Z)) Esi+14.191001 | 2.39E-02 | -4.20E+00 | -3.79E+00 | -6.41E+00 | -2.00E+00 |
| C26 O22 | 3.34E-02 | -7.67E+00 | -4.38E+00 | -1.14E+01 | -3.93E+00 |
| Bay-K-8644 Esi+1.045 | 3.34E-02 | -7.69E+00 | -4.38E+00 | -1.14E+01 | -3.95E+00 |
| Halistanol sulfate Esi+8.138 | 3.34E-02 | -7.68E+00 | -4.38E+00 | -1.14E+01 | -3.94E+00 |
| Licoricesaponin C2 Esi+14.0720005 | 3.39E-02 | 8.73E+00 | 4.00E+00 | 4.20E+00 | 1.33E+01 |
| 100-2 Esi+10.135001 | 3.34E-02 | -7.77E+00 | -4.37E+00 | -1.16E+01 | -3.98E+00 |
| C13 H30 Cl N6 S2 | 3.34E-02 | -8.35E+00 | -4.06E+00 | -1.26E+01 | -4.07E+00 |
| Coproporphyrin I Esi+9.671 | 1.61E-02 | 9.35E+00 | 4.51E+00 | 5.06E+00 | 1.36E+01 |
| Pheophytin a Esi+15.421998 | 4.53E-03 | 1.29E+01 | 5.92E+00 | 8.32E+00 | 1.76E+01 |
| PC(22:5(4Z,7Z,10Z,13Z,16Z)/22:6(4Z,7Z,10Z,13Z,16Z,19Z)) Esi+16.175001 | 3.12E-06 | 5.93E+00 | 6.25E+00 | 4.06E+00 | 7.81E+00 |
| C9 H17 N O4 | 1.61E-02 | 1.05E+01 | 4.99E+00 | 6.00E+00 | 1.49E+01 |
| Satratoxin H Esi+12.554999 | 3.38E-02 | 8.21E+00 | 3.90E+00 | 3.88E+00 | 1.25E+01 |
| DG(14:0/14:0/0:0) Esi+16.031002 | 3.97E-03 | -6.43E+00 | -4.59E+00 | -9.23E+00 | -3.64E+00 |
| PE(18:1(9Z)/14:0) Esi+12.898999 | 1.94E-06 | 8.86E+00 | 6.76E+00 | 6.25E+00 | 1.15E+01 |
| Coproporphyrin I Esi+9.669001 | 3.63E-02 | 8.33E+00 | 3.92E+00 | 3.93E+00 | 1.27E+01 |
| Argenteane Esi+13.371001 | 2.87E-02 | 8.81E+00 | 4.05E+00 | 4.34E+00 | 1.33E+01 |
| PC(22:5(7Z,10Z,13Z,16Z,19Z)/22:6(4Z,7Z,10Z,13Z,16Z,19Z)) | 4.08E-02 | 3.49E+00 | 3.38E+00 | 1.45E+00 | 5.53E+00 |
| PE(14:0/P-18:1(9Z)) | 1.47E-02 | -5.25E+00 | -4.03E+00 | -7.85E+00 | -2.65E+00 |
| Pravastatin Esi+13.206999 | 1.71E-02 | 8.91E+00 | 4.29E+00 | 4.66E+00 | 1.32E+01 |
| 34a-Deoxy-rifamycin W Esi+14.601002 | 1.51E-02 | 9.95E+00 | 4.39E+00 | 5.31E+00 | 1.46E+01 |
| PE(14:0/P-18:1(9Z)) Esi+12.721 | 2.81E-03 | -6.24E+00 | -4.75E+00 | -8.86E+00 | -3.62E+00 |
| C24 H55 Cl N16 S | 3.81E-02 | -8.77E+00 | -3.78E+00 | -1.35E+01 | -4.01E+00 |
| N-Acetyl-N6,O-didemethylpuromycin-5'-phosphate Esi+5.9010005 | 3.34E-02 | -7.69E+00 | -4.03E+00 | -1.17E+01 | -3.73E+00 |
| PE(15:0/16:0) Esi+15.602 | 3.96E-08 | 9.64E+00 | 7.73E+00 | 7.16E+00 | 1.21E+01 |
| Aralionine A Esi+10.856002 | 2.14E-02 | 9.02E+00 | 4.23E+00 | 4.63E+00 | 1.34E+01 |
| C49 H53 Cl N6 O2 | 3.34E-02 | 9.32E+00 | 4.38E+00 | 4.79E+00 | 1.39E+01 |
| PE(14:0/18:4(6Z,9Z,12Z,15Z)) Esi+12.832 | 3.55E-02 | 4.71E+00 | 3.51E+00 | 2.05E+00 | 7.38E+00 |
| PC(14:0/14:0) Esi+12.872 | 1.11E-03 | 3.56E+00 | 4.85E+00 | 2.11E+00 | 5.01E+00 |
| PE(14:0/16:1(9Z)) Esi+9.455001 | 1.69E-02 | 4.39E+00 | 3.96E+00 | 2.17E+00 | 6.60E+00 |
| Pheophytin a Esi+15.421998 :1 | 3.12E-02 | 1.02E+01 | 4.06E+00 | 5.04E+00 | 1.55E+01 |
| C13 H26 N2 O2 | 3.25E-02 | -9.42E+00 | -4.14E+00 | -1.42E+01 | -4.68E+00 |
| PE(14:0/P-18:0) Esi+13.75 | 2.79E-02 | 4.98E+00 | 3.69E+00 | 2.30E+00 | 7.66E+00 |
| Imperialine Esi+15.822002 | 4.21E-02 | -1.05E+01 | -3.72E+00 | -1.63E+01 | -4.72E+00 |
| PI(20:2(11Z,14Z)/16:0) Esi+13.548002 | 1.24E-02 | 6.52E+00 | 4.25E+00 | 3.43E+00 | 9.60E+00 |
| 4,4'-Sulfonyldiphenol | 7.62E-03 | -1.01E+01 | -5.64E+00 | -1.39E+01 | -6.26E+00 |
| Tamoxifen Esi+8.518999 | 1.39E-02 | -9.15E+00 | -4.41E+00 | -1.34E+01 | -4.91E+00 |
| PC(22:0/20:4(5Z,8Z,11Z,14Z)) | 9.45E-04 | 4.54E+00 | 4.87E+00 | 2.70E+00 | 6.38E+00 |
| 6?-Glucuronosylhyodeoxycholate Esi+14.222997 | 3.43E-02 | 8.33E+00 | 4.00E+00 | 4.00E+00 | 1.27E+01 |
| PE(14:0/14:1(9Z)) Esi+13.345 | 1.11E-02 | -4.93E+00 | -4.20E+00 | -7.27E+00 | -2.58E+00 |
| PC(20:1(11Z)/18:3(6Z,9Z,12Z)) | 4.60E-03 | -3.70E+00 | -4.35E+00 | -5.37E+00 | -2.02E+00 |
| Talinumoside I Esi+14.052 | 3.03E-02 | 1.04E+01 | 3.98E+00 | 5.03E+00 | 1.57E+01 |
| PI(22:4(10Z,13Z,16Z,19Z)/18:0) Esi+14.910002 | 3.14E-02 | -6.76E+00 | -3.70E+00 | -1.04E+01 | -3.11E+00 |
| Pelargonidin 3-O-rutinoside Esi+6.3180003 | 3.85E-02 | -8.53E+00 | -3.79E+00 | -1.32E+01 | -3.91E+00 |
| Talinumoside I Esi+14.048997 | 3.05E-02 | 1.03E+01 | 3.98E+00 | 5.01E+00 | 1.56E+01 |
| Dalfopristin Esi+14.035003 | 2.55E-02 | 1.04E+01 | 4.20E+00 | 5.27E+00 | 1.55E+01 |
| Licoagrodin Esi+8.14 | 3.34E-02 | -7.52E+00 | -4.38E+00 | -1.12E+01 | -3.86E+00 |
| PC(20:4(8Z,11Z,14Z,17Z)/20:2(11Z,14Z)) Esi+14.485999 | 2.48E-11 | 7.32E+00 | 8.82E+00 | 5.68E+00 | 8.96E+00 |
| PI(20:2(11Z,14Z)/18:2(9Z,12Z)) Esi+14.567996 | 1.56E-02 | 7.15E+00 | 4.04E+00 | 3.61E+00 | 1.07E+01 |
| 2,3-dinor-8-iso-PGF2a Esi+10.306 | 3.34E-02 | 8.66E+00 | 4.10E+00 | 4.26E+00 | 1.31E+01 |
| Pheophytin a Esi+15.014005 | 1.90E-02 | 1.01E+01 | 4.20E+00 | 5.17E+00 | 1.49E+01 |
| PC(14:1(9Z)/20:5(5Z,8Z,11Z,14Z,17Z)) Esi+15.014 | 8.92E-03 | 3.00E+00 | 4.11E+00 | 1.56E+00 | 4.43E+00 |
| Lyciumoside VIII Esi+14.554 | 3.54E-02 | 7.66E+00 | 3.97E+00 | 3.65E+00 | 1.17E+01 |
| Elaidic carnitine Esi+11.779 | 1.65E-02 | 1.09E+01 | 4.96E+00 | 6.23E+00 | 1.56E+01 |
| Cucurbitacin P Esi+10.166 | 3.34E-02 | 8.56E+00 | 4.06E+00 | 4.17E+00 | 1.29E+01 |
| PC(20:3(8Z,11Z,14Z)/15:0) Esi+13.214998 | 3.48E-03 | 3.17E+00 | 4.49E+00 | 1.77E+00 | 4.56E+00 |
| Acetoacetyl-CoA | 1.23E-09 | 5.09E+00 | 8.05E+00 | 3.84E+00 | 6.34E+00 |
| PA(16:0/16:0)[rac] Esi+15.643 | 1.61E-04 | 5.77E+00 | 5.62E+00 | 3.73E+00 | 7.81E+00 |
| Acetoacetyl-CoA Esi+2.959 | 1.21E-09 | 4.87E+00 | 8.06E+00 | 3.68E+00 | 6.06E+00 |
| Testosterone glucuronide Esi+13.273 | 3.32E-02 | 8.06E+00 | 4.09E+00 | 3.96E+00 | 1.22E+01 |
| PC(18:3(9Z,12Z,15Z)/18:2(9Z,12Z)) Esi+13.714998 | 2.99E-11 | 6.70E+00 | 8.73E+00 | 5.18E+00 | 8.21E+00 |
| C25 N O16 | 1.65E-02 | -9.61E+00 | -4.95E+00 | -1.37E+01 | -5.47E+00 |
| C21 H2 N O19 | 1.65E-02 | -9.33E+00 | -4.95E+00 | -1.34E+01 | -5.32E+00 |
| (S)-N-[3-(3,4-Methylenedioxyphenyl)-2-(mercaptomethyl)-1-oxoprolyl]glycine | 3.34E-02 | -8.22E+00 | -4.35E+00 | -1.22E+01 | -4.20E+00 |
| PC(24:0/P-18:1(11Z)) | 6.47E-04 | 3.67E+00 | 5.02E+00 | 2.23E+00 | 5.12E+00 |
| PC(20:4(8Z,11Z,14Z,17Z)/20:2(11Z,14Z)) Esi+15.358001 | 3.89E-02 | 2.79E+00 | 3.41E+00 | 1.17E+00 | 4.40E+00 |
| PC(14:1(9Z)/22:6(4Z,7Z,10Z,13Z,16Z,19Z)) Esi+14.244001 | 1.27E-11 | 6.21E+00 | 9.26E+00 | 4.88E+00 | 7.54E+00 |
| PC(22:0/20:4(5Z,8Z,11Z,14Z)) Esi+13.863999 | 3.05E-03 | 2.91E+00 | 4.51E+00 | 1.64E+00 | 4.18E+00 |
| PC(14:0/14:0) Esi+12.678 | 8.26E-04 | 2.50E+00 | 5.04E+00 | 1.51E+00 | 3.48E+00 |
| PC(20:4(8Z,11Z,14Z,17Z)/20:2(11Z,14Z)) Esi+15.307 | 2.67E-03 | 3.64E+00 | 4.61E+00 | 2.08E+00 | 5.20E+00 |
| PC(20:4(8Z,11Z,14Z,17Z)/20:2(11Z,14Z)) Esi+14.901 | 3.18E-02 | 2.99E+00 | 3.57E+00 | 1.34E+00 | 4.65E+00 |
| all-trans-Undecaprenyl phosphate Esi+14.216002 | 3.65E-02 | 8.21E+00 | 3.90E+00 | 3.85E+00 | 1.26E+01 |
| PC(18:1(11Z)/18:3(6Z,9Z,12Z)) | 1.96E-05 | 4.31E+00 | 6.04E+00 | 2.89E+00 | 5.72E+00 |
| PC(20:3(5Z,8Z,11Z)/20:1(11Z)) Esi+14.201997 | 5.02E-03 | -3.65E+00 | -4.32E+00 | -5.32E+00 | -1.98E+00 |
